# A Randomized, Double-Blind, Placebo-Controlled, Phase II Study Assessing Safety, Tolerability, and Efficacy of Bryostatin in the Treatment of Moderately Severe to Severe Alzheimer’s Disease

**DOI:** 10.3233/JAD-180759

**Published:** 2019-01-22

**Authors:** Martin R. Farlow, Richard E. Thompson, Lee-Jen Wei, Alan J. Tuchman, Elaine Grenier, David Crockford, Susanne Wilke, Jeffrey Benison, Daniel L. Alkon

**Affiliations:** a Indiana University Medical School, Indianapolis, IN, USA; b Johns Hopkins Bloomberg School of Public Health, Baltimore, MD, USA; c Harvard University, Boston, MA, USA; d New York Medical College, Valhalla, NY, USA; e Neurotrope, Inc., New York, NY, USA

**Keywords:** Bryostatin, memantine, neurorestorative, PKC (Protein Kinase C), severe Alzheimer’s disease, severe impairment battery, synaptic growth factors, synaptogenesis

## Abstract

**Background:**

Bryostatin-activated PKC epsilon pre-clinically induces synaptogenesis, anti-apoptosis, anti-amyloid-β oligomers, and anti-hyperphosphorylated tau.

**Objectives:**

To investigate bryostatin safety, tolerability, and efficacy to improve cognition in advanced Alzheimer’s disease (AD) patients.

**Methods:**

A double-blind, randomized, placebo-controlled Phase II, 12-week trial of i.v. bryostatin for 150 advanced AD patients (55–85) with MMSE-2 of 4–15, randomized 1:1:1 into 20 μg and 40 μg bryostatin, and placebo arms. The Full Analysis Set (FAS) and the Completer Analysis Set (CAS) were pre-specified alternative assessments (1-sided, *p* < 0.1 for primary efficacy, and 2-sided, *p* < 0.05 for pre-specified and *post hoc* exploratory analyses).

**Results:**

The safety profile was similar for 20 μg treatment and placebo patients. The 40 μg patients showed safety and drop-out issues, but no efficacy. Primary improvement of Severe Impairment Battery (SIB) scores at 13 weeks was not significant (*p* = 0.134) in the FAS, although in the CAS, the SIB comparison favored 20 μg bryostatin compared to placebo patients (*p* < 0.07). Secondary analyses at weeks 5 and 15 (i.e., 30 days post-final dosing) also favored 20 μg bryostatin compared to placebo patients. A pre-specified ANCOVA for baseline memantine blocking bryostatin and positive *post-hoc* trend analyses were statistically significant (2-sided, *p* < 0.05).

**Conclusion:**

Although the primary endpoint was not significant in the FAS, primary and secondary analyses in the CAS, and pre-specified and *post-hoc* exploratory analyses did favor bryostatin 20 μg compared to the placebo cohort. These promising Phase II results support further trials of 20 μg bryostatin— without memantine— to treat AD.

## INTRODUCTION

### Alzheimer’s disease therapeutics

Therapeutic strategies for Alzheimer’s disease (AD) have focused on immunotherapy and enhancement or blockade of neurotransmitters at synaptic junctions [1–3]. The latter have generated drugs with some symptomatic efficacy offering some welcome relief to AD patients [4, 5]. A major unmet medical need, however, is the relentless progression of AD. This degeneration of synaptic networks and neurons, as measured directly or indirectly at autopsy, have been found to closely correlate with the degree of cognitive deficits [6]. These autopsy-based correlations have been observed in several subsequent studies [7–9].

With a focus on synaptic and neuronal loss in AD, we have developed a therapeutic strategy that has shown a neurorestorative potential, i.e., to restore lost synapses in AD brains in pre-clinical studies [10], as well as the concomitant potential to prevent apoptosis [10–12], reduce amyloid-β (Aβ) oligomers, lower hyperphosphorylated tau [10–12], and reduce oxidative stress [13]. In a number of pre-clinical studies, activators of PKC epsilon, such as the marine macrocyclic lactone, bryostatin, have been shown to increase synaptic numbers via synaptic growth factors such as BDNF, NGF, and IGF [14, 15]. Specific enzymatic pathways were demonstrated in pre-clinical studies to mediate such effects. These included alpha-secretase activation, Aβ degrading enzyme activation, m-RNA stabilization of growth factor mRNAs, and inhibition of GSK-3β-mediated tau phosphorylation [16].

### PKC epsilon mechanism(s) of action [11, 16, 17]

**Fig.1:**
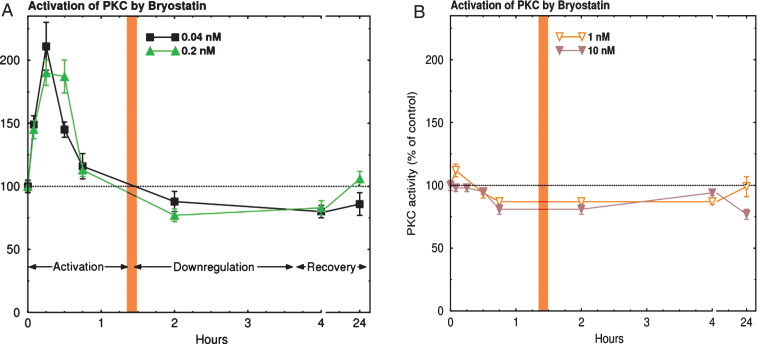
PKC Activation Time course in cultured human neuroblastoma SH-SY5Y cells. PKC activation was measured by the degree of histone phosphorylation in response to continuous application of Bryostatin. Note that in (A), activation for less than 40 minutes occurs with doses below 1 nM (0.01–0.4 nM). Activation is followed by a down-regulation phase (inhibition) for several hours. In (B), with doses of 1 nM or more, activation markedly decreases, but the downregulation phase remains [11]. nM, nanomoles.

Previous studies with purified enzyme activity, cultured neuronal enzyme activity, and *in vivo* animal endogenous enzyme activity have shown (see Fig. 1A, B) that there are characteristically three phases of PKC activity: 1) activation (15–30 min), 2) downregulation or inhibition (>2 h), and 3) *de novo* synthesis (>2 days). These phases have been explained by known enzymatic pathways that begin with translocation of the cytoplasmic enzyme to the cellular membrane (in response to second messengers such as diacylglycerol, calcium, arachidonic acid, and phosphatidylserine), activation in association with the cellular membrane, ubiquitination and the onset of proteasome degradation, and recycling of degradation products into *de novo* synthesis of the original enzyme. Because of these phases, dose-dependence is typically an inverted-U shaped curve that has activation in the lower doses (0.02–1.0 nM), plateau constancy at dosing maximum (>1.0 nM) and inactivation on the descending limb of the dose-dependent curve. This is illustrated in Fig. 1 for activation of PKC epsilon, at lower doses of bryostatin, and such that at higher doses (in this study most likely corresponding to the 40 μg cohort), bryostatin predominantly causes downregulation. These higher doses would be expected to be ineffective for increasing target activity. Therefore, these higher doses could also be ineffective for patient benefits that derive from PKC epsilon activation. The 40 μg cohort in the clinical trial described here can be interpreted to correspond to the higher doses observed *in vitro* (Fig. 1A, B) that cause downregulation (inhibition) of PKC. Furthermore, clinically, this higher dose protocol (the 40 μg cohort) showed reduced safety, increased drop-out rate, and no efficacy signals (See Results, below).

### Phase I experience with bryostatin

Extensive experience with bryostatin used as an anti-tumorigenic agent with >1400 patients indicated that the drug could be well-tolerated— anticipated particularly in the lower dose range <30 μg/m^2^/week. At lower doses, bryostatin causes significant activation of PKC epsilon, while in higher doses downregulation or inhibition predominates. A pharmacokinetic study with AD patients demonstrated a peak activation of PKC within 1 h of infusion onset, closely associated with a measured rise to peak of bryostatin blood levels [18]. Furthermore, compassionate use trials showed promising improvements in AD patients with advanced disease [18]. These results suggested potential efficacy of bryostatin in advanced AD patients toward whom the present trial was oriented. It was this experience with the compassionate use trial patients as well as with numerous pre-clinical studies that motivated the design of the clinical trial reported here. The present design, however, does not preclude future clinical testing in earlier stage AD patients.

## METHODS

### Study design and patients

As a first-in-Alzheimer’s-patients, multiple dose trial, safety and tolerability were the primary objectives, and bryostatin’s efficacy for cognitive improvement was the secondary objective. The Full Analysis Set (FAS, mITT, modified intent to treat) as well as the Completer Analysis Set (CAS) were each included in the pre-specified Statistical Analysis Plan (SAP) as alternative population sets to assess the primary, secondary, and exploratory efficacy endpoints. The mITT included trial participants who dropped out (Fig. 3) prior to the 13-week time point. Bryostatin efficacy was considered more directly related, however, to Severe Impairment Battery (SIB) scores of patients who in fact received the entire planned drug dose regimen. The primary endpoint was quantified at 13 weeks for patients in the mITT group, and as an alternate population, for patients who received the full dosing schedule of 12 weeks, and had a completed SIB score measure at the week 13 time point (CAS). Similarly, secondary endpoints at 5, 9, and 15 weeks were evaluated in patients who received drug at those time points. The SIB was the primary metric of cognitive performance, while a secondary metric, the Alzheimer’s Disease Cooperative Study – Activities of Daily Living - Severe Impairment Version (ADCS-ADL-SIV), provided additional data on functional benefit.

**Fig.2:**
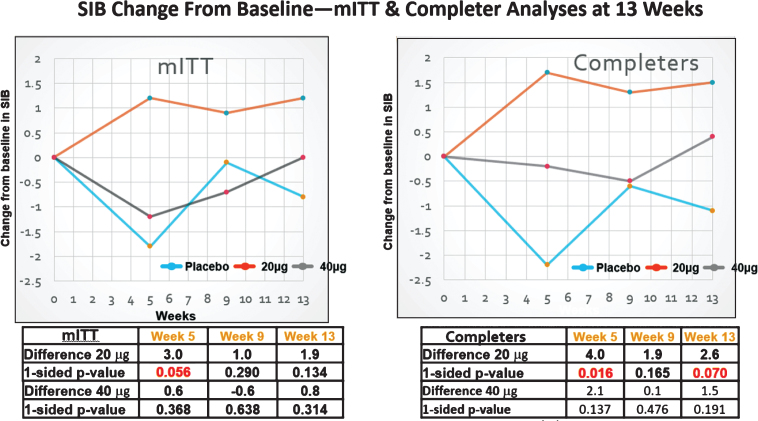
SIB changes in the MITT (FAS) and completers sets. Clear improvement signals in the SIB were only observed with the 20 μg dosing protocol.

**Fig.3:**
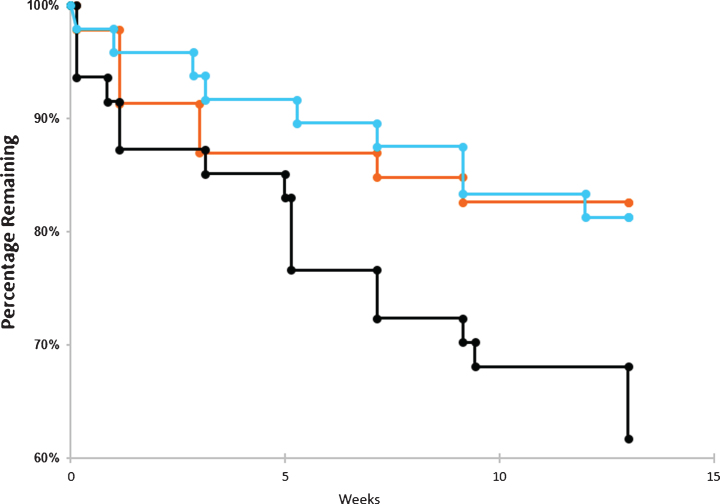
Drop outs by time and dose. Dropout rate over the course of the trials for placebo arm (blue), 20 μg arm (orange), and 40 μg arm (black). The number of patients withdrawing from the trial were tabulated for each cohort in the graphs above. No survival analyses were conducted.

### Design details

We conducted a double-blind, randomized, placebo-controlled Phase II trial, in which bryostatin was administered by intravenous infusion (45±5 min, with a total of 7 doses) to patients with advanced AD over the course of 12 weeks. The study was approved by Copernicus central IRB and by the applicable local IRB where required. Patients and their primary caregivers gave written consent prior to participation. For this study, the IRB required a signature by the patient’s legally authorized representative, who may or may not have been the primary caregiver. A copy of the protocol, NRP101-202, allowed to proceed by FDA, is available (Protocol ID: NTRP 101 - 202).

Adults aged 55–85 with cognitive deficits present for at least 2 years, Mini-Mental State Examination, version 2 (MMSE-2) score of 4–15 inclusive, and a diagnosis of AD were considered eligible for this trial. The MMSE or Folstein test is a 30-point questionnaire that is used extensively in clinical and research settings to measure cognitive impairment [19]. Low scores indicate greater impairment. Version 2 of the MMSE was published in 2010, expanding the original’s usefulness in populations with milder forms of cognitive impairment. After consent and confirmation of eligibility, study participants were randomized 1:1:1 into one of three treatment arms: 20 μg bryostatin, 40 μg bryostatin, or placebo. For the two bryostatin treatment arms, two loading doses (20% higher; 24 μg and 48 μg, respectively) were followed by infusions of 20 μg bryostatin or 40 μg bryostatin administered every two weeks for the remaining 5 doses. To preserve the blind, patients assigned to the placebo treatment arm were randomized to receive a “loading dose” volume of placebo identical to the volumes of the 24 μg and 48 μg treatments for the first two infusions, followed by administration of placebo volumes identical to the 20 μg and 40 μg dose administrations at subsequent dosing visits. A total of 7 doses were administered to each patient who completed the 12-week treatment period. The dosing schema is below.


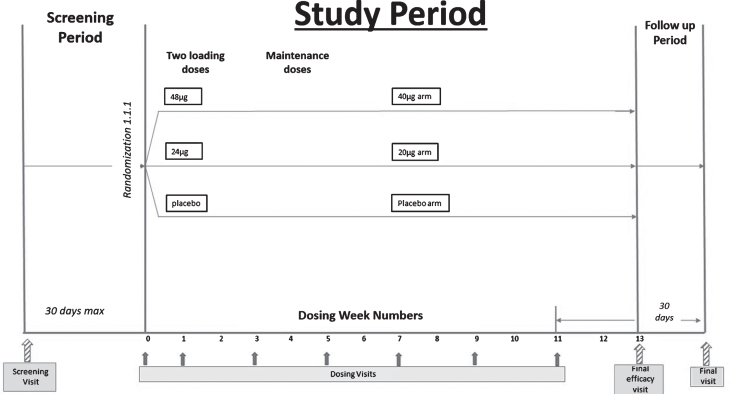


A total of 264 patients was screened at 27 clinical sites in the United States. Among the screened patients, a total of 147 was randomized and treated with at least one dose of study drug. These patients were included in the Safety Analysis Set (SAS). A total of 135 subjects provided a post-baseline efficacy assessment and were analyzed as the Full Analysis Set (FAS) based on the mITT principle, defined as all randomized subjects who received at least one dose of their assigned study drug, and who had at least one post-baseline efficacy assessment. A total of 113 subjects out of 141 treated (80.1%) performed a week 13 evaluation of the SIB and was analyzed as the CAS. Thirty-five subjects in the SAS, and 29 subjects in the FAS withdrew early from the trial. The most common reason for early study termination was withdrawal of informed consent (18 subjects), followed by 11 subjects who left the study early due to an adverse event (AE).

### Selection of doses

As an exploratory trial, three separate doses were identified to define a range of dosing efficacy: zero (placebo), 20 μg, and 40 μg. The 40 μg dose corresponded approximately to the 25 μg/m^2^ doses that were used in Compassionate Use protocols [18]. These doses were derived from three sources of extensive prior data collection: pre-clinical *in vitro* studies with isolated PKC enzymes and their substrates, pre-clinical *in vivo* studies with AD transgenic mice, and compassionate use patients. These studies and the relevant data are all described in several publications that are referenced in this article.

The range of 0 to 40 μg was not arbitrary, but instead was directly derived from empirical experience. In the Phase II A study [18], Compassionate Use protocols used doses that approximated the 40 μg (25 μg/m^2^) with frequencies that apparently were too high, thus causing downregulation after 3 consecutive weekly doses. Inference of downregulation was based on direct measurement of PKC epsilon in a patient’s blood samples. For this compassionate use patient, it was possible to measure blood PKC epsilon levels [18] rise in close association with the patient’s initial improvement with weekly dosing, followed by a decline in this improvement as the PKC epsilon levels fell, in fact, showing downregulation (inhibition). These and other compassionate use results guided our selection of alternate weekly doses— to avoid this observed downregulation— in moderately severe to severe AD patients. These compassionate use results also suggested that too frequent bryostatin over time would produce downregulation and not the activation of PKC epsilon that our pre-clinical studies indicated was associated with cognitive, synaptogenic, and anti-amyloid benefit. On that basis, therefore, we anticipated that the 40 μg dose might be too high a dose and weekly might be too high a frequency (e.g., weekly). Given those possibilities, we believed that we should test an intermediate dose of 20 μg, also administered at a lower frequency than that of the compassionate use protocols. A similar sequence of PKC activation followed by prolonged downregulation had been previously demonstrated in a clinical oncology trial [20] with a dose level and frequency of bryostatin administration comparable to that of our Compassionate Use protocols for AD.

The biochemical data presented in Fig. 1 A, B illustrates *in vitro* PKC epsilon activation that follows an inverted U-shaped dose-response curve. Such an inverted U-shaped dose-response curve was, in fact, suggested by the improvement signals in the SIB scores that were observed in the present trial.

### Drop-outs

Figure 3 illustrates that patients in the Placebo Group and the 20 μg cohort had comparably modest drop-outs (placebo = 12.5%, 20 μg = 17.4%). By contrast, the 40 μg Cohort at the specified protocol dosing frequency, found to be ineffective (See Methods and Results, below) and to have many more associated side-effects, had a markedly increased number of drop-outs (38.8%). For most of the data analyzed below, therefore, this 40 μg cohort, at the protocol frequency reported here, was considered a limiting dose that would not be useful clinically. However, the 40 μg lack of efficacy was consistent with the known U-shaped curve of dose-response previously described in the *in vitro* biochemical literature (see Fig. 1) and thus provided a dosing limit for lower, potentially therapeutic doses, also observed here (see below).

### Randomization and masking

The contract research organization, Worldwide Clinical Trials (https://www.worldwide.com) was responsible for the allocation of patients into treatment arms, and for collecting and masking patient data throughout the trial. Once all eligibility criteria for the study had been met, the subjects were randomized by the statistical group at Worldwide Clinical Trials using an Interactive Web Response System (IwRS). Patients were stratified by baseline MMSE-2 scores, dichotomized into low (4–9) and high (10–15) scores.

After a randomization number was assigned to patients using the IwRS, a twelve-week supply of study drug for that randomization number was shipped to the site. Randomization and scheduling of the first study drug infusion were timed to allow for receipt of the study drug prior to the scheduled study treatment. Drug kits, each containing 7 vials of bryostatin for infusion or placebo for infusion, lyophilized presentations and 7 vials of PET diluent for reconstitution were shipped to the unmasked individual at each site who was responsible for kit storage and drug preparation for infusion. No other study staff member handled the study drug kits. The shipped kits were identified by kit number and their contents did not disclose the identity of the study drug containing either bryostatin or placebo.

The Sponsor and investigators were blinded to treatment assignment throughout the trial, while statisticians performing the data analyses for DSMB safety reviews conducted during the study, were partially masked (e.g., they knew only treatment assignments as ‘A’, ‘B’, or ‘C’) when performing the analyses of the final data. The DSMB had the option of requesting to see completely unmasked data if there were any safety concerns.

### Outcomes

The primary safety outcome was treatment emergent adverse events (TEAEs). AEs were defined as expected or unexpected events that lead to discomfort or unfavorable symptoms on the part of the patient. One Adverse Event of Special Interest (AESI) was myalgia, which was reported as dose-limiting in oncology trials and appeared to be dose dependent and cumulative across all the oncology studies, sponsored by the NCI. Serious Adverse Events (SAEs) were defined as any untoward medical occurrence that was fatal, life-threatening, required in-subject hospitalization or prolonged existing hospitalization, resulted in persistent or significant disability or incapacity, was a congenital anomaly or birth detect, or was an important medical event. SAEs and AEs may not necessarily be causally related to treatment. Secondary safety endpoints included vital signs obtained from physical examination, 12-lead electrocardiogram (ECG) results, scores on the Columbia Suicide Severity Rating Scale (C-SSRS), and assessment of hematology and blood chemistry. In this study, safety data were analyzed descriptively in all subjects who received any dose of study drug (including partial infusions).

Efficacy assessments used in this trial included the SIB [21], the ADCS-ADL-SIV [22], the Clinical Global Impression of Improvement (CGI-I) [23], and Neuropsychiatric Inventory (NPI) [24]. The SIB is used to assess cognition in subjects with moderate and severe AD. It is divided into nine subscales that include attention, language, orientation, memory, praxis, visuospatial ability, construction, social skills, orienting head to name. Forty questions are included with a point score range of 0–100. Lower scores indicate greater cognitive impairment.

The ADCS-ADL-SIV is a 19-item functional assessment of the performance of activities of daily living for subjects with moderate to severe AD. Each item is rated from the highest level of independent performance to complete loss. Total score ranges from 0–54 with lower scores indicating greater functional impairment.

The CGI-I is used to assess global change in the subject’s condition compared to baseline before treatment. This is a seven-point scale ranging from (1) very much improved to (7) very much worse.

The NPI is a caregiver interview-based rating scale assessing 12 behavioral disturbances occurring in dementia subjects. Items are scored for both frequency and severity. Total scores range from 0–144 with higher scores indicating greater behavioral disturbances. For each item, the associated caregiver distress is also assessed.

The primary statistical objective for efficacy was to estimate the effect of bryostatin on the mean change in the SIB after 12 weeks of treatment (week 13). Secondary SIB assessments were taken at 5, 9, and 15-weeks post-first dose. Changes in the ADCS-ADL-SIV, CGI-I, and NPI were assessed as secondary endpoints.

The primary and secondary efficacy endpoints were defined as the change in the SIB score at the assessment time points from baseline. The primary SIB endpoint was the change at 13-weeks post-dose from baseline, while secondary SIB assessments were taken at 5, 9, and 15-weeks post-dose. Additional efficacy endpoints (secondary) included changes in the ADCS-ADL-SIV, CGI-I, and NPI metrics.

Pre-specified exploratory analyses were performed on patients in the 20 μg bryostatin and placebo arms not taking memantine (e.g., called here “memantine free”) as concomitant standard of care (SOC; the baseline treatment already followed for patients enrolled) baseline therapy for the duration of the trial. In these exploratory analyses, the primary endpoint was modified slightly from the pre-specified endpoint described in the study statistical analysis plan. For patients studied in these pre-specified exploratory analyses, we defined the primary efficacy endpoint as the change in the average SIB score obtained at both the week 13 and week 15 from baseline. If a patient had missing SIB data at either week 13 or week 15, then the average 13/15-week SIB was given by the one obtained SIB. Finally, the secondary efficacy endpoints for the memantine free patients included the SIB change at week 5 from baseline, and at week 9 from baseline. Another pre-specified exploratory analysis, an ANCOVA analysis using a 2-tailed, *p* < 0.05 criterion, was planned to test for memantine interaction with bryostatin. For the ANCOVA, the FAS was used. Other pre-specified exploratory analyses showed no effects of SOC donepezil administration on the bryostatin treatment effects.

### Statistical analysis

As the first multiple bryostatin dose protocol in AD patients, power analyses for this exploratory study were based on a bryostatin treatment effect on the mean change in the SIB at week 13 from baseline. Our power analyses determined that 150 subjects equally randomized among the three treatment arms would provide at least 80% power, with a less demanding one-sided alpha = 0.1, to detect signals for a treatment effect favoring bryostatin in the comparison of 1) either bryostatin dosing arm versus placebo, and 2) the pooled bryostatin arms versus placebo. Reaching this level of a treatment effect, however, was not interpreted here as demonstrating statistical significance. These power analyses were based on a minimum mean SIB (SD) change at week 13 from baseline≥6.5 (14) points in either bryostatin treatment arm as compared to placebo. This power estimate allowed for a lost-to-follow-up rate of 15% during the trial, approximated by the drop-outs in both the placebo and the 20 μg cohorts.

Initially, exploratory analyses were performed to evaluate the impact of baseline covariates on the efficacy variables. The primary endpoint of the change in SIB at 13 weeks from baseline was analyzed using the Mixed Model for Repeated Measures (MMRM). The MMRM regression model included random patient effects and fixed effects for treatment (three treatment arms), baseline MMSE-2 stratum, baseline SIB, scheduled visit (treated as a categorical or ‘factor’ variable), and scheduled visit by treatment interaction terms. The results were evaluated at a one-side alpha level of 0.10, as specified in the statistical analysis plan submitted to the FDA for both the FAS and CAS groups. Least-square means (LSM) and 2-sided 80% confidence intervals (CI) were provided for treatment group differences by each follow-up visit. The change from baseline at every visit (indirectly follow-up time) was the outcome of interest and there were multiple follow-up times. The LSM contrasts obtained from this MMRM allowed for an estimate of the treatment effect at the primary time point of week 13, as well as at the secondary time points of week 5, 9, and 15. Given the relatively small sample size for this trial in conjunction with the relatively large number of the parameters required by this MMRM, this model, in retrospect, may not have been most appropriate for the primary data analysis. However, without knowledge of the unblinded data and relevant sub-populations, this model was thought to be most appropriate at the time that the trial and its SAP were pre-specified. Results from this pre-specified model are, therefore, reported here.

Secondary endpoints for ADCS-ADL-SIV, MMSE-2 (excludes MMSE Stratum variable), and NPI at week 13 were analyzed using a statistical model that was similar to the one used for analysis of the SIB. Finally, the CGI-I secondary endpoint was analyzed in similar fashion, except the model did not have a baseline value as a covariate. There were no adjustments in *p*-values for multiplicity.

While the initial, pre-specified, primary efficacy analysis used MMRM, this complex model, requiring multiple parameters was considered not to be necessary to estimate treatment effects in the pre-specified exploratory analyses. This is especially true given that one purpose of MMRM is to adequately handle data missing at random, which was not an issue in the exploratory analyses as we had very few missing SIB observations for patients off memantine. Moreover, to avoid large potential intra-patient SIB variation over time, for the exploratory analysis, we considered the change in average score collected during week 13–15 from baseline as the endpoint. The statistical assessment of treatment effect estimate was based on the simple, transparent two-sample t-statistic.

Exploratory analyses were pre-specified in the SAP to examine the potential interaction of SOC baseline therapy— either donepezil and/or memantine. As discussed below (see Discussion), memantine was a particularly important interaction analysis because of the known biochemical regulation by PKC of memantine’s target, the NMDA receptor (see Discussion). Because the data from pre-specified exploratory analyses initially showed no efficacy for patients on baseline memantine therapy, further exploratory analyses here focused on the off-memantine patients in the low dose bryostatin (20 μg) and placebo arms. These analyses occurred in three stages. In the first stage, the treatment-specific SIB means of the primary efficacy endpoint were calculated, and group differences statistically assessed by the *t*-test for two independent samples, assuming unequal variance. In addition, the Wilcoxon Rank Sum test was performed to determine the robustness of the *t*-test results. Second, a one-sided multivariate Rank Sum test was used that simultaneously considered SIB differences at week 5, week 9, and the average of week 13/15 from baseline [25]. The *post-hoc t*-test was done on the change in the 13/15 SIB measure minus baseline SIB for the memantine-free patients (one delta measurement per person NOT the MMRM). Finally, a trend analysis was performed on the repeated SIB measures over time. MMRM analysis that included the fixed effects of treatment and a treatment-by-time interaction, with time treated as a continuous variable, was used to estimate summary measures of treatment-specific SIB outcomes changing over time as linear regression slopes. This approach was in contrast to the more complicated MMRM used in the pre-specified primary analysis described above, which estimates discrete treatment contrasts at each of the several follow-up time points. No endpoints were imputed in the analyses of off-memantine patients due to the low loss to follow-up rate for these patients.

As indicated above, the Statistical Analysis Plan (SAP) states that *p*-values are to be reported as 1-sided for the pre-specified primary efficacy analysis. Although his level for type 1 error has been used in other Phase II trials [26], it will not be interpreted here as indicative of statistical significance.

Missing outcomes were not imputed in the pre-specified primary, secondary, or exploratory analyses. The MMRM analysis provides robust estimates of the treatment effect if these data are missing at random. All statistical analyses were performed using SAS, STATA version 14.0, and R software packages.

## RESULTS

As shown in Table 1, demographics and baseline patient characteristics were very similar across all three treatment arms. Mean (SD) patient ages ranged from low of 70.2 (7.5) in the 40 μg arm to high of 73.5 (7.7) in the placebo arm. Overall, patients were predominately white (>90%) and non-Hispanic (>89%). Study participants were approximately equally distributed between males and females in all three treatment arms. Placebo patients and patients in the 40 μg dosing arm had a median MMSE-2 at baseline of 10.0, while those in the 20 μg arm had a median baseline MMSE-2 of 11.0. Mean (SD) time from AD diagnosis to screening was longest for placebos at 5.6 (2.9) years, and shortest in the 20 μg arm, with a mean (SD) AD duration = 4.6 (3.0). See Table 1.

**Table 1:** Demographic, baseline, and safety event variables for the safety analysis set patients

	Placebo (N = 48)	Bryostatin 20 μg (N = 46)	Bryostatin 40 μg (N = 47)
*Demographics*
Age (y)
Mean (SD)	73.5 (7.7)	71.2 (8.4)	70.2 (7.5)
Sex
Female	23 (47.9%)	26 (56.5%)	22 (46.8%)
Male	25 (52.1%)	20 (43.5%)	25 (53.2%)
Race
White	45 (93.8%)	42 (91.3%)	46 (97.9%)
African American	3 (6.3%)	3 (6.5%)	1 (2.1%)
Asian	0 (0%)	1 (2.2%)	0 (0%)
Ethnicity
Hispanic (%)	5 (10.4%)	4 (8.7%)	3 (6.4%)
Not Hispanic (%)	43 (89.6%)	42 (91.3%)	44 (93.6%)
BMI (kg / m^2^)
Mean (SD)	25.9 (3.8)	25.9 (4.1)	26.8 (4.6)
BSA (m^2^)
Mean (SD)	1.8 (0.2)	1.8 (0.2)	1.9 (0.2)
*Baseline*
MMSE-2
Mean (SD)	10.0 (3.5)	10.5 (3.2)	10.0 (3.5)
Median	10.0	11.0	10.0
Rosen-Modified Hachinski Score
Mean (SD)	0.8 (0.7)	0.5 (0.8)	0.6 (0.6)
Median	1.0	0.0	1.0
AD Diagnosis at Screen (y)
Mean (SD)	5.6 (2.9)	4.6 (3.0)	5.2 (2.3)
*Safety Variables*
Any TEAE	28 (58.3%)	30 (65.2%)	39 (83.0%)
Treatment-related TEAE	8 (16.7%)	17 (37.0%)	24 (51.1%)
Serious TEAE	3 (6.3%)	1 (2.2%)	6 (12.8%)
Myalgia	0 (0%)	0 (0%)	4 (8.5%)
Fatal TEAE	0 (0%)	0 (0%)	1 (2.1%)

### Safety

Overall, patients in the 20 μg treatment arm demonstrated minimal differences from the placebo patients in safety assessments (see Table 1). Both groups had similar numbers of TEAEs (28 events in the placebo group versus 30 events in the 20 μg group). In contrast, patients in the 40 μg treatment arm, observed below (as expected) to have no efficacy, had significantly greater TEAEs (57 events) than patients in either of the other treatment arms. The TEAEs observed more often in the 20 μg treatment group versus the placebo group were infusion site reactions (eight events versus three events in placebo) and diarrhea (five events versus one event in the placebo group). It is important to emphasize that once the appropriate precautions were taken including WebEx-based training on IV infusion, aseptic techniques and universal precautions, no additional infusion site reactions occurred (in the 2nd half of the trial), suggesting that this AE can be prevented.

Other common TEAEs included headache, fatigue, and myalgia. Myalgia was seen in five subjects; four of whom were given the 40 μg dose. Observed myalgia was mostly mild and managed with analgesics. There were more TEAEs of diarrhea, headache, and fatigue among patients in the 40 μg arm as compared to patients on the other two treatments. Patients in both bryostatin groups reported higher rates of infusion site TEAEs than the placebo group. Again, with the appropriate precautions in the 2nd half of the trial, no infusion site reactions occurred.

There was one death in the trial, a subject in the 40 μg arm who suffered a severe TEAE of worsening of AD that was unrelated to bryostatin treatment. In addition, 12 (8.5%) subjects had 14 treatment emergent non-fatal SAEs; four subjects with four events in the placebo arm, two subjects with two events in the 20 μg treatment arm, and six subjects with eight SAEs in the 40 μg treatment arm. No apparent differences were seen between treatment groups for most vital signs and ECG. However, there was a decline in weight among patients in both bryostatin arms, a result more prominent in the 40 μg dose group as compared to the 20 μg dose group (i.e., mean (SD) weight loss = –1.65 (2.77) kg in the 20 μg arm versus a mean (SD) weight loss = –2.98 (2.10) kg in the 40 μg arm). In contrast, there was a slight weight gain among the placebo group (mean (SD) weight gain = 0.44 (2.52) kg). Furthermore, five subjects in the 40 μg treatment arm had five TEAEs of weight decrease, three of which were judged to be related to bryostatin. No weight-related TEAEs were observed in the 20 μg arm. There were no differences between treatment groups on the C-SSRS results, as most subjects did not have suicidal thoughts. There were no attempts at suicide by any patient during the trial. Finally, there were no apparent differences between treatment arms in laboratory assessments.

### Efficacy

#### Primary analyses

Among the FAS patients (some of whom did not receive the full dosing regimen, i.e., including drop-outs that had at least one post-dose SIB measure), no evidence of improvement signals between the 20 μg or the 40 μg arm and the placebo arm was seen at the 13-week primary endpoint (see Fig. 2A and Table 3). By week 13, those in the 20 μg arm signals were observed to show an increase in mean (SEM) SIB of 1.16 (1.15) from baseline, while the placebo mean (SEM) SIB decreased by –0.79 (1.33) points from baseline during this same time period (difference [80% CI] = 1.94 [–0.31, 4.19], *p* = 0.134). At the 5- week secondary endpoint, comparison of the bryostatin 20 μg cohort versus the placebo cohort favored bryostatin (difference [80% CI] = 2.96 [0.58, 5.34], *p* = 0.056).

Among patients exposed to the complete dosing regimen and who had a 13-week SIB assessment (CAS patient group), evidence of benefit in the SIB scores favored the 20 μg bryostatin arm versus the placebo arm for the primary SIB endpoint at weeks 5 and 15 (Fig. 2B; Table 2). Baseline SIB scores were similar across all three treatment arms.

**Table 2:** Results of the MMRM analysis for the completer analysis set

	Week 5	Week 9	Week 13
Diff. 20 μg versus Placebo (80% CI)	4.0 (1.6, 6.4)	1.9 (–0.6, 4.3)	2.6 (0.4, 4.9)
One-sided *p*-value	0.016	0.165	0.070
Diff. 40 μg versus Placebo (80% CI)	2.1 (–0.4, 4.6)	0.1 (–2.3, 2.5)	1.5 (–0.7, 3.8)
One-sided *p*-value	0.137	0.476	0.191

**Table 3:** Results of the MMRM analysis for the full analysis set

	Week 5	Week 9	Week 13
Diff. 20 μg versus Placebo (80% CI)	3.0 (0.6, 5.3)	1.0 (–1.4, 3.4)	1.9 (–0.3, 3.4)
One-sided *p*-value	0.056	0.290	0.134
Diff. 20 μg versus Placebo (80% CI)	0.6 (–1.7, 3.0)	–0.6 (–2.9, 1.7)	0.8 (–1.4, 3.0)
One-sided *p*-value	0.368	0.638	0.314

At week 13, the mean (SEM) SIB increased by 1.51 (1.12) points from baseline in the 20 μg arm, while placebo patients showed a decrease in their mean (SEM) SIB scores from baseline of –1.12 (1.39) (difference [80% CI] = 2.63 [0.35, 4.91], *p* = 0.070).

At week 5, there was also evidence of benefit in mean SIB scores from baseline among 20 μg bryostatin patients versus the placebo patients (difference [80% CI] = 4.00 [1.63, 6.38], *p* = 0.016). See Table 2. No differences in mean SIB changes from baseline in the CAS patient sub-group were seen in the 40 μg treatment arm versus the placebo arm at any follow-up time point (see Fig. 2B). Similarly, pooling the bryostatin-treated patients across both dosing arms did not produce statistically significant differences in mean SIB changes compared to the placebos at any follow-up time points.

As seen in Fig. 3, the dropout rate was very similar between the 20 μg arm and the placebo arm throughout the course of the trial. In contrast, the dropout rate was very high for the 40 μg arm as compared to the other two arms. The high dropout rate among those exposed to the highest dose of bryostatin is most likely the result of more side effects and AEs experienced by patients in this arm and is consistent with a lack of PKC activation efficacy at this dosing level.

**Fig.4:**
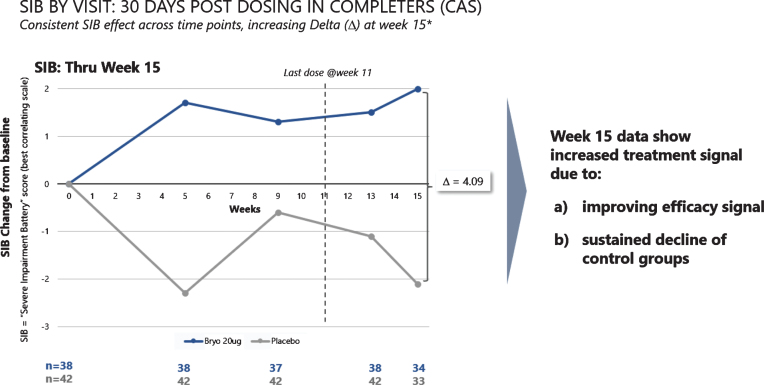
Improvement scores in SIB scores – through week 15. SIB improves throughout the trial, with a decline in SIB for the placebo patients (lower curve).

When we assessed post dosing outcomes on the SIB, we found that patients in the 20 μg arm showed benefits from baseline at week 15 in both the CAS and FAS patient groups (See Fig. 4). Among patients who were exposed to the complete dosing regimen (Completers), the 15-week mean (SEM) SIB increased from baseline by 1.96 (1.23) points in the 20 μg treatment group, while the placebo group showed a decline in mean (SEM) SIB = –2.13 (1.76), giving a treatment difference of greater than 4.0 points (difference (80% CI) = 4.09 (1.33, 6.85), *p* = 0.029). Similarly, including patients who dropped out prior to receiving all 7 doses (i.e., the FAS), the 15-week mean (SEM) SIB increased from baseline by 1.77 (1.34) points in the 20 μg arm, while the placebo group showed a decline in mean (SEM) SIB = –1.82 (1.73), giving a treatment difference favoring bryostatin (80% CI) = 3.59 (0.79, 6.39), (*p* = 0.050).

Among secondary outcome measures, we found that the ADCS-ADL-SIV mean score favored bryostatin 20 μg versus placebo at week 13 (*p* = 0.082) in the CAS patient subset. In contrast, there were no benefits of bryostatin 20 μg versus placebo in the ADCS-ADL-SIV mean score at week 13 from baseline in the FAS (*p* = 0.104).

Much smaller effects were seen between these two arms in the ADCS-ADL-SIV scores at week 5 and week 9. The NPI changes from baseline did not show convincing improvement in both bryostatin dosing arms versus the placebo patients.

### Exploratory analyses

Because PKC isozymes have been shown in previous pre-clinical studies to regulate the NMDA receptor (see Discussion below), the clinical effects of a known blocker of the NMDA receptor, memantine, used as a baseline SOC during the bryostatin protocols, were considered particularly important among the pre-specified exploratory parameters. As mentioned above (see Statistics), except for the multivariate Rank Sum test, all *p*-values for the exploratory analyses are reported as 2-sided, and an alpha level of 0.05 was considered statistically significant.

**Fig.5:**
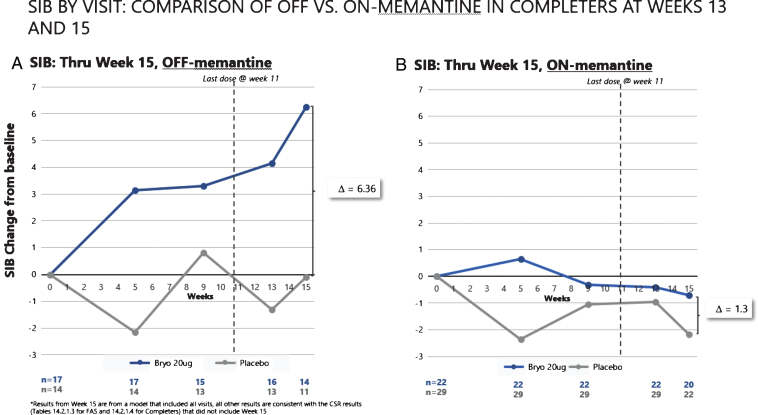
SIB improvement signals (A) are clear with repeated doses of bryostatin in the absence of memantine. No such improvement was apparent with SOC memantine (B).

As can be seen in Fig. 5A, the patients who received no concomitant memantine baseline therapy in the 20 μg bryostatin arm showed evidence of a sustained SIB improvement from baseline over the course of the trial. In contrast, the patients who did receive baseline memantine (Fig. 5B) showed no evidence of SIB improvement over time. Among the patients not receiving memantine, the mean SIB change at the average of week 13 and week 15 time points from baseline was significantly greater in the 20 μg bryostatin arm as compared to placebo patients (difference (95% CI) = 6.1 (1.5, 10.7) points; *p* = 0.012). This improvement of SIB scores persisted after controlling for baseline SIB and MMSE-2 strata at randomization in Analysis of Covariance models. For comparison purposes, we also considered the change in SIB at week 13 from baseline, the original primary endpoint, among patients who received no concomitant baseline memantine therapy. Results of this analysis were shown to produce significant improvement in the mean SIB change from baseline in the 20 μg versus the placebo treatment arm (difference (95% CI) = 5.6 (0.4, 10.9) points; *p* = 0.035).

In the analysis using the method of Wei and Lachin [27] that simultaneously tested for the treatment differences in SIB at week 5, week 9, and week 13 from baseline, we found results that were consistent with those of the univariate analyses shown above (see Table 4). These sustained positive results over time provide evidence on the superiority of the treatment over placebo. A pre-specified ANCOVA analysis for the interaction of memantine with the 20 μg bryostatin cohort also showed significant bryostatin benefit (*p* < 0.024), 2-tailed, *p* < 0.05.

**Table 4:** Pre-specified exploratory analytical results among memantine free patients

SIB Change from Baseline	Placebo	20 μg Bryostatin	*T*-test
Delta Mean (SD)	Delta Mean (SD)	t-statistic (p val)
Week 5	–1.20 (10.26)	3.44 (5.75)	1.56 (0.134)
Week 9	0.79 (7.44)	3.47 (7.04)	1.00 (0.329)
Week 13	–1.14 (6.89)	4.50 (7.01)	2.22 (0.035)
Week 13/15	–0.68 (6.71)	5.41 (5.43)	2.71 (0.012)
Wei-Lachin test^17^	*T*-test	Wilcoxon Test
Weight	Z (1-sided *p*-value)	Z (1-sided *p*-value)
Equal	1.63 (0.052)	1.77 (0.039)
Variance^-1^	1.55 (0.060)	1.75 (0.040)
Optimal	1.10 (0.135)	1.63 (0.052)
Trend Analysis
	Random Intercept Model	Random Intercept, Slope Model
Slope (Placebo) (95% CI)	0.019 (–0.19, 0.22)	0.019 (–0.19, 0.22)
Slope (Bryostatin) (95% CI)	0.38^**^ (0.18, 0.56)	0.38^**^ (0.18, 0.57)
Interaction (95% CI)	0.36^*^ (0.08, 0.64)	0.36^*^ (0.08, 0.64)

^*^p < 0.05; ^**^p < 0.001; 2-tailed *t*-test, alpha of 0.05 for significance.

**Fig.6:**
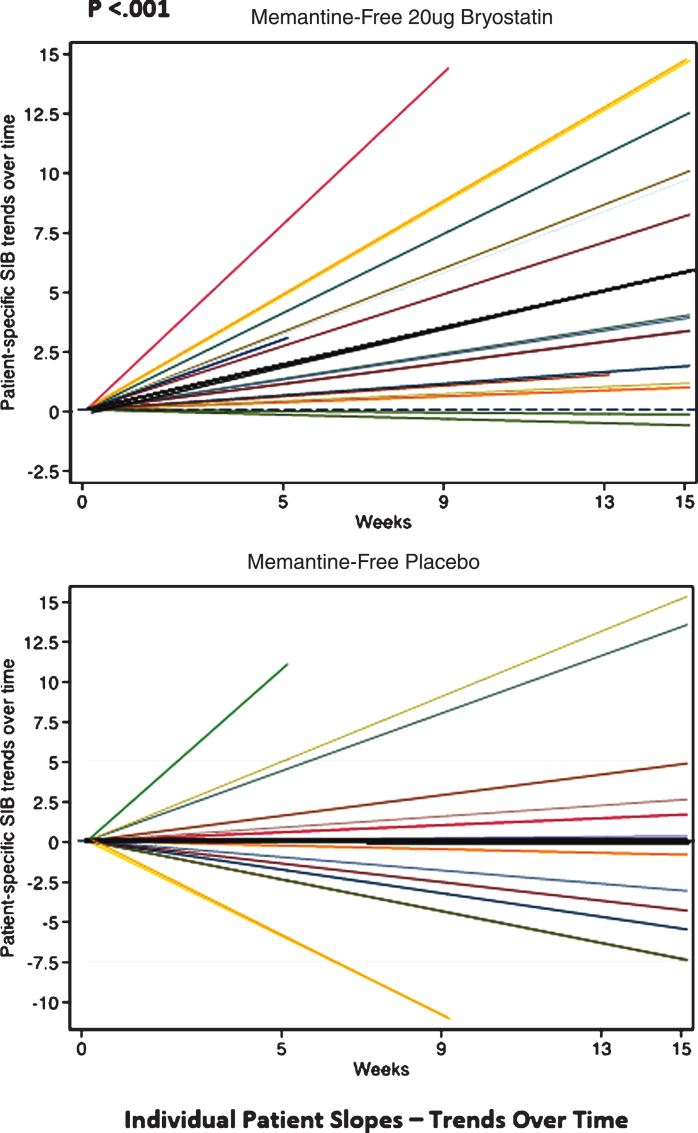
Individual SIB slopes (e.g., trends over time) from baseline (various color lines), and overall treatment SIB slopes (darker black lines) for memantine-free 20 μg bryostatin arm, (Top); and memantine-free, placebo arm, (Bottom), respectively. Based on the statistical analysis, only the 20 μg bryostatin, memantine-free group, overall treatment (dark black line, Top) shows a significant (*p* < 0.001) positive SIB trend (SIB improvement with repeated doses over time) suggesting a treatment effect of bryostatin for this group only. With memantine present, neither the 20 μg bryostatin arm nor the placebo arm showed a significant positive SIB trend.

Finally, in the trend analyses, we found that the SIB values did not increase over time for the placebo patients under the MMRM models, resulting in slopes that were non-significantly different from zero (e.g., ‘zero-slopes’). In contrast, the SIB slopes for the 20 μg bryostatin patients who did not receive baseline memantine were found to be statistically significant, giving a slope (95% CI) = 0.38 (0.18, 0.57) SIB points per week in the random intercept model, and a slope (95% CI) = 0.38 (0.18, 0.59) points per week in the random intercept and slope model. The interaction terms, which indicate a difference in treatment effect by arm, were significant in both mixed effects models (*p* < 0.012, see Table 4). Trends of individual SIB scores over time from baseline of the patients who received 20 μg bryostatin (20 μg bryostatin, memantine-free; and placebo with memantine-free) are illustrated in Fig. 6. The treatment SIB trend was highly statistically different from 0 (*p* < 0.001) for the 20 μg arm (dark black line), while the treatment SIB trend was not statistically different from 0 for the placebo arm. The trends for individual patients are illustrated in Fig. 6, for patients off memantine, both in response to the 20 μg protocol and in response to placebo.

**Fig.7:**
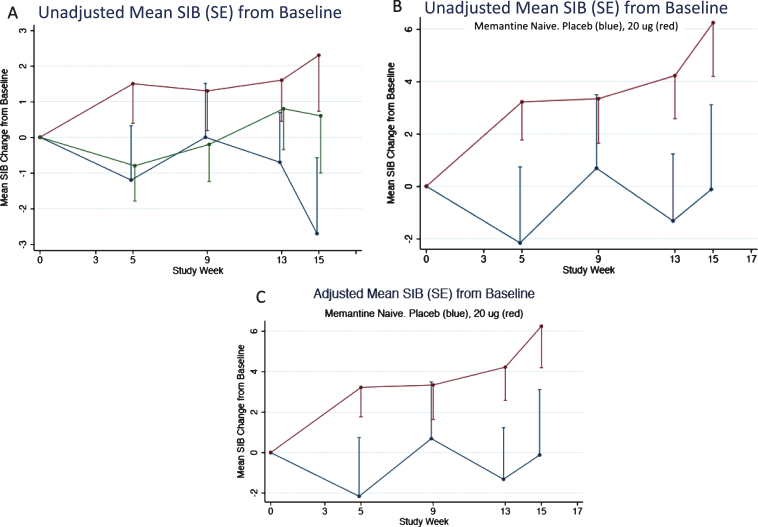
A) Mean SIB changes (unadjusted) from baseline for the FAS subset. 80% confidence intervals are given in Table 3. B) Mean SIB changes (unadjusted) from baseline for the FAS subset for patients not on memantine. C) Mean SIB changes (adjusted) from baseline for the FAS subset for patients not on Memantine. Error Bars = SEM.

As described above, the memantine naïve patients in the 20 μg bryostatin arm showed evidence of sustained benefit of SIB improvement from baseline over the course of the trial. This evidence was also apparent for the patients in the unadjusted FAS (or mITT) and more apparent for the unadjusted memantine free patients (Fig. 7A, B). An adjusted mixed-effects model incorporating time as a factor variable and time by treatment interactions at each time point produced estimates close in value to the unadjusted mean SIB scores from baseline (Fig. 7A-C).

## DISCUSSION

Bryostatin 20 μg did not meet pre-specified primary outcome criteria in the FAS group, but planned analyses showed what we believe are consistent signals of benefit for the drug at this dose in the CAS group. We would emphasize, however, that these improvement signals could be observed in the Completer populations for the primary data analysis but not at a commonly accepted level of statistical significance. However, these SIB improvement signals could be observed at 15 weeks, i.e., four weeks after the termination of the dosing protocol at week 11 for both the FAS and CAS subsets. One exploratory analysis that was pre-specified (the ANCOVA for memantine) and the *post-hoc* exploratory analyses, however, did reach significance at the 2-tailed, *p* < 0.05 level with multiple analytic tests such as a Trend Analysis and Wei-Lachin integrated measurements (Fig. 6A, B). These exploratory analytic results can guide further clinical trials that will use the 20 μg dose on patients who are not on concomitant baseline memantine therapy.

In the present report, the 40 μg produced little or no benefit at the frequency of administration in the selected protocol design. Nor would benefit from this 40 μg dose, at the frequency administered— based on prior pre-clinical and Compassionate Use trial experience— be expected to be effective. However, this higher dose did provide a dose-limit for future trials as well as context for a lower dose, 20 μg, at this frequency. Because this was a first-in-humans multiple dose trial, there was no way to know *a priori* how the pre-clinical dosing data would translate into human dosing until we conducted the present exploratory trial.

It is also worth re-emphasizing that the memantine, often used for symptomatic relief, here blocked all signals of bryostatin induced SIB improvement. Chronic memantine drug therapy has not been shown to have lasting benefit. Testing for the interaction of memantine baseline therapy with bryostatin efficacy was pre-specified (in the Statistical Analysis Plan) when the data were still blinded, prior to unblinding and data analysis. For the effective 20 μg dose, in the absence of baseline memantine, only 1 in 16 patients showed a SIB decline for the week 13–week 15-week endpoint. In contrast, 9 in 22 patients in the patient group receiving 20 μg bryostatin while on memantine, and 20 in 36 placebo patients showed a SIB decline. The principle targets of bryostatin, PKC isozymes, are known to regulate NMDA receptor functions, which are blocked by memantine. Therefore, it is not surprising that the blockade of the NMDA receptor could offset most if not all the bryostatin treatment effect. PKC regulation of the NMDA receptor functions includes increasing NMDA conductance by relieving Mg++ blockade, controlling trafficking of the NMDA receptor to the neuronal membranes, and enhancing NMDA-induced synaptogenesis. This synaptogenesis, a primary mechanism of action of bryostatin demonstrated in a variety of pre-clinical models, is mediated by bryostatin-PKC epsilon enhancement of several synaptic growth factors that include BDNF, NGF, and IGF.

While the memantine interaction with bryostatin adds complexity to the potential benefit of bryostatin for AD patients, we would submit that it also provides some additional evidence for this potential benefit. Namely, an effect of bryostatin that occurred only by chance would not be likely to be eliminated entirely in only patients who received SOC memantine (see Fig. 5A, B).

The apparent persistence of the bryostatin-induced SIB improvement signals is consistent with a long-lasting consequence of PKC epsilon-growth factor effects that could induce the growth and/or maturation of synaptic networks in the brain. This might translate into long-lasting benefit in cognitive function.

Although the analyses of the primary endpoint at 13 weeks was not significant for the full data set (FAS), the data did provide evidence of bryostatin’s improvement signals of the SIB scores at 13 weeks for the Completers Set and for both data sets at 15 weeks, 30 days after drug dosing completion. Pre-specified exploratory analyses, moreover, although, in some cases, implemented in a *post-hoc* framework, did provide evidence of significant benefit throughout the lower dose (20 μg) protocol. The totality of these analyses, therefore, suggest that the trial showed evidence of bryostatin’s SIB improvement signals, in the absence of baseline memantine, that warrant further trials to evaluate bryostatin’s potential utility to improve cognitive function(s) as well as to provide symptomatic relief and/or to delay cognitive decline of patients with moderately severe to severe AD.

## Supplementary Material

Supplementary Material 1

Supplementary Material 2
