# Supplementary material for: A Randomized, Double-Blind, Placebo-Controlled, Phase II Study Assessing Safety, Tolerability, and Efficacy of Bryostatin in the Treatment of Moderately Severe to Severe Alzheimer’s Disease
Source: J Alzheimers Dis. 2019 Jan 22;67(2):555–70. doi: 10.3233/JAD-180759 (PMC6398557; doi:10.3233/JAD-180759)
Supplement: Supplementary Material 1 [file jad-67-jad180759-s001.pdf]

STUDY PROTOCOL

A Randomized, Double-Blind, Placebo-Controlled, Phase 2 Study Assessing The Safety, Tolerability and Efficacy of Bryostatin in the Treatment of Moderately Severe to Severe Alzheimer's Disease

Protocol Number:	NTRP-101-202
Amendment Date:	3 March 2017
Study Drug:	Bryostatin 1; matching Placebo
Investigational new Drug Application (IND) Number:	71,276
Version:	4.0
Planned FPI to LPLV	Jan 2016 to Feb 2017
Study Sponsor:	Neurotrope BioScience, Inc.

Regulatory Statement

This study will be performed in compliance with the protocol and in accordance with Good Clinical Practice (GCP) (International Conference on Harmonisation [ICH], Guidance E6, 1996), principles of human subject protection, and applicable country-specific regulatory requirements.

Confidentiality Statement

The information in this document contains trade secrets and commercial information that are privileged or confidential and may not be disclosed unless such disclosure is required by applicable laws and regulations. In any event, persons for whom this information is disclosed must be informed that the information is privileged or confidential and may not be further disclosed by them. These restrictions on disclosure will apply equally to all future information supplied to you that is indicated as privileged or confidential

SPONSOR'S SIGNATURE PAGE

Title:

A Randomized, Double-Blind, Placebo-Controlled, Phase 2 Study Assessing Safety, Tolerability and Efficacy of Bryostatin in the Treatment of Moderately Severe to Severe Alzheimer's Disease

Protocol Number: NTRP-101-202

Version: 4.0 March 3, 2017

Sponsor's Authorized Representatives:

Kenneth Gorelick, MD	Acting Chief Medical Officer<br>Neurotrope BioScience, Inc.
______________________	______________________
Signature	Date
David Crockford	Vice President, Regulatory Affairs<br>Neurotrope BioScience, Inc.
______________________	______________________
Signature	Date
Elaine Grenier	Director, Head of Clinical Operations<br>Neurotrope BioScience, Inc
______________________	______________________
Signature	Date

PRINCIPAL INVESTIGATOR'S (PI) SIGNATURE PAGE

Protocol Title: A Randomized, Double-Blind, Placebo-Controlled, Phase 2 Study Assessing the Safety, Tolerability and Efficacy of Bryostatin in the Treatment of Moderately Severe to Severe Alzheimer's Disease

Protocol Number: NTRP-101-202
Protocol Version and Date: Version 4.0 March 3, 2017

Investigational Medicinal Product: Bryostatin 1

PI'S STATEMENT OF APPROVAL

Confidentiality of all information received or developed in connection with this protocol will be maintained by me, as well as all other personnel involved in the study who are employed by me. By signing this protocol, I confirm that I have read and agree to conduct the study as outlined in the protocol and in compliance with Good Clinical Practice, the Declaration of Helsinki as amended and all other applicable regulatory requirements.

Printed name of PI: ____________________________________

____________________________________ ______________________________

Signature Date

STUDY CONTACT INFORMATION

Sponsor :	Neurotrope BioScience, Inc.<br>205 East 42nd Street, 16th Floor<br>New York, NY 10017<br><br>Phone : (973)242-0005<br>Fax : (973)242-0009
Kenneth Gorelick, MD	Acting Chief Medical Officer<br>Neurotrope BioScience, Inc.<br><br>Mobile: 610-659-8236<br>Email: KGorelick@neurotropebioscience.com
David Crockford	Vice President, Regulatory Affairs<br>Neurotrope BioScience, Inc.<br><br>Mobile: (978) 992-1445<br>Email dcrockford@neurotropebioscience.com
Elaine Grenier	Director, Head of Clinical Operations<br>Neurotrope BioScience, Inc.<br><br>Mobile: (415) 548-0368<br>Email: egrenier@neurotropebioscience.com

Study Administrative Structure

Clinical Research Organization	Worldwide Clinical Trials<br><br>Operational Headquarters<br>1000 Continental Drive<br>Suite 290<br>King of Prussia, PA 19406<br>Tel: 610 964 2000<br>Corporate Headquarters<br>401 N Maple Drive<br>Beverly Hills, CA 90210<br>Tel: (310) 786-9100
Study Medical Monitor/Emergency Contact Information	Idil Cavus, MD PhD<br>Worldwide Clinical Trials<br>Mobile: (203) 623-2622<br>Fax: (203) 779-5678<br>E-mail: idil.avus@wwctrials.com
Central Laboratory	ACM Global<br>160 Elmgrove Park<br>Rochester, NY 14624 USA
Central ECG	eResearch Technology, Inc. (ERT)<br>1818 Market Street, Suite 1000<br>Philadelphia, PA 19103-3638<br>Tel: (215) 282-5303
Pharmacokinetic and Pharmacodynamic Plasma Sample Analysis Laboratory	Intertek Pharmaceutical Services<br>10420 Wateridge Circle<br>San Diego, CA 92121<br>(858) 558-2599, x241<br><br>Blanchette Rockefeller Neurosciences Institute<br>Eight Medical Center Drive<br>Morgantown, WV 26505-3409<br>(304) 293-0930

PROTOCOL SYNOPSIS

Name of Company: Neurotrope BioScience, Inc.	Name of Study Medication: Bryostatin 1	Name of Active Ingredient: Bryostatin 1
**Title of Study:**<br><br>A Randomized, Double-Blind, Placebo-Controlled, Phase 2 Study Assessing The Safety, Tolerability and Efficacy of Bryostatin in the Treatment of Moderately Severe to Severe Alzheimer's Disease		
**Study center(s):** Approximately 30 sites in United States (US)		
**Protocol Number:** NTRP101-202		
**Study Duration:** Approximately 14 months		**Study Phase:** 2
**Objectives:**<br><br>**Primary objective:**<br>To evaluate the safety and tolerability of bryostatin 1 (hereafter referred to as bryostatin) for the treatment of moderately severe to severe Alzheimer's disease (AD).<br><br>**Secondary objective:**<br>To evaluate the efficacy of bryostatin in the treatment of moderately severe to severe AD.<br><br>**Exploratory objectives:** To characterize the pharmacokinetics of bryostatin in subjects with AD following administration of 20 or 40µg of bryostatin administered as a 45-minute intravenous (IV) infusion over 12 weeks.<br><br>To characterize the pharmacodynamics of bryostatin in subjects with AD following administration of 20 or 40µg of bryostatin administered as a 45-minute infusion over 12 weeks.		
**Study Drug:** Bryostatin 1 or matching Placebo for Infusion<br>Drug is administered IV over 45(±5) minutes.		
**Number of Subjects:** 150 randomized subjects (approximately 250 subjects screened)		
**Study Design**<br><br>This is a randomized double-blind Placebo-controlled, Phase 2 exploratory study comparing two different doses of bryostatin to placebo for the treatment of moderately severe to severe Alzheimer's disease. The study is 15 weeks in duration, including a safety and efficacy evaluation 30 days after the last dose of study drug. Subjects will receive 7 doses of study drug during the study. The primary efficacy endpoint is the change from baseline in the Severe Impairment Battery (SIB) score after 12 weeks of treatment (at Week 13).		

Name of Company: Neurotrope BioScience, Inc.	Name of Study Medication: Bryostatin 1	Name of Active Ingredient: Bryostatin 1

Randomization and Treatment

Eligible subjects will be stratified based on Mini Mental State Exam (MMSE-2) scores 4-9 vs. 10-15 and will be randomized 1:1:1 to one of three treatment arms: 20µg, 40µg or placebo for twelve weeks. Study drug is administered IV by continuous infusion. The first two doses of each active treatment arm will be a loading dose 20% higher than the assigned dose (24µg, 48µg) and will be administered one week apart. Thereafter, the assigned dose of either 20µg, 40µg will commence with the third dose and be administered every other week. Subjects are scheduled to receive seven doses over 12 weeks. Subjects who drop out prior to completing the Week 7 visit will be replaced, up to a maximum of 15 subjects.

Evaluation Methods

Safety and tolerability will be determined through evaluations of adverse events (AE), serious adverse events (SAE), physical examination (PE), vital signs, 12-lead electrocardiogram (ECG), the Columbia Suicide Severity Rating Scale (C-SSRS), and clinical laboratory assessments.

Primary Safety End Points
Treatment emergent AEs and SAEs

Secondary Safety End Points
- Vital signs, hematology, blood chemistry, PE including body weight
- ECG parameters.
- C-SSRS

Primary Efficacy End Point
Change from baseline in the SIB Scale Score at Week 13

Secondary Efficacy End Points
- Change from baseline in the SIB at Weeks 5 and 9
- Change from baseline in Alzheimer Disease Cooperative Study Activities of Daily Living Inventory-Severe Impairment Version (ADCS-ADL-SIV) score at Weeks 5, 9 and 13
- Change from baseline in MMSE-2 score at Weeks 5, 9 and 13
- Change from baseline in Neuropsychiatric Inventory (NPI) score at Weeks 5, 9 and 13
- Clinical Global Impression of Improvement (CGI-I) at Weeks 5, 9 and 13

Exploratory Pharmacokinetic and Pharmacodynamic End Points:
- Determine a pharmacokinetic model for bryostatin plasma concentrations and summarize bryostatin exposure in plasma

Name of Company: Neurotrope BioScience, Inc.	Name of Study Medication: Bryostatin 1	Name of Active Ingredient: Bryostatin 1

- Peripheral blood mononuclear cell (PBMC) Protein Kinase C epsilon (PKCε) amount at various time points may be determined.

Statistical Considerations:

The primary statistical objective for efficacy is to estimate the effect of bryostatin on the mean change in the SIB after 12 weeks of treatment. A linear model will be used for both estimation and significance testing. This will be a mixed-model for repeated measures (MMRM) with fixed effects for treatment, baseline MMSE-2 stratum, scheduled visit (class variable) and scheduled visit by treatment interaction, plus a random effect for subject. The baseline efficacy measurement (SIB for the primary) will be a covariate. The primary contrasts will be the change from baseline in the SIB at Week 13 between each bryostatin group and the placebo group and between the combined (pooled) bryostatin dosing group and the placebo group. Inferences will be based on the estimates of expected values (least squares means (LSM) by treatment by scheduled visit derived from the model.

It is estimated that 150 subjects equally randomized among the three dosing groups (two doses of bryostatin and placebo administered over 12 weeks) will provide at least 80% power, assuming a true mean difference of at least 6.5 points in favor of the bryostatin groups (standardized effect size of 0.47), in a test between each bryostatin group and placebo in mean change from baseline in the SIB, (one-sided at α=0.10). This assumes the standard deviation of change from baseline at Week 13 is 14 points. This estimate allows that up to 15% of randomized subjects will not provide information due to discontinuation. The least significant difference for the test between the observed means under these conditions is estimated to be 3.9 points (effect size of 0.28).

Efficacy analyses will be conducted according to randomized groups. The full analysis set, consistent with the intention-to-treat principles, will be defined as all randomized subjects who received at least one dose of randomized study medication and who had at least one post-baseline assessment.

Adverse event and other safety data will be analyzed descriptively in all subjects who received any dose of study drug (including partial infusions). These data will be summarized by treatment group and by time in study.

Eligibility Criteria:

Inclusion

1. Written informed consent from caregiver and subject (if possible) or legally acceptable representative if different from caregiver
2. Male and female subjects 55-85 years of age inclusive

3. Cognitive deficit present for at least 2 years that meet the diagnostic criteria for probable Alzheimer's dementia.[1] The diagnosis must be confirmed at the time of the screening visit
4. MMSE-2 score of 4-15 inclusive
5. Patients must be able to perform at least one item on the SIB
6. Neuroimaging computerized tomography (CT) or Magnetic Resonance Imaging (MRI) within the last 24 months consistent with a diagnosis of probable AD without any other clinically significant co-morbid pathologies. If there has been a significant change in the subject's clinical status since the last imaging study that is not consistent with progression of the subject's AD an imaging study should be performed to confirm eligibility
7. Reliable caregiver(s) or informant(s) who attends the subject at least an average of 3 hours or more per day for 3 or more days per week and who will agree to accompany the subject to the clinic visits and reliably complete the caregiver questions
8. Adequate vision and motor function to comply with testing
9. If taking drugs approved for treatment of Alzheimer's disease (e.g. cholinesterase inhibitors, memantine), must be on a stable dose for at least 3 months prior to entry into study and the dose must not change during the study unless a change is required due to an adverse effect of the prescribed medication or a clinically significant change in the patient's status
10. Subjects on neuroleptic medications must be on a stable dose for ≥4 weeks (dose adjustments will be permitted)
11. Females participating in the study must meet one the following criteria:
    a. Surgically sterilized (e.g., hysterectomy, bilateral oophorectomy or tubal ligation) for at least 6 months or postmenopausal (postmenopausal females must have no menstrual bleeding for at least 1 year) or
    b. If not postmenopausal, agree to use a double method of contraception, one of which is a barrier method (e.g., intrauterine device plus condom, spermicidal gel plus condom) 30 days prior to dosing until 30 days after last dose and have negative human chorionic gonadotropin (β-hCG) test for pregnancy at screening
12. Males who have not had a vasectomy must use appropriate contraception methods (barrier or abstinence) from 30 days prior to dosing until 30 days after last dose
13. In the opinion of the PI subjects should be in reasonably good health over the last 6 months and any chronic disease should be stable

Exclusion

1. Dementia due to any condition other than AD, including vascular dementia (Rosen-Modified Hachinski Ischemic score ≥ 5)
2. Evidence of significant central nervous system (CNS) vascular disease on previous neuroimaging including but not limited to: cortical stroke, multiple infarcts, localized

single infarcts in the thalamus, angular gyrus, multiple lacunar infarcts or extensive white matter injury

3. Clinically significant neurologic disease or condition other than AD, such as cerebral tumor, chronic subdural fluid collections, Huntington's Disease, Parkinson's Disease, normal pressure hydrocephalus, or any other diagnosis that could interfere with assessment of safety and efficacy
4. Evidence of clinically significant unstable cardiovascular, pulmonary, renal, hepatic, gastrointestinal, neurologic, or metabolic disease within the 6 months prior to enrollment
5. Creatinine clearance (CL) of <45ml/min
6. Poorly controlled diabetes, at the discretion of the Principal Investigator
7. Use of vitamin E > 400 International Units (IU) per day within 14 days prior to screening
8. Use of valproic acid within 14 days prior to screening
9. Use of an active Alzheimer's vaccine within 2 years prior to screening
10. Use of a monoclonal antibody for treatment of AD within 1 year prior to screening
11. Any medical or psychiatric condition that is likely to require initiation of additional medication or surgical intervention during the course of the study
12. Any screening laboratory values outside the reference ranges that are deemed clinically significant by the PI
13. Use of an investigational drug within 30 days prior to screening
14. Suicidality defined as active suicidal thoughts during the 6 months prior to screening or at Baseline [Type 4 or 5 on C-SSRS], or history of suicide attempt in previous 2 years, or at serious suicide risk in PI's judgment
15. Major psychiatric illness such as current major depression according to Diagnostic and Statistical Manual of Mental Disorders, 5th Edition[2], current or past diagnosis of bipolar disorder, schizophrenia, or any other psychiatric disorder that might interfere with the assessments of safety or efficacy at the discretion of the PI
16. Diagnosis of alcohol or drug abuse within the last 2 years
17. Abnormal laboratory tests that suggest an alternate etiology for dementia. If the patient has prior history of serum B12 abnormality, anemia with hemoglobin ≤10g /dl, thyroid function abnormality, electrolyte abnormality, or positive syphilis serology the patient should be revaluated to determine if these potential causes of dementia have been addressed. Only if these causes have been ruled out as the cause of the dementia can the patient be enrolled.
18. History of prolonged QT or prolonged QT on screening ECG (QTcB or QTcF >499 per central reader)
19. Acute or poorly controlled medical illness: blood pressure > 180 mmHg systolic or 100 mmHg diastolic; myocardial infarction within 6 months; uncompensated congestive heart failure [New York Heart Association (NYHA) Class III or IV][3]
20. Known to be seropositive for human immunodeficiency virus (HIV)

21. Known to be seropositive for Hepatitis B or C
22. AST or ALT >3x upper limit of normal (ULN) and total bilirubin >2x ULN or International Normalized Ratio (INR) >1.5
23. Prior exposure to bryostatin, or known sensitivity to bryostatin or any ingredient in the study drug
24. Any other concurrent medical condition, which in the opinion of the PI makes the subject unsuitable for the clinical study

Table of Contents

SPONSOR'S SIGNATURE PAGE **2**
STUDY CONTACT INFORMATION **4**
STUDY ADMINISTRATIVE STRUCTURE **5**
PROTOCOL SYNOPSIS **6**
LIST OFABREVIATIONS **15**
1 INTRODUCTION **18**
1.0 Alzheimer's disease 18
1.1 Rationale for the use of bryostatin in the treatment of AD 19
1.2 Pharmacokinetics, Toxicology and Drug Metabolism in Animals 19
1.3 Genotoxicity 20
2 CLINICAL TRIAL DATA **20**
2.0 Oncology Data 20
2.1 Alzheimer's disease data 21
2.1.1 Study NTRP101-201 21
3 STUDY OBJECTIVES AND HYPOTHESIS **22**
3.0 Objectives 22
3.0.1 Primary objective: 22
3.0.2 Secondary Objectives 23
3.0.3 Exploratory Objectives: 23
3.1 Hypothesis 23
4 INVESTIGATIONAL PLAN **23**
4.0 Overview of Study Design 23
4.1 Dose Rationale 24
4.2 Risk/Benefit 25
4.3 Study Endpoints 26
4.3.1 Primary Safety End Points 26
4.3.2 Secondary Safety End Points 26
4.3.3 Primary Efficacy End Point 26
4.3.4 Secondary Efficacy End Points 26
4.3.5 Exploratory Pharmacokinetic and Pharmacodynamic End Points: 27
4.4 Study Population 27
4.4.1 Inclusion Criteria 27
4.4.2 Exclusion Criteria 28
5 PRODUCTS USED IN THIS STUDY **29**
5.0 Bryostatin 29
5.1 Placebo 30
5.2 Packaging and Labeling of Study Drug Kits 30
5.3 Storage and Preparation of Study Drug 30
5.4 Study Drug Accountability and Disposal 31
5.5 Randomization 31
5.6 Blinding 32
5.7 Study Drug Administration 32

6 STUDY PROCEDURES AND ASSESSMENTS 32
6.0 Table 1 Schedule of Activities 33
6.1 Assessments 35
6.1.1 Safety 35
6.1.2 Efficacy / Psychometric Assessments 37
6.1.3 Pharmacokinetic and Pharmacodynamic Assessments 38
6.2 Visit Procedures 38
6.2.1 Screening and Randomization (Days -28 to -2) 38
6.2.2 Week 0 (Day 0 Dose 1) 39
6.2.3 Week 1 (Day 7 (±2 days) Dose 2) 40
6.2.4 Week 2 (Day 14 (±2 days) No dose) 40
6.2.5 Week3 (Day 21 (±2 days)/ Dose 3) 40
6.2.6 Week 5 (Day 35 (±2 days)/ Dose 4) 41
6.2.7 Week 7 (Day 49 (±2 days)/ Dose 5) 41
6.2.8 Week 9 (Day 63 (±2 days)/ Dose 6) 41
6.2.9 Week 11 (Day 77(±2 days)/ Dose 7) 42
6.2.10 Week 13 (Day 91 (±2 days) 42
6.2.11 30-day Follow-up Visit 43
6.3 Concomitant Medications 43
6.3.1 Medications for AD 43
6.3.2 Concomitant Medications for Management of Myalgia 44
6.3.3 Prohibited Medications 44
7 ADVERSE EVENTS AND OTHER SAFETY EVALUATIONS 45
7.0 Definition of Adverse Events 45
7.1 Adverse Event of Special Interest - Myalgia 45
7.2 Definition of Serious Adverse Event 45
7.3 Assessment of Intensity 46
7.4 Relationship to Study Drug 46
7.5 Table 2 Relationship of AE to Study Drug or Trial-Related Procedures 47
7.5.1 Unexpected Adverse Event 47
7.6 Reporting Adverse Events 47
7.6.1 SAE reports 48
7.6.2 Reporting to Regulatory Authorities 49
7.7 Criteria for Withdrawal of Subjects 49
7.8 Criteria for Permanent Discontinuation of Study Drug 49
7.9 Study discontinuation 50
8 INDEPENDENT DATA AND SAFETY MONITORING BOARD (DSMB) 50
9 DATA ANALYSIS / STATISTICAL METHODS 50
9.0 Sample Size Determination 51
9.1 Demographics, Baseline Characteristics and Disposition 51
9.2 Safety Analysis 51
9.3 Efficacy Analysis 52
9.3.1 Analysis of Primary Endpoint 52
9.3.2 Analysis of Secondary Endpoints 52
9.4 Pharmacokinetics 53
9.5 DSMB Safety Assessments 53

10 DATA MONITORING....53
10.0 Source Documentation....53
10.1 Study Documentation and Record Retention....54
10.2 Site Monitoring....54
10.3 Quality Assurance and Quality Control....55
10.4 Audits and Inspections....55
11 ETHICS....55
11.0 Institutional Review Board....55
11.1 Ethical Conduct of the Study....56
11.2 Written Informed Consent....56
12 STUDY MANAGEMENT....56
12.0 Data Collection and management....56
12.1 Data Quality Control....56
12.2 Data Management and Data Storage....57
12.3 Inspection of Records....57
12.4 Retention of Records....57
12.5 Confidentiality....58
12.6 Protocol Amendments....58
12.7 Protocol Deviations....58
12.8 Data Corrections....58
12.9 Insurance....59
13 PUBLICATION AND DISCLOSURE POLICY....59
14 APPENDIX 1 - RESTRICTED CONCOMITANT MEDICATIONS....60
15 APPENDIX 2 LIST OF REFERENCES....65
16 APPENDIX 3 SUMMARY OF CHANGES 66

LIST OFABBREVIATIONS

Abbreviation	Definition
Aβ	Beta-amyloid
AD	Alzheimer's Disease
ADCS-ADL-SIV	Alzheimer's Disease Cooperative Study – Activities of Daily Living - Severe Impairment Version
AE	Adverse Event
ALP	Alkaline Phosphatase
ALT	Alanine Amino Transferase
APP	Amyloid Precursor Protein
AST	Aspartate Amino Transferase
Aβ1-42	Beta-Amyloid 1-42
ARIA	Amyloid Related Imaging Abnormalities
βAPP	Beta-amyloid Precursor Protein
β-hCG	Human chorionic gonadotropin
BDNF	Brain-derived Neurotrophic Factor
BUN	Blood Urea Nitrogen
CBC	Complete Blood Count
C-CASA	Columbia-Classification Algorithm of Suicide Assessment
CFR	Code of Federal Regulations
CGI-I	Clinical Global Impression – Improvement
CL	Clearance
CNS	Central Nervous System
CPK	Creatine phosphokinase
CRC	Cancer Research Campaign
CSF	Cerebrospinal Fluid
CT	Computerized tomography
C-SSRS	Columbia Suicide Severity Rating Scale
DSMB	Data Safety Monitoring Board
DSC	Digit Symbol Coding
ECG	Electrocardiogram
eCRF	Electronic Case Report Form
EDC	Electronic Data Capture
EMG	Electromyography
ERB	Ethical Review Board
FAS	Full Analysis Set

Abbreviation	Definition
FDA	Food and Drug Administration
FLAIR	Fluid Attenuated Inversion Recovery
GCP	Good Clinical Practice
GGT	Gamma Glutamyl Transferase
GI	Gastrointestinal
GMP	Good Manufacturing Practice
GRE	Gradient Refocused Echo
IAP	Interim Analysis Plan
IB	Investigator Brochure
ICF	Informed Consent Form
ICH	International Conference on Harmonisation
IEC	Independent Ethics Committee
IND	Investigational New Drug
INR	International Normalized Ratio
IRB	Institutional Review Board
IwRS	Interactive Web Response System
ITT	Intention-To-Treat
IU	International Unit
IV	Intravenous
LDH	Lactate Dehydrogenase
LSM	Least-Squares Means
MAP	Mitogen Activated Protein
MMRM	Mixed Model for Repeated Measures
MMSE-2	Mini Mental State Examination
MRI	Magnetic Resonance Imaging
MTD	Maximum Tolerated Dose
NCI	National Cancer Institute
NYHA	New York Heart Association
NMDA	N-Methyl-D-Aspartate
NOAEL	No Adverse Effect Level
NPI	Neuropsychiatric Inventory
PAP	Pharmacokinetic Analysis Plan
PBMCs	Peripheral Blood Mononuclear Cells
PET	Tween 80 (polysorbate 80)
PI	Principal Investigator

Abbreviation	Definition
PK	Pharmacokinetics
PKC	Protein Kinase C
PKCε	Protein Kinase C Epsilon
PP	Per Protocol Analysis Set
PT,	Prothrombin Time
PTT,	Partial Prothrombin Time
PVC	Polyvinylchloride
QTcB	Corrected QTc – Bazett's formula
QTcF	Corrected QTc – Fridericia's formula
RBANS	Repeatable Battery of Assessments for Neuropsychological Status
SA	Safety Analysis Set
SAE	Serious Adverse Event
SAP	Statistical Analysis Plan
SD	Standard Deviation
SIB	Severe Impairment Battery
SUSAR	Suspected Unexpected Serious Adverse Reactions
ULN	Upper limit of normal
USP	United States Pharmacopeia
US	United States
WHO-DRL	World Health Organization Drug Reference List

1 INTRODUCTION

1.0 Alzheimer's disease

Alzheimer's disease is the most common cause of dementia, affecting approximately 5.3 million people in the United States (US) and 30 million people worldwide. There is a disproportionate representation of women with nearly 2/3 of the affected individuals being female. Of the 5.3 million affected Americans, 5.1 million are older than 65 (Alzheimer's Association website www.alz.org, 2015). Since aging is the single most important risk factor for development of dementia and medical advances are prolonging survival, the incidence of AD will increase. The US Census Bureau data suggest that the number of individuals living until age 100 between the years 2000 and 2020 will increase by more than 200% and the number of individuals living until age 90-95 will double.[4] By 2050 the number of individuals with Alzheimer's disease will almost triple to a projected 13.8 million. In 2013, 15.5 million family and friends provided 17.7 billion hours of unpaid care to those with Alzheimer's and other dementias – care valued at $220.2 billion (Alzheimer's Association website www.alz.org, 2015).

The currently approved treatments for AD provide modest symptomatic benefit and do not alter the disease progression. These therapies primarily consist of cholinesterase inhibitors [tacrine (Cognex), donepezil (Aricept), rivastigmine (Exelon), and galantamine (galanthamine, Reminyl)] and the N-methyl-D-aspartate (NMDA) antagonist, memantine (Namenda). The development of a medication that produces a significant improvement in clinical symptoms and/or slows the progression of the disease would be a significant advance in the therapeutics of AD.

The protein kinase C (PKC) signaling pathways have been shown to play an integral role in learning and memory. Several animal models have demonstrated that PKC is involved in the learning process.[5, 6] In addition, PKC activity regulates phosphorylation of Tau and cleavage of Aβ amyloid through its effects on alpha secretase and GSK3β.[13] The single most important risk factor for the sporadic form of AD, increased age, has also been linked to impaired PKC-mediated α-secretase activation.[7] Aged animal models, for example, have shown age-specific changes of PKC isozyme distribution in the brain,[8, 9] impaired PKC translocation, reduced levels of the PKC anchoring protein, RACK1,[10] alterations in mitogen activated protein (MAP) kinase Erk1/2,[11] and reduced levels of the α-secretase cleaved Amyloid Precursor Protein (APP), soluble amyloid precursor protein alpha (sAPPα), in the cerebrospinal fluid (CSF).[12] Aging of normal human fibroblasts also reduced secretion of sAPPα.

The demonstration of reduced levels of PKC in the brain of Alzheimer's subjects suggests a potential target to improve cognition in AD is the activation of PKC which in turn activates a series of downstream pathways that enhance synaptic function and promote synaptogenesis. Bryostatin is a potent stimulator of PKCε and is non-tumorigenic. Hongpaisan et.al [13] have recently demonstrated the cognitive benefits and the effect on synaptogenesis in two strains of transgenic AD mice. Their work was recently reproduced by Schrott, et.al.[14] Activation of PKCε by bryostatin was associated with reduced levels of β amyloid, increased levels of the Brain Derived Neurotropic Factor (BDNF), prevention of synapse loss, reduced plaque formation and restored memory and learning even in the presence of plaques.

1.1 Rationale for the use of bryostatin in the treatment of AD

The pharmacological basis for the use of bryostatin for the treatment of AD is based on the hypothesis that low dose, intermittent administration of bryostatin will activate PKC isozymes α and ε. Activation of PKC is associated with:

- Enhanced associative learning and recent memory via PKC-mediated phosphorylation of downstream substrates
- Increased synthesis of proteins such as MAP kinase Erk1/2, and synaptogenic proteins such as BDNF required for long-term memory
- Activation of α-secretases, thereby increasing non-toxic fragments of the beta-amyloid precursor protein (βAPP) and reducing the neurotoxic fragment of βAPP, $A\beta_{1-42}$, and the associated neuropathology
- Activation of $A\beta_{1-42}$ – degrading enzymes
- Phosphorylation of GSK-3 beta, inhibiting production of Tau and the associated neuropathology
- Improved memory and learning in 3 different strains of transgenic mice with single or multiple Alzheimer's gene mutations even in the presence of amyloid plaque [14, 13, 15]

Thus activation of PKC can enhance existing synaptic function, reduce the toxic effects of amyloid and promote synaptogenesis, all potential targets to improve cognitive function in AD.

1.2 Pharmacokinetics, Toxicology and Drug Metabolism in Animals

Single dose toxicity of bryostatin has been characterized in four non-GLP studies conducted under National Cancer Institute's (NCI) IND, including three IV toxicity studies in mice and one IV toxicity study in rats. Bryostatin was evaluated in a single GLP–repeated dose 21-day IV toxicity study in rats. The single dose toxicity studies were conducted using the ethanol/saline or the polyethylene glycol, ethanol, and Tween 80 (polysorbate 80) (PET) diluent. LD_{50} range from a low of 38µg/kg to 75µg/kg. The 21 day repeat-dose toxic study performed in rats at doses of 0, 10, 15 and 25µg/m^2 with the ethanol/saline formulation. There were no relevant toxicology findings. The maximum tolerated dose (MTD) and no adverse effect level (NOAEL) were noted to be 25µg/m^2 the highest dose studied. However since there were no toxicologically relevant findings, the report suggested that the MTD and NOAEL are greater than 25µg/m^2.

Limited pharmacokinetics (PK) data are available in animals. The pharmacokinetics of bryostatin was analyzed in female CD1/F2 mice by using [C26-3H]-labeled bryostatin following IV and IP administration (Zhang et al 1996). Following IV administration of 40µg/kg (120mg/m^2), the plasma disappearance curve for bryostatin could be described by a 2-compartment model, with a distribution $t_{½}$ of 1.05hours and an elimination $t_{½}$ of 22.96 hours. In contrast, following IP administration, the plasma disappearance curve was better described by a first-order absorption one-compartment model, with an absorption $t_{½}$ of 0.81hours and an elimination $t_{½}$ of 28.76hours.

Urinary excretion represented the major pathway of elimination in the first 12 hours after IV administration, with 23.0 ± 1.9% (mean ± standard deviation) of the administered dose excreted.

Approximately equal amounts of radioactivity (40%) were excreted in feces compared with urine within 72 hours after IV administration. A greater area under the curve, longer mean resident time, and lower clearance were observed with IP administration compared with IV administration. Bryostatin was widely distributed to various tissues following both IV and IP administration. However, accumulation was observed in the lung, liver, gastrointestinal (GI) tract, and fatty tissue.

1.3 Genotoxicity

Bryostatin was evaluated using the bacterial reverse mutation assay (i.e., the Ames test), no positive responses were observed. Bryostatin was evaluated as negative (nonclastogenic) in the micronucleus assay; it was also evaluated as negative (non-DNA damaging) in the Comet Assay. Overall, based on the results of the Ames test and the combined micronucleus/comet assay, bryostatin is not considered to be genotoxic.

2 CLINICAL TRIAL DATA

2.0 Oncology Data

Safety data are available from published clinical studies of bryostatin for the treatment of cancer. Altogether, over 1400 oncology subjects received bryostatin, mainly under NCI's IND # 42,780, with exposures to bryostatin in both single and combination agent studies. About 584 subjects received bryostatin as monotherapy, with dose levels ranging from 5μg/m^2 to >180μg/m^2. Most subjects in both the monotherapy and combination therapy studies received bryostatin at doses >25μg/m^2, most often as 1 hour infusions administered at various time intervals from weekly infusion to continuous infusions for 72 hours. Most studies were repeated dose studies where subjects received treatment for several weeks (See Investigators Brochure).

Adverse events occurring in the single agent studies that resulted in discontinuation from the studies were myalgia (28 subjects), acute transient reaction (dyspnea, flushing, hypotension, and bradycardia; 4 subjects each), phlebitis (attributed to ethanol in the formulations, 6 subjects), fatigue (3 subjects), and 1 subject each with bacteremia, chest pain, dehydration, dysphagia, hematuria, nausea, skin rash, subclavian vein thrombosis, thrombocytopenia, and vomiting. The following AEs were associated with death in the single agent trials: cardiac arrest (2), hypotensive with evidence of renal and hepatic failure (1), perforated gastric ulcer (1), renal function decline with cardiac arrest and perforated gastric ulcer (1; PIs considered not related to bryostatin), and sudden death (1; PIs considered likely to be cardiovascular event). Relatedness to bryostatin treatment was not assessed except where noted.

Other severe (Grade 3 or higher) AEs reported in the single agent clinical studies in cancer subjects included: alkaline phosphatase (ALP) (elevated; subject had pre-existing liver metastases), allergic reaction, anemia, anorexia, arthralgia, ataxia, cardiac arrhythmias, cardiovascular, coagulation, community-acquired pneumonia, congestive heart failure, constipation, dermatitis, dermatologic, diarrhea, dyspnea, edema/weight gain, fever, gastrointestinal, genitourinary, granulocytopenia, headache, hepatic, hyperbilirubinemia, hyperglycemia, hypokalemia, hyponatremia, infection, leg weakness, lymphedema, lymphocytopenia, myocardial infarction, neurotoxicity, neutropenia, pain (abdominal, back, eye, site not specified), pulmonary, pulmonary embolus from inferior vena cava tumor thrombus, sepsis and pneumonia without neutropenia, syncope, and urinary frequency.

The absence of a Placebo-control group in bryostatin oncology studies makes it difficult to determine the extent to which underlying disease or concomitant medications may have contributed to this safety profile.

2.1 Alzheimer's disease data

2.1.1 Study NTRP101-201

This was a randomized, double-blind, Placebo-controlled safety study of a single dose of bryostatin in subjects with mild to moderate AD (MMSE: 14-26). Subjects were randomized 2:1 to receive bryostatin 25µg/m^2 or Placebo. The primary objective was to evaluate the safety and tolerability of bryostatin by the incidence of AEs and SAEs. Secondary safety endpoints included assessment of physical examination, hematology including complete blood count (CBC) and platelet count, coagulation parameters, serum chemistries, ECG, urinalysis and vital signs.

The primary efficacy endpoint was a composite end point of change from baseline in the Hopkins Verbal Learning Test-Revised (HVLT-R) delayed recall and Repeatable Battery of Assessments for Neuropsychological Status (RBANS) figure recall at 48 hours post study drug infusion.

The study included single dose PK, and measurement of PKCε in peripheral blood mononuclear cells (PBMCs) as a potential biomarker.

2.1.1.1 Results

2.1.1.1.1 <u>Demographics</u>

The study enrolled nine subjects, 4 male and 5 female, with a mean age of 71.8 ±7.4 years (range 62 to 82). The mean MMSE-2 at baseline was 22.5 for the three placebo subjects (range 19-24) and 22 (range 16-26) for the six bryostatin treated subjects.

2.1.1.1.2 <u>Safety</u>

Bryostatin was well tolerated. There were no deaths or SAEs reported. No subjects had an AE leading to withdrawal during the study. There were five treatment emergent adverse events occurring in three subjects: headache, dizziness, and papular rash. There were no reported episodes of myalgia, a known side effect of bryostatin. The only adverse event in the bryostatin treated group was headache, which was not considered related to study drug. All adverse events were mild and resolved without treatment. All laboratory assessments, including hematology, chemistry, coagulation, renal function and liver function as well as cardiac assessments were unremarkable after treatment and there was no clinically significant change in any vital signs.

2.1.1.1.3 <u>Efficacy</u>

There was no difference in HVLT-R delayed recall or the RBANS delayed figure recall at 48 hours. Additional time points of assessment for these measures (day 2, day 4 and day 15) did not indicate any difference between groups. Additional endpoints included the change from baseline in Digit Symbol Coding (DSC), and MMSE-2 at various time points. There was no difference in mean values between

groups for these endpoints at any time point. Both the treatment and the Placebo group showed an improvement in the MMSE-2 score most likely due to practice effects since the MMSE-2 was administered five times in 2 weeks.

In summary, there was no clinically significant difference between the mean or mean change from baseline between bryostatin and placebo in the HVLT-R or MMSE-2 assessments or any other efficacy assessments to suggest a treatment effect after a single dose of 255μg/m^2 bryostatin.

2.1.1.1.4 Pharmacokinetics

The pharmacokinetics of bryostatin were assessed in 6 subjects following 25 μg/m^2 bryostatin administered as a single 1-hour IV infusion. Individual bryostatin plasma concentrations were observed to increase and approach steady-state within the 1 hour infusion periods, and then rapidly decrease following the end of the infusion. The bryostatin maximum plasma concentrations occurred at the end of the IV infusions, and had a mean (±standard deviation (SD)) value of 1.09 ±0.25 ng/mL. A terminal elimination rate could not be calculated for most subjects due to sampling frequency, the rapid drug elimination, and the sensitivity limitations of the assay. Based on the observed individual bryostatin plasma concentration profiles, the elimination $t_{1/2}$ associated with the observed drug exposure was estimated to be less than 30 minutes. Total drug CL was observed to be high (~ 40 L/h), consistent across the individual administered doses, and did not appear to be related to body weight, BSA, or sex. The bryostatin pharmacokinetic parameters were observed to have low to moderate intersubject variability.

2.1.1.1.5 Pharmacodynamics/ PKCε

Preliminary assessment of PKCε concentration in PBMCs suggests there is an increase in the total amount of PKCε (cytosol plus membrane bound concentration) following treatment with bryostatin. Additional analysis is underway to further characterize the increase and pharmacodynamics.

2.1.1.1.6 Conclusions

This is the first double-blind assessment of the safety of bryostatin treatment in subjects with Alzheimer's disease. The study met it primary endpoint of safety and tolerability. Bryostatin appears safe and well tolerated in this cohort of subjects with mild to moderate Alzheimer's disease. No improvement was detected in cognitive function on the efficacy measurements though the study was not powered to detect cognitive benefit. There was no evidence of an adverse effect on cognition following treatment with bryostatin.

3 STUDY OBJECTIVES AND HYPOTHESIS

3.0 Objectives

3.0.1 Primary objective:

- To evaluate the safety and tolerability of bryostatin for the treatment of moderately severe to severe Alzheimer's disease

3.0.2 Secondary Objectives

- To evaluate the efficacy of bryostatin in the treatment of moderately severe to severe Alzheimer's disease

3.0.3 Exploratory Objectives:

To characterize the pharmacokinetics of bryostatin in subjects with AD following administration of 20 or 40µg of bryostatin administered as a 45 minute IV infusion over 12 weeks.

To characterize the pharmacodynamics of bryostatin in subjects with AD following administration of 20 or 40µg of bryostatin administered as a 45 minute IV infusion over 12 weeks of treatment.

3.1 Hypothesis

Treatment with bryostatin will result in improvement of cognitive function as measured by improvement on performance on the SIB measured after 12 weeks of treatment compared to placebo. The primary efficacy endpoint will be tested against the null hypothesis: the change from baseline at Week 13 (after 12 weeks of treatment) of the SIB score in the bryostatin arms is equal to or less than the change from baseline of the SIB score in the placebo arm. The alternative hypothesis is: the change from baseline at Week 13 of the SIB score due to bryostatin is greater than the change from baseline of the SIB score due to placebo.

The null hypothesis will be rejected at a significance level of α (1-sided) = 0.10. This analysis will be a test of superiority and only an improvement in the SIB score is of interest. The analysis will be conducted using a mixed model for repeated measures (MMRM). Analyses will be performed on each bryostatin dosing group vs. placebo and on the "pooled" bryostatin dosing group vs. placebo.

4 INVESTIGATIONAL PLAN

4.0 Overview of Study Design

This is a randomized double-blind placebo-controlled exploratory study comparing different doses of bryostatin for the treatment of moderately severe to severe Alzheimer's disease. The study is 15 weeks in duration, including a safety and efficacy evaluation 30 days after the last treatment. Eligible subjects will be stratified based on MMSE-2 scores 4-9 vs. 10-15 and will be randomized 1:1:1 to one of three treatment arms: 20µg, 40µg or placebo for 12 weeks of treatment (see Figure 1). Study drug is administered IV by continuous infusion. The first two doses of each active treatment arm will be a loading dose 20% higher than the assigned dose (24µg, 48µg) and will be administered one week apart. The assigned dose of either 20µg or 40µg will commence with the third dose two weeks after the second loading dose and be administered every other week thereafter. Subjects are scheduled to receive seven doses over 12 weeks. Subjects who drop out prior to completing the Week 7 visit will be replaced, up to a maximum of 15 subjects. Safety and efficacy assessments will be done as indicated on the Schedule of Activities (Table 1).

Figure 1: Study Design

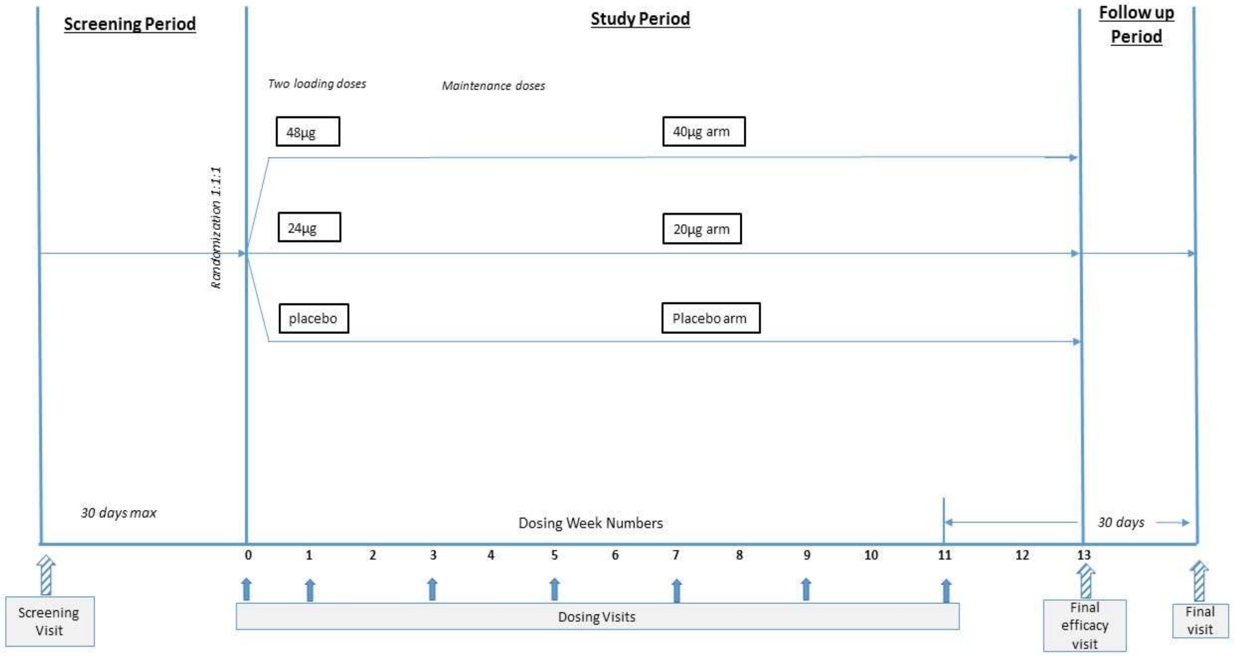

4.1 Dose Rationale

The primary objective of treatment for cognitive disorders with bryostatin is activation of PKCε, not downregulation as was attempted for the oncology indications. Bryostatin activation of PKCε is dose dependent, where lower doses activate PKCε and appear to increase de-novo synthesis of the enzyme, while higher doses of bryostatin downregulate PKCε. It is unclear where the inflection point for downregulation occurs, thus this study is designed to explore two doses of bryostatin at fixed dose levels and frequency that is thought to initiate and maintain activation of PKCε without downregulation.

The dosing regimen is based on existing human oncology data and the recently completed phase 2a PK and safety study and PKCε levels in peripheral monocytes that were measured after treatment with bryostatin in both programs.

The oncology data suggest that the dose-limiting side effect of myalgia is dose dependent and cumulative and likely related to the down regulation of PKCε. In the oncology studies where subjects received 75µg/m^2 to 125µg/m^2 of bryostatin over a 72-hour infusion, PKC levels were initially upregulated, but within 2-24 hours after start of the infusion, levels were down regulated for the remainder of the 72 hour of infusion. This was not seen in the Phase 2a safety study where AD subjects received a single 25µg/m^2 dose of bryostatin.

Preliminary assessment of PKCε total concentration in AD subjects (Phase 2a safety study) who received bryostatin as a single 25µg/m^2 dose demonstrated an increase in PKCε total protein. There were

no reports of myalgia in that study suggesting there was no down regulation. Thus the activation of PKCε can be achieved with a dose of 25µg/m^2.

The phase 2a PK data demonstrated that total drug CL was high (~ 40 L/h), was consistent across the individual administered doses, and did not appear to be related to body weight, BSA, or sex. In addition, bryostatin pharmacokinetic parameters were observed to have low to moderate inter-subject variability. Based on these findings, a fixed dose regime is being used in this protocol. Using 1.73 m^2 as standard body surface area, a 25µg/m^2 dose translates to a fixed dose of 43.25µg. To explore doses that can activate PKCε and not decrease the activity or concentration of the enzyme when repeated over time, doses of 40µg and 20µg will be explored. The fixed doses used in this study will provide bryostatin exposures in plasma that are similar to or lower than exposures associated with a bryostatin dose of 25 µg/m^2. In addition, fixed dosing will reduce the chance of dosing errors inherent in weight or body surface area dosing paradigms.

Dosing will begin with two initial loading doses at one week intervals that are 20% higher (48µg and 24µg respectively) than the maintenance doses to assure enzyme activation. Dosing will then continue every other week as noted in Figure 1.

4.2 Risk/Benefit

In Study NTRP101-201, a Phase 2a safety study, which included nine Alzheimer's disease subjects, six subjects received bryostatin. There were no safety signals identified following a single IV infusion of 25µg/m^2 of bryostatin; there were no SAES, and AEs were mild and self-limited. There were no laboratory or cardiac changes noted following treatment.

Multiple phase 1 and phase 2 oncology studies were conducted under NCI's IND for bryostatin using multiple dose regimens across a wide range of doses, as low as 5µg/m^2 up to 180µg/m^2 administered as infusions ranging from 1 to 72 hours duration. The only dose limiting toxicity that was observed was myalgia. The myalgia generally involved thigh, calf and ocular muscles. The myalgia appeared in incidence and severity to be related to both administered dose and cumulative dose.

The etiology of the myalgia is not clear. No increase in muscle enzymes was found in those subjects in whom it was studied. Electromyography (EMG) was abnormal in one subject who received 65µg/m^2, suggesting a patchy myositis; and MRI in another subject was normal. Almost all oncology studies were performed at higher doses than are planned in this protocol, and in some cases the oncology subjects were dosed at a higher frequency, including weekly for 3 weeks with one week off. The doses planned in this study, 20µg and 40µg,are generally below those administered in the oncology studies. Myalgia was self-limited lasting hours to days, depending on severity. The pain was relieved by over the counter analgesics such as acetaminophen. To protect subject's safety, myalgia is defined as an adverse event of special interest and a Data Safety Monitoring Board (DSMB) will review all safety data as described in Section 8 of this protocol with special attention to this dose limiting toxicity.

Headache and infusion reactions that include dyspnea, fever, flushing, hypotension, and bradycardia have been reported in the oncology studies, and as noted in one study lasted only 5-10 minutes. In the very early oncology studies, using a different formulation (60% ethanol/40% saline), phlebitis was reported at the injection site. It has also been reported across some studies with the present PET diluent.

Subjects will be monitored for infusion reactions and treated as needed. Pretreatment with antihistamines or steroids is not deemed necessary at this time but can be implemented if the need arises.

Therapeutic antibodies targeting β-amyloid have been associated with the occurrence of amyloid related imaging abnormalities (ARIA), which is similar in appearance to cerebral vasogenic edema and microhemorrhage. A minority of cases have been associated with symptoms of headache, visual disturbance, loss of coordination, or disorientation. Asymptomatic ARIA was reported in a clinical study of the small molecule gamma-secretase inhibitor BMS-708163. Bryostatin has a different mechanism of action than antibodies directed at amyloid, but in the transgenic mouse model of AD treated with bryostatin a decrease of Aβ deposition was observed. Given the limited bryostatin experience in AD, the potential for ARIA and associated clinical events cannot be excluded.

In the event that a subject develops findings suggestive of vasogenic edema or microhemorrhage, evaluation should include an MRI scan performed in accordance with Sperling 2011[16] including 2D T2* gradient refocused echo (GRE) for the detection of micro-hemorrhages and T2 Fluid Attenuated Inversion Recovery (FLAIR) for the detection of edema. Should evidence of edema or micro-hemorrhage be observed in association with clinical symptoms, study drug should be discontinued. ARIA cases have often resolved despite continued study drug, and no specific treatments for ARIA have been confirmed as necessary or useful, but intravenous corticosteroids may be considered in the event of severe symptoms and/or severe edema.

Subjects in this study, with moderately severe to severe dementia, are those who have had a progressive dementia to the degree that their activities of daily living and their ability to care for themselves may be compromised. Subjects with MMSE-2 scores of 15 or less display significant cognitive impairments that are disabling. Most if not all of these subjects will have progressed despite treatment and have no alternative treatments available. Based on the available nonclinical and clinical data, and the low risk of ARIA, the potential benefits of treatment with bryostatin at the proposed doses in this protocol outweigh the risks.

4.3 Study Endpoints

4.3.1 Primary Safety End Points

Treatment emergent AEs and SAEs

4.3.2 Secondary Safety End Points

1. Vital signs, hematology, blood chemistry, and physical examination including body weight
2. ECG parameters
3. C-SSRS

4.3.3 Primary Efficacy End Point

1. Change from baseline in the SIB at Week 13

4.3.4 Secondary Efficacy End Points

1. Change from baseline in the SIB at Weeks 5 and 9

2. Change from baseline in ADCS-ADL-SIV score at Weeks 5, 9 and 13
3. Change from baseline in MMSE-2 score at Weeks 5, 9 and 13
4. Change from baseline in NPI score at Weeks 5, 9 and 13
5. CGI-I at Weeks 5, 9 and 13

4.3.5 Exploratory Pharmacokinetic and Pharmacodynamic End Points:

1. Cmax, Tmax, AUClast, AUCinf, λz, T1/2, CL, Vz
2. Peripheral blood mononuclear cell PKCε amount may be assessed at various time points

4.4 Study Population

Subjects with moderately severe to severe Alzheimer's disease defined as a MMSE-2 score of 4-15 inclusive are eligible to enroll. Subjects will be permitted to continue present FDA-approved treatments for Alzheimer's disease but no new treatments can be initiated. OTC medications taken for cognitive improvement such as Ginkgo Biloba or other empiric medications are permitted while participating in the study but should not be initiated or dose modified after screening. Subjects who are no longer on medications for Alzheimer's disease can be enrolled.

4.4.1 Inclusion Criteria

1. Written informed consent from caregiver and subject (if possible) or legally acceptable representative if different from caregiver
2. Male and female subjects 55-85 years of age inclusive
3. Cognitive deficit present for at least 2 years that meet the diagnostic criteria for probable Alzheimer's dementia[1]. The diagnosis must be confirmed at the time of the screening visit
4. MMSE-2 score of 4-15 inclusive
5. Patients must be able to perform at least one item on the SIB
6. Neuroimaging (CT or MRI) within the last 24 months consistent with a diagnosis of probable AD without any other clinically significant co-morbid pathologies. If there has been a significant change in the subject's clinical status since the last imaging study that is not consistent with progression of their AD an imaging study should be performed to confirm eligibility
7. Reliable caregiver(s) or informant(s) who attends the subject at least an average of 3 hours or more per day for 3 or more days per week and who will agree to accompany the subject to the clinic visits and reliably complete the caregiver questions
8. Adequate vision and motor function to comply with testing
9. If taking drugs approved for treatment of Alzheimer's disease (e.g. cholinesterase inhibitors, memantine), must be on a stable dose for at least 3 months prior to entry into study and dose must not change during the study unless a change is required due to an adverse effect of the prescribed medication or a clinically significant change in the patient's status
10. Subjects on neuroleptic medications must be on a stable dose for ≥4 weeks (dose adjustments will be permitted)

11. Females participating in the study must meet one the following criteria:
    a. Surgically sterilized (e.g., hysterectomy, bilateral oophorectomy or tubal ligation) for at least 6 months or postmenopausal (postmenopausal females must have no menstrual bleeding for at least 1 year) or
    b. If not postmenopausal, agree to use a double method of contraception, one of which is a barrier method (e.g., intrauterine device plus condom, spermicidal gel plus condom) 30 days prior to dosing until 30 days after last dose and have negative β-hCG test for pregnancy at screening
12. Males who have not had a vasectomy must use appropriate contraception methods (barrier or abstinence) from 30 days prior to dosing until 30 days after last dose
13. In the opinion of the PI subjects should be in reasonably good health over the last 6 months and any chronic disease should be stable.

4.4.2 Exclusion Criteria

1. Dementia due to any condition other than AD, including vascular dementia (Rosen-Modified Hachinski Ischemic score ≥ 5)
2. Evidence of significant central nervous system (CNS) vascular disease on previous neuroimaging including but not limited to: cortical stroke, multiple infarcts, localized single infarcts in the in the thalamus, angular gyrus, multiple lacunar infarcts or extensive white matter injury
3. Clinically significant neurologic disease or condition other than AD, such as cerebral tumor, chronic subdural fluid collections, Huntington's Disease, Parkinson's Disease, normal pressure hydrocephalus, or any other diagnosis that could interfere with assessment of safety and efficacy
4. Evidence of clinically significant unstable cardiovascular, pulmonary, renal, hepatic, gastrointestinal, neurologic, or metabolic disease within the 6 months prior to enrollment
5. Creatinine CL of <45ml/min
6. Poorly controlled diabetes, at the discretion of the Principal Investigator
7. Use of vitamin E > 400 IU per day within 14 days prior to screening
8. Use of valproic acid within 14 days prior to screening
9. Use of an active Alzheimer's vaccine within 2 years prior to screening
10. Use of a monoclonal antibody for treatment of AD within 1 year prior to screening
11. Any medical or psychiatric condition that is likely to require initiation of additional medication or surgical intervention during the course of the study
12. Any screening laboratory values outside the reference ranges that are deemed clinically significant by the PI
13. Use of an investigational drug within 30 days prior to screening

14. Suicidality defined as active suicidal thoughts during the 6 months prior to the screening or at Baseline [Type 4 or 5 on C-SSRS scale], or history of suicide attempt in previous 2 years, or at serious suicide risk in PI's judgment
15. Major psychiatric illness such as current major depression according to Diagnostic and Statistical Manual of Mental Disorders, 5th Edition, current or past diagnosis of bipolar disorder, schizophrenia, or any other psychiatric disorder that might interfere with the assessments of safety or efficacy at the discretion of the PI
16. Diagnosis of alcohol or drug abuse within the last 2 years
17. Abnormal laboratory tests that suggest an alternate etiology for dementia. If the patient has prior history of serum B12 abnormality, anemia with hemoglobin ≤10g /dl, thyroid function abnormality, electrolyte abnormality, or positive syphilis serology the patient should be revaluated to determine if these potential causes of dementia have been addressed. Only if these causes have been ruled out as the cause of the dementia can the patient be enrolled.
18. History of prolonged QT or prolonged QT on screening ECG (QTcB or QTcF >499 per central reader)
19. Acute or poorly controlled medical illness: blood pressure > 180 mmHg systolic or 100 mmHg diastolic; myocardial infarction within 6 months; uncompensated congestive heart failure [New York Heart Association (NYHA) Class III or IV]. History of cancer: the subject should be clear of cancer for at least 2 years prior to screening
20. Known to be seropositive for human immunodeficiency virus (HIV)
21. Known to be seropositive for Hepatitis B or C
22. Aspartate Amino Transferase (AST) or Alanine Amino Transferase (ALT) >3x ULN and total bilirubin >2x ULN or INR >1.5
23. Prior exposure to bryostatin, or known sensitivity (allergy) to bryostatin or any ingredient in the study drug
24. Any other concurrent medical condition, which in the opinion of the PI makes the subject unsuitable for the clinical study.

Subjects who are screen-failed (e.g. due to clinically significant laboratory abnormality or an active medical condition) may be re-screened if their medical condition stabilizes or improves as assessed by the PI. Subjects with CL<60ml/min should be referred for follow-up by their primary care provider.

5 PRODUCTS USED IN THIS STUDY

Active study drug, bryostatin, and a matching placebo will be provided as described below.

5.0 Bryostatin

The investigational drug product, bryostatin, is a sterile, pyrogen-free, lyophilized powder intended for IV infusion upon reconstitution and dilution. Bryostatin will be supplied in a 10 mL vial containing 0.05mg bryostatin, 2.5mg povidone lyophilized from 40% t-butanol. Accompanying each vial of

Bryostatin will be a 10mL vial containing 2mL of sterile PET diluent [60% v/v polyethylene glycol 400, 30% v/v dehydrated ethyl alcohol, and 10% v/v Tween-80 (polysorbate 80)].

5.1 Placebo

The placebo is a sterile, pyrogen-free lyophilized powder intended for IV infusion upon reconstitution and dilution. It will be supplied in a 10 mL vial containing 0.0mg bryostatin, 2.5mg povidone lyophilized from 40% t-butanol. Accompanying each vial of placebo will be a 10 mL vial containing 2mL of the same sterile PET diluent as described above for bryostatin. The placebo is identical to bryostatin in appearance, including color, consistency and odor.

5.2 Packaging and Labeling of Study Drug Kits

Study drug kits will contain 7 vials of Bryostatin for Infusion or 7 vials of Placebo for Infusion and 7 vials of PET diluent. Each kit will be identified by kit number and assigned to a subject via the Interactive Web Response System (IwRS), according to the established randomization scheme.

Study drug kits will be labeled in English according to the US FDA's current Good Manufacturing Practice (GMP) and local regulations. The label will contain the following information:

1. Bryostatin or Placebo for Infusion Kit
2. Kit number
3. Store refrigerated at 2-8°C.
4. A description of kit contents
5. Caution: Investigational New Drug - Limited by Federal (US) Law to Investigational Use Only
6. Sponsor's manufacturer

5.3 Storage and Preparation of Study Drug

The study drug kits will be stored under refrigeration (2-8°C) in a refrigerator, refrigerated cabinet or other refrigerated enclosure, which is securely locked and temperature monitored. Access to the stored study drug kits will be restricted to the investigational site pharmacy or designated unblinded staff member.

Study drug will be prepared and dispensed for administration by a qualified, unblinded member of the study staff (e.g., pharmacist, pharmacist-designee, nurse or physician trained in aseptic handling techniques). This individual will be responsible for reconstitution, dilution and preparation of study drug (active and placebo) according to the randomization assignment for each subject. The investigational drug is to be administered only according to the conditions of this protocol. The individual designated to prepare the study drug may not participate in any study subject safety or efficacy evaluations.

Study drug will be administered by the study PI or his/her designees. Study drug should be allowed to come to room temperature prior to administration. The study drug contains no antibacterial preservatives and must be used within eight hours of reconstitution.

Study drug should be reconstituted with 1mL of PET diluent (resulting in 50µg bryostatin per mL or 0.0µg bryostatin per mL). After swirling the vial to completely dissolve the contents, the resulting solution must be diluted immediately with 9mL of 0.9% sodium chloride (NS) injection, United States Pharmacopeia (USP). The necessary volume of this solution to achieve the assigned dose for the subject should then be added to an IV infusion bag containing 50 mL of normal saline. Once the infusion bag is filled, the 45-minute infusion must be completed within 3 hours.

The infusion system (bag and tubing) should be made of a polyolefin plastic, such as polypropylene or polyethylene, or a combination of both. The use of polyvinylchloride (PVC) plastic bags and tubing is NOT recommended, as plasticizer from the PVC is leached and there is some limited absorption of bryostatin to PVC plastics.

The prepared infusion bag containing study drug should be labeled with the following information:

- Protocol number
- Kit/Subject number
- Date and time prepared
- Instructions for dosing (i.e., as a 45 minute (±5 min) IV infusion)

5.4 Study Drug Accountability and Disposal

Neither the Investigational Pharmacy, designated drug preparer, the PI nor any of his/her designees may provide drug to any person not enrolled in this study. Adequate records of study drug receipt and use must be maintained in order to comply with governmental regulations and with the protocol in addition to preventing unauthorized distribution.

Study drug orders, records of receipts, dispensing records, and inventory forms will be examined and reconciled throughout the study. All study drug that is used during the course of the study must be accounted for on a drug accountability form.

Unless otherwise directed, at the end of the study all unused study medication must be destroyed onsite after drug accountability has been verified by the monitor. If destruction on site is not possible, study medication should be retained and returned to BioConvergence at the end of the study. A copy of all completed drug accountability forms will be collected by the monitor or appropriate designee upon completion of the study.

Note: The medications should not be disposed of prior to monitoring and approval.

5.5 Randomization

Once all eligibility criteria for the study have been met, the subject will be randomized via IwRS. Instructions for use of the IwRS system are provided in the IwRS Manual. A randomization number will be assigned, as well as the concentration (20µg or 40µg) of study drug to be administered, and drug for that randomization number will be shipped to the site for twelve weeks of treatment. Randomization and scheduling of the first study drug infusion should be timed to allow for receipt of study drug prior to the scheduled study treatment. The drug kits will be shipped to the unblinded individual who will be responsible for kit storage and drug preparation and may not be handled by any other study staff

member. The study drug kit will not identify the vial containing study drug as either placebo or bryostatin.

5.6 Blinding

All subjects, PIs, and investigational clinical site personnel, with the exception of the individual responsible for study drug preparation, will be blinded to dose assignment. The individual responsible for preparation of study drug for infusion is not permitted to perform any safety or efficacy assessments and must maintain confidentiality of all dose assignments.

Since there is no known antidote to bryostatin, the blind should only be broken in exceptional circumstances and is at the discretion of the PI. The Medical Monitor should be contacted as soon as possible to discuss the situation but this should not delay any treatment.

In a non-emergency situation, when unblinding is requested, the site should discuss the clinical circumstances with the Medical Monitor to determine if breaking the blind will alter the subject's treatment. The decision to break the blind is ultimately the decision of the PI. If the blind is broken for a subject, the PI will record the date and reason for breaking the blind in the electronic case report form (eCRF) and study drug treatment will be discontinued. However, the subject will continue to be monitored per protocol for safety and efficacy.

5.7 Study Drug Administration

Study drug is scheduled to be administered by intravenous infusion only, via a pump, at the same rate over 45 minutes (±5 min) once a week for 2 weeks and then every 14 days ±2 days thereafter (See Figure 1 Study Design). The entire volume of study drug in the infusion bag is to be administered. Infusion times should not be extended or shortened. If a subject misses a dose, the dose should be administered as soon as possible. The subject should then continue on the original dosing schedule. Provided all efficacy assessments have been completed, if deemed necessary by the PI, a short acting sedative may be administered to reduce a subject's anxiety or agitation during the infusion. All medication is to be recorded on the Concomitant Medication eCRF.

6 STUDY PROCEDURES AND ASSESSMENTS

Subjects will be randomized to study drug, either bryostatin or placebo treatment. A final follow-up visit will take place 30 days after the last dose of study drug for all subjects, including subjects that have discontinued treatment before completion of the study. Assessments will be performed according to the Schedule of Activities (Table 1).

6.0 Table 1 Schedule of Activities

Week	Screening and Randomization		0	1	2	3	5	7	9	11	13	30-day Follow-up/ET
Day (±2 days)	(Days -28 to -2)		0	7	14	21	35	49	63	77	91	
Dose			1	2		3	4	5	6	7		
	Screening	Rand										
Informed Consent	X											
Medical history	X											
Demographics	X											
Neuroimaging	X[#]											
Rosen-modified Hachinski Scale	X											
SIB	X[Δ]						X*		X*		X	X
MMSE-2	X						X*		X*		X	X
ADCS-ADL-SIV[b]	X						X*		X*		X	X
NPI	X						X*		X*		X	
CGI-I							X*		X*		X	
CSSRS	X				X			X*			X	
Labs^^	X				X			X*			X	X
ECG	X (x3)				X			X			X	
PE	X*							X*+			X	X+
Vitals	X		X[c]	X[c]	X	X[c]	X[c]	X[c]	X[c]	X[c]	X	X
Randomization		X[a]										
Confirm Eligibility		X										
Study Drug Dosing			X	X		X	X	X	X	X		
PKCε			X^			X^				X^		
Bryostatin levels			X[∞]			X[∞]		X[∞]		X[∞]		
AE	X		X	X	X	X	X	X	X	X	X	X
Conmeds	X		X	X	X	X	X	X	X	X	X	X

[Δ]Baseline SIB administered during screening must be done within 3 weeks of the first dose of study drug. If the screening period exceeds 3 weeks, the SIB should be repeated on the day of first dose, prior to dosing.; *Before dose, ^PKCε samples prior to dose, at the end of infusion, and 60(±5) minutes after end of infusion (at selected sites); [∞]Bryostatin level samples prior to dosing, 15-20 minutes after start of infusion, at end of infusion (+5 minutes time window allowed), 15(±5) min, 30(±5)min, 45(±5) min, 60(±5) min and 120 (±10) min after end of infusion; # CT scan if imaging not performed within last 2 years or if subject has had significant worsening over last 12 months; ^^Labs: CBC including differential, coagulation, clinical chemistry, TSH, CPK at screening and event of myalgia, βhCG if indicated; B12, T-3 and T-4 if TSH abnormal, Hb A1C if clinically indicated; + abbreviated physical examination; [a] Randomization after initial screening procedures indicate eligibility; [b] The ADCS-ADL-SIV and NPI may be administered via telephone at the discretion of the investigator, within the allowed time window for the scheduled visit; [c] Vital signs (supine) prior to infusion, then at 30(±5), 60(±5) and 90(±5) minutes from start of the infusion, before and after drug administration.

6.1 Assessments

Every effort should be made to ensure that protocol-required tests and procedures are completed as described. However, it is anticipated that from time to time there may be circumstances outside the control of the PI that may make it unfeasible to perform a test. In these cases, the PI must take all steps necessary to ensure the safety and well-being of the subject. When a protocol required test cannot be performed, the PI will document the reason for the missed test and any corrective and preventative actions which were taken to ensure that the required processes are adhered to as soon as possible. The study team must be informed of these incidents in a timely manner.

6.1.1 Safety

Overall safety and tolerability will be assessed by the incidence of treatment emergent AEs and SAEs and by evaluations of change from baseline in physical examination, vital signs, 12-lead ECG, the C-SSRS, clinical chemistry, hematology, and coagulation lab tests.

6.1.1.1 Laboratory

Blood samples will be obtained for routine laboratory tests, including hematology, chemistry, coagulation and βhCG if indicated as outlined in the Schedule of Activities (Table 2).

Hematology tests will include CBC with differential, platelet count and coagulation (prothrombin time (PT) and partial prothrombin Time (PTT)) studies.

Clinical chemistry tests will include sodium, potassium, chloride, bicarbonate, blood urea nitrogen (BUN), creatinine, estimated creatinine CL, , glucose, calcium, CO2, total protein, albumin, ALP, ALT, AST, gamma glutamyl transferase (GGT) lactate dehydrogenase (LDH), uric acid and bilirubin. A serum creatine phosphokinase (CPK) will be done at screening and in the event of myalgia. A TSH will be done at screening, and T-3 and T-4 will be done if TSH result is abnormal.

Samples will be sent to a central lab for testing. Refer to the Laboratory Manual for detailed instructions.

6.1.1.2 Physical Examination

All physical examinations must be performed by the PI or qualified designee (physician, physician's assistant, or nurse practitioner). The complete physical examination conducted during the screening period and at designated subsequent time points should include, but is not limited to the following:

- General appearance
- Weight
- Height (screening only)
- Ears

- Eyes
- Nose
- Throat
- Neck
- Respiratory system
- Cardiovascular system
- Abdomen
- Musculoskeletal /Neurologic
- Extremities
- Skin
- Lymph nodes

An abbreviated PE will include but is not limited to the following:

- General appearance
- Weight
- Respiratory system
- Cardiovascular system
- Abdomen

Examination of other systems as needed to explore reports of AEs should be conducted as needed by the PI. Any clinically significant physical findings that were not present on the initial physical examination will be considered AEs and documented on the AE eCRF as well as in the subject's source documentation and on the physical examination eCRFs.

6.1.1.3 Vital Sign Measurements

Single supine blood pressure and pulse rate will be measured at Screening and at other visits as specified in the Schedule of Activities.

6.1.1.4 Electrocardiogram

An average of triplicate 12-lead ECGs will be collected at screening. The triplicate ECG measurements should be obtained approximately 2-4 minutes apart. Single 12-lead ECGs will be collected at time points specified in the Schedule of Activities.

6.1.1.5 Columbia Suicide Severity Rating Scale

The C-SSRS is an interview based rating scale to systematically assess suicidal ideation and suicidal behavior. The scale should be administered by an individual with appropriate clinical training, who has also taken the specific rater training for the scale, which will be provided by an agent of the Sponsor prior to the study start. If at any visit from baseline on, there are "YES" answers on items 4, 5 or on any behavioral question of the C-SSRS, a risk assessment should be done by a qualified clinician to determine whether it is safe for the subject to continue to participate in the study. A suicidality narrative should be constructed for subjects who have

undergone any post-baseline risk assessment, using information from the C-SSRS, and available information from the NPI, prior screening and baseline information, the clinician assessment and the narrative guide. Subjects who answer “YES” on items 4, 5 or on any behavioral question of the C-SSRS on more than one occasion during a study should be discontinued from the study. Suicidality AEs or other clinical observations may, based on the judgment of the PI, also trigger a risk assessment and a narrative. When there is a positive response to any question on the C-SSRS, the PI should determine whether an AE has occurred.

6.1.2 Efficacy / Psychometric Assessments

All raters for the efficacy assessments need to be trained and qualified on the administration of the scales. A qualified rater is required for the administration of the SIB, NPI and MMSE-2.

The same qualified rater should perform a given Efficacy/Psychometric Assessment at approximately the same time of day for a given subject throughout the study. All changes in raters for a given assessments must be noted in the subject’s source documents. The caregiver designated to provide assessments (ADCS-ADL-SIV and NPI) at the start of the study should be the same individual throughout the study. Should a change be necessary during the course of the study, the reason for the change and the first corresponding visit must be noted in the subject’s source document.

All psychometric tests performed on study subjects on the same day should be administered in the following order: SIB followed by MMSE-2. The tests should be administered prior to dosing.

6.1.2.1 Rosen-Modified Hachinski Scale

The Rosen-Modified Hachinski Scale will be evaluated at screening to differentiate Alzheimer’s type dementia from multi-infarct dementia. The 8-item scale results in a score of 0-12; and a score of 5 or above is exclusionary for the study.

6.1.2.2 Severe Impairment Battery

The SIB is used to assess cognition in subjects with moderate and severe AD. It is divided into nine subscales that include attention, language, orientation, memory, praxis, visuospatial ability, construction, social skills, orienting head to name. Non-verbal responses are allowed, thus decreasing the need for language output. Forty questions are included with a point score range of 0-100. Lower scores indicate greater cognitive impairment.

6.1.2.3 Mini Mental State Examination, 2nd Edition

The Standard version of the MMSE-2, with two alternate forms, will be used. This version has the structure and scoring of the original 30-item MMSE-2 and scores are comparable. The MMSE-2 is a brief, widely used test for assessing overall cognitive state. The MMSE-2 measures selected aspects of cognition such as memory, orientation, attention, language, and praxis on a scale of 0-30. Lower scores indicate greater cognitive impairment.

6.1.2.4 Alzheimer's Disease Cooperative Study Activities of Daily Living Inventory – Severe Impairment Version

The ADCS-ADL-SIV is a 19-item functional assessment of the performance of activities of daily living for subjects with moderate to severe Alzheimer's disease. Informants are queried via a structured interview format as to whether subjects attempted each item in the inventory during the previous 4 weeks, as well as their level of performance. Each item is rated from the highest level of independent performance to complete loss. Total score range from 0-54 with lower scores indicating greater functional impairment. The ADCS-ADL-Severe may be administered via telephone at the discretion of the PI, within the allowed time window for the scheduled visit.

6.1.2.5 Neuropsychiatric Inventory

The NPI is a caregiver interview-based rating scale assessing 12 behavioral disturbances occurring in dementia subjects. The NPI may be administered via telephone at the discretion of the investigator, within the allowed time window for the scheduled visit. Items are scored for both frequency and severity. Total scores range from 0-144 with higher scores indicating greater behavioral disturbances. For each item, the associated caregiver distress is also assessed.

6.1.2.6 Clinical Global Impression of Improvement Scale

The CGI-I is used to assess global change in the subject's condition compared to baseline before treatment. The same Clinician must complete the CGI-I scale at each scheduled visit. This is a seven-point scale ranging from (1) very much improved to (7) very much worse.

6.1.3 Pharmacokinetic and Pharmacodynamic Assessments

Selected sites will draw blood samples for pharmacokinetics at the time of doses 1, 3, 5, and 7. Samples will be drawn prior to dosing, at 15-20 minutes after the start of infusion, at the end of infusion (+5minute window), 15 minutes (±5minutes), 30 minutes (±5minutes), and 45(±5minutes) 60 minutes (±5minutes), and 120 minutes 120 (±10) after the end of infusion. If for any reason samples cannot be obtained for a dose as scheduled, they may be drawn at the time of the next scheduled dose.

In addition, selected sites will collect blood samples for assessment of PKCε concentration prior to the infusion, at the end of infusion (+5minute window) and 60 minutes after completion of infusion(±5minutes).) for doses 1, 3, and 7., respectively.

Instructions for sample preparation, handling and shipment are provided in the study Laboratory Manual.

6.2 Visit Procedures

6.2.1 Screening and Randomization (Days -28 to -2)

Informed Consent must be obtained before any study related procedures are performed. Screening procedures and randomization will take place within an approximate 4-week period prior to first dose administration (Days -28 to -2).

The following procedures should be performed first to avoid unnecessary procedures for ineligible subjects:

- Collect subject demographic data
- Review of medical history
- Assessment of neuroimaging (CT or MRI)
- Evaluate Rosen-Modified Hachinski Scale
- Evaluate MMSE-2

After confirming that these parameters satisfy eligibility criteria, the remaining screening procedures should be performed;

- SIB. The SIB should be done within 3 weeks before the first study drug dose. If the screening period exceeds 3 weeks, the SIB should be repeated on the day of the first dose, prior to dosing. The SIB score obtained closest to the day of dosing should be entered as the baseline value.
- NPI
- ADSC-ADL-SIV
- Columbia Suicide Severity Rating Scale (C-SSRS)
- Blood samples for routine safety including hematology, PT, PTT, INR and serum-chemistry, CPK, TSH (T-3 and T-4 if indicated), and β-hCG, if indicated
- 12-lead ECG (x3)
- Complete physical examination
- Vital signs
- AEs and concomitant medications

If changes in the subject's health or mental status occur during the screening and randomization period, including changes in medication affecting the mental status, the Medical Monitor should be notified. The Medical Monitor will advise the PI regarding any assessments that should be repeated to ensure eligibility requirements are met.

Sites will not be able to randomize subjects before confirmation that eligibility criteria have been met per the review of the Rosen-Modified Hachinski Scale, MMSE-2, and CSSRS by the Worldwide Clinical Trials' Clinical Assessment Technologies (CAT) group.

6.2.2 Week 0 (Day 0 Dose 1)

If the subject has had no intervening medical issues or changes in medications between the date of randomization and Week 0, dosing may proceed as scheduled.

A final assessment of the inclusion/exclusion criteria, including any changes in medications or health status will be done before dosing to confirm the subject's eligibility.

The following procedures will be done on Day 0:

- *Dosing by IV infusion* will be done according to the procedures outlined in Section 5.7.
- Vital signs prior to infusion, then at 30(±5), 60(±5) and 90(±5) minutes from start of the infusion
- At selected sites, blood sample to measure bryostatin levels (prior to dosing, 15-20 minutes after start of infusion, at end of infusion, 15, 30, 45, 60 minutes (±5 minutes, respectively) and 120 (±10) minutes after end of infusion. and PKCε assay (prior to dose, at the end of infusion, and 60 (±5) minutes after end of infusion) will be drawn and processed as described in the Laboratory Manual.
- AEs and concomitant medications

6.2.3 Week 1 (Day 7 (±2 days) Dose 2)

One week after the first dose of study drug, subjects will return for a second infusion. The following procedures will be performed:

- Vital signs prior to infusion, then at 30(±5), 60(±5) and 90(±5) minutes from start of the infusion
- *Dosing by IV infusion*
- AEs and concomitant medications

6.2.4 Week 2 (Day 14 (±2 days) No dose)

- C-SSRS
- Blood samples for routine safety
- 12-lead ECG
- Vital signs
- Adverse events and concomitant medications

6.2.5 Week3 (Day 21 (±2 days)/ Dose 3)

- Vital signs prior to infusion, then at 30(±5), 60(±5) and 90(±5) minutes from start of the infusion
- At selected sites, blood sample to measure bryostatin levels (prior to dosing, 15-20 minutes after start of infusion, at end of infusion, 15, 30, 45, 60 minutes (±5 minutes, respectively) and 120 (±10) minutes after end of infusion and PKCε assay (prior to dose, at the end of infusion, and 60 (±5) minutes after end of infusion) will be drawn and processed as described in the Laboratory Manual.
- *Dosing by IV infusion*

- Adverse events and concomitant medications

6.2.6 Week 5 (Day 35 (±2 days)/ Dose 4)

- Prior to dosing:
  - SIB
  - MMSE-2
  - ADCS-ADL-SIV
  - NPI
  - CGI-I
- Vital signs prior to infusion, then at 30(±5), 60(±5) and 90(±5) minutes from start of the infusion
- *Dosing by IV infusion*
- AEs and concomitant medications

6.2.7 Week 7 (Day 49 (±2 days)/ Dose 5)

- Prior to dosing:
  - C-SSRS
  - Blood samples for routine safety
  - 12-lead ECG
  - Abbreviated Physical exam
- Vital signs prior to infusion, then at 30(±5), 60(±5) and 90(±5) minutes from start of the infusion
- At selected sites, blood sample to measure bryostatin levels (prior to dosing, 15-20 minutes after start of infusion, at end of infusion, 15, 30, 45, 60 minutes (±5 minutes, respectively) and 120 (±10) minutes after end of infusion) as described in the Laboratory Manual
- *Dosing by IV infusion*
- AEs and concomitant medications

6.2.8 Week 9 (Day 63 (±2 days)/ Dose 6)

- Prior to dosing:
  - SIB

  - MMSE-2
  - ADCS-ADL-SIV
  - NPI
  - CGI-I
- Vital signs prior to infusion, then at 30(±5), 60(±5) and 90(±5) minutes from start of the infusion
- *Dosing by IV infusion*
- AEs and concomitant medications

6.2.9 Week 11 (Day 77(±2 days)/ Dose 7)

- Vital signs prior to infusion, then at 30(±5), 60(±5) and 90(±5) minutes from start of the infusion
- At selected sites, blood sample to measure bryostatin levels (prior to dosing, 15-20 minutes after start of infusion, at end of infusion, 15, 30, 45, 60 minutes (±5 minutes, respectively) and 120 (±10) minutes after end of infusion) and PKCε assay (prior to dose, at the end of infusion, and 60 (±5) minutes after end of infusion) will be drawn and processed as described in the Laboratory Manual.
- *Dosing by IV infusion*
- Adverse events and concomitant medications

6.2.10 Week 13 (Day 91 (±2 days)

At Week 13, subjects will undergo efficacy assessments as well as routine safety assessments. The following will be performed:

- SIB
- MMSE-2
- ADCS-ADL-SIV
- NPI
- CGI-I
- CSSRS
- Blood samples for routine safety
- 12-lead ECG
- PE

- Vital signs
- AEs
- Concomitant medications

6.2.11 30-day Follow-up Visit

For subjects who have completed or withdrawn from the study, a final follow-up visit will take place 30 days after the last dose of study drug. The following will be performed:

- SIB
- MMSE-2
- ADCS-ADL-SIV
- Blood samples for routine safety
- Abbreviated PE
- Vitals
- AEs and concomitant medications

6.3 Concomitant Medications

During the screening visit, the Caregiver will provide a history of prior medication use during the past 6 months and provide a list of currently used medications. All concomitant medications used should be recorded on the eCRF and in the source documents using the generic name for the drug. Assessment of concomitant medications will take place at each study visit. Any changes to chronic medications should be noted as well as new and discontinued medications.

Subjects taking allowed antidepressant medications may be enrolled in the study (see Appendix 1). The dose and dose regimen for these medications should be stabilized for at least 30 days prior to enrollment in the study. Every effort should be made to keep the dose and dose regimen of antidepressant medications stable throughout the study.

6.3.1 Medications for AD

Subjects taking FDA approved medications for the treatment of AD may be enrolled in the study. The subject must be on a stable dose for at least 3 months prior to entry into study and dose must not change during the study. Subjects may not initiate additional drugs for treatment of AD during the study other than the study drug.

6.3.2 Concomitant Medications for Management of Myalgia

Non-steroidal anti-inflammatory drugs such as ibuprofen and naproxen sodium are permitted. Use of acetaminophen is permitted, however, due to the theoretical consideration that it may inhibit PKC, it should be used sparingly.

6.3.3 Prohibited Medications

High dose Vitamin E (> 400 IU / day), Valproic Acid and divalproex sodium are prohibited.

If the PI determines that initiation of any of the prohibited medications is required to ensure the subject's safety, the medication may be initiated with the notification of the Medical Monitor and proper documentation of a protocol deviation. Other restricted concomitant medications are listed in Appendix 1.

7 ADVERSE EVENTS AND OTHER SAFETY EVALUATIONS

7.0 Definition of Adverse Events

An AE is any untoward medical occurrence associated with the use of a drug in humans, whether or not considered drug related. An AE (also referred to as an adverse experience) can be any unfavorable and unintended sign (e.g., clinically significant abnormal laboratory finding), symptom, or disease temporally associated with the use of a drug, without any judgment about causality.

The PI is responsible for obtaining information about all medical emergencies during the clinical study. The PI's contact information will be located in the body of the informed consent form (ICF) and subjects/caregivers will be encouraged to contact the PI or clinical site personnel during any clinical study-related emergency.

All adverse events spontaneously reported by the subject and/or caregiver in response to an open question or revealed by observation will be recorded during the study regardless of relationship to the study drug. All AEs will be monitored and recorded for the progress of the event until it resolves or reaches a clinically stable outcome. Adverse events that are not resolved at the time of database lock will be recorded as ongoing.

The AE and SAE reporting period starts from the time of consent to the last study visit.

7.1 Adverse Event of Special Interest - Myalgia

Myalgia has been reported as the dose limiting toxicity across the oncology studies. The incidence of myalgia appears to be dose dependent and cumulative. However, it has been reported in subjects receiving doses as low as 5µg/m^2. The myalgia has been investigated in some studies but not all. No increase in muscle enzymes were found in the cases investigated. EMG was abnormal in one subject who received 65µg/m^2 and suggested a patchy myositis, MRI in another subject was normal.

For all cases of myalgia despite the severity, a narrative will be created documenting onset, severity, treatment and outcome. Muscle enzymes, CPK, will be collected for all cases and compared to baseline values. Additional investigations are at the discretion of the investigator.

7.2 Definition of Serious Adverse Event

An SAE is any untoward medical occurrence that fulfills any of the following criteria:

- Results in death (fatal)
- Is life threatening (an event is considered "life threatening" if, in the view of either the investigator or sponsor, its occurrence placed the subject at immediate risk of death. It does not include an AE that, had it occurred in a more severe form, might have caused death).
- Requires in-patient hospitalization or prolongation of existing hospitalization
- Results in a persistent or significant disability/incapacity

- Is a congenital anomaly/birth defect

or

- Is an important medical event (events that may not result in death, be immediately life threatening or require hospitalization; may be considered serious when based upon appropriate medical judgment, they jeopardize the subject and may require medical or surgical intervention to prevent any of the outcomes listed above. Examples include; allergic bronchospasm requiring intensive treatment in an emergency room or at home, convulsions that do not result in hospitalization, or the development of drug dependency or drug abuse.

The following situations may not, by themselves, constitute sufficient grounds to be considered as an SAE:

1. Hospitalization solely for a diagnostic purpose, even if related to an AE,
2. Elective hospitalization for an intervention planned before the subject enrollment in the study
3. Admission to a day care facility or sleeping laboratory

7.3 Assessment of Intensity

Severity is a clinical determination of the intensity of an AE and will be determined by the PI based on the following classification criteria for all AEs occurring during the clinical study:

Mild - Awareness of signs or symptom, but easily tolerated, may require additional therapy

Moderate - Discomfort, enough to cause interference with usual activity and to require intervention or additional therapies

Severe - Incapacitating with inability to work or perform usual activity

Note: It should be noted that a severe AE need not be serious in nature and that an SAE is not, by definition, severe. Regardless of intensity, all SAEs and significant events must be reported.

7.4 Relationship to Study Drug

The causal relationship between the investigational drug and each AE will be determined by the PI based on his/her medical judgment in consideration of all relevant factors, including pattern of reaction, temporal relationship, positive, concomitant medication, co-existing diseases, and relevant medical history. The PI will classify every AE according to its relationship to study drug or trial-related procedures. The categories according to World Health Organization guidelines are listed in the following Table

7.5 Table 2 Relationship of AE to Study Drug or Trial-Related Procedures

Rating	Classification	Definition
1	Probable	An AE that:<br>• Occurs at a reasonable time interval after administration of the study drug;<br>• Follows a known response pattern to the study drug and;<br>• Cannot be reasonably explained by the known characteristics of the subject's clinical state or by other therapies.
2	Possible	An AE that:<br>• Occurs at a reasonable time interval after administration of the study drug;<br>• Follows a known response pattern to the study drug, but;<br>• Could have been produced by the subject's clinical state or by other therapies.
3	Unlikely*	• An AE for which sufficient information exists to indicate that the etiology is unrelated to the study drug;<br>• Another etiology is specified.

*If the AE is classified as unlikely, the PI should provide a likely cause, other illness, concomitant medication, or other.

7.5.1 Unexpected Adverse Event

An AE is considered "unexpected" if it is not listed in the Investigator's Brochure (IB) or is not listed at the specificity or severity that has been observed. Unexpected refers to AEs that are mentioned in the IB as occurring with a class of drugs or as anticipated from the pharmacological properties of the drug, but are not specifically mentioned as occurring with the particular drug under investigation. However, an event that is more specific or more severe than described in the IB will be considered unexpected.

7.6 Reporting Adverse Events

The PI should instruct all subjects/caregivers on the procedure for reporting AEs/SAEs to the appropriate clinical site personnel. For each AE reported by parents/guardians, clinical site personnel should obtain all required information to complete the eCRF.

The PI or designee should document all AEs/SAEs in subjects' source documentation and on the AE eCRF.

In addition to standardized reporting procedures, worsening or exacerbation of concurrent conditions in subjects will also be reported as AEs, and will follow the designated reporting format.

The following should be recorded with each SAE/AE:

- The nature of the AE, with a diagnosis wherever possible
- Date of event
- Assessment of intensity
- Is this an SAE?
- Relationship to study drug or trial-related procedures
- Action taken regarding study drug treatment
- Outcome

If the intensity of an already reported AE increases, then a new AE eCRF must be completed for that AE. The date of change would be included as the end date for the originally reported AE, and the start date for the new AE of greater intensity.

The clinical research associate is responsible for source document verification of all safety events.

If the clinical site becomes aware of an SAE, regardless of causality, within 30 days following the last administration of investigational product, the SAE should be recorded and reported immediately to the Sponsor. An SAE that occurs more than 30 days after the last dose will NOT be collected unless the PI considers that the event is related to the investigational product.

The sponsor should be informed if the PI becomes aware of any unusual safety information or any potential drug-related safety information, even after a subject completes the study

7.6.1 SAE reports

All SAEs, whether or not considered associated with study treatment or study-related procedures, must be reported on the eCRF immediately and no later than 24 hours after the site becomes aware of the event. Follow-up information must be provided promptly as requested.

The PI is obligated to provide as much information about the event as possible on the eCRF provided and as requested by the Sponsor or Medical Monitor.

In general, this will include a description of the SAE in sufficient detail to allow for a complete medical assessment of the case. The pharmacovigilance physician or designee, will review the SAE including the SAE criteria, the relationship to study medication, the expected or unexpected assessment, and inform the Sponsor by phone and e-mail immediately.

The following AEs should also be reported to the Sponsor’s designated Medical Monitor immediately:

- severe injection/infusion site reactions (ulceration or necrosis that is severe; operative intervention indicated)
- systemic hypersensitivity reactions
- myalgia

All additional follow-up evaluations must be reported by the site to the medical monitor, or designee, immediately after notification of the additional information.

An SAE will be followed until it resolves or reaches a clinically stable outcome. AEs/SAEs that have not resolved by study closure will be considered ongoing.

An SAE that occurs after the 30-day safety visit will NOT be collected unless the PI determines that the event is related to the investigational drug product.

7.6.2 Reporting to Regulatory Authorities

The PI, or designee, is responsible for informing the Institutional Review Board (IRB) of any unexpected SAEs, as well as any additional SAEs according to the IRB's policy.

Any SAE that is serious, suspected to be related to investigational study drug and unexpected (SUSAR), will be promptly reported to regulatory authorities by the Sponsor according to expedited reporting requirements. Subsequent relevant information after the initial submission of the (IND) Safety Report to the regulatory authorities will be submitted in a follow-up IND Safety Report to the regulatory authorities in the expedited period by the Sponsor.

7.7 Criteria for Withdrawal of Subjects

Subjects may be withdrawn from the clinical study for the following reasons:

- The PI believes withdrawal to be medically necessary or in the best interest of the subject
- Noncompliance with the protocol as judged by the PI (requires discussion with the Sponsor)
- Subjects who are enrolled in violation of inclusion and/or exclusion criteria
- An AE that presents an unacceptable consequence or risk to the subject as judged by the PI, Sponsor or the Medical Monitor
- Lost to follow up
- Withdrawal of consent
- Subject is unblinded

Subjects may voluntarily discontinue their participation in the study at any time without prejudice to further treatment.

7.8 Criteria for Permanent Discontinuation of Study Drug

Study drug treatment may be discontinued for the following reasons:

- if sponsor or regulatory authorities discontinue study
- if the PI believes that discontinuing treatment is in the best interest of the subject

7.9 Study discontinuation

Neurotrope BioScience, Inc. has the right to discontinue this clinical study at any time. The PI has the right to discontinue participation in this clinical study at any time for any reason. Clinical study site discontinuation should only occur after mutual consultation between the PI and Neurotrope BioScience, Inc.

Should the clinical study be discontinued prematurely, all subjects should be brought in for early termination procedures as outlined for the 30-day Follow-up visit. All clinical study materials should be returned to Neurotrope Bioscience, Inc. or designee.

8 INDEPENDENT DATA AND SAFETY MONITORING BOARD (DSMB)

Since there is limited exposure in this population and since the study population is largely elderly and potentially fragile, an independent safety monitoring board will be formed. The roles and responsibilities of the DSMB will be detailed in the DSMB Charter. The DSMB will have access to unblinded safety data and will conduct review of these data at regular intervals during the study. The DSMB may also conduct ad hoc safety reviews at their own request or at the request of the Sponsor.

The first safety analysis will be performed when 30 subjects have received four doses of study drug, followed by additional safety analyses when 60 and 90 subjects have received 4 doses. The safety analyses will be performed by an independent unblinded statistician. Tables and listings will be provided according to the procedures specified in the DSMB Charter. The DSMB will advise the sponsor of any safety issues. Any recommendations made by the DSMB to alter the conduct of the study will be forwarded to the Sponsor for appropriate action.

9 DATA ANALYSIS / STATISTICAL METHODS

Detailed methodology for summary and statistical analyses of the data collected in this study will be documented in a Statistical Analysis Plan (SAP). This separate document will be finalized prior to study unblinding and conduct of any statistical analyses. The SAP may modify and will take precedence over the plans outlined in the protocol; however, any major modifications or modification of the primary endpoint definition and/or its analysis will also be reflected in a protocol amendment.

Descriptive summaries of safety and efficacy data including number of evaluable subjects, mean, median, SD, maximum and minimum for continuous variables will be provided by treatment group and scheduled visit. Categorical variables will be presented showing number evaluable, frequencies and percentages. In addition, the same descriptive statistics will be provided for changes from baseline at each post-baseline visit.

The primary efficacy analyses of the primary and secondary endpoints will be conducted on data collected through Week 13.

9.0 Sample Size Determination

Since the primary objective of the study is to obtain safety and tolerability data for the administration of multiple doses of bryostatin, the planned sample size is based largely on feasibility. However, the following is an assessment of power for the primary analyses of the primary efficacy endpoint, estimation of the effect of bryostatin on the mean change from baseline in the SIB at Week 13.

It is estimated that 150 subjects equally randomized among the three dosing groups (two doses of bryostatin and placebo) will provide at least 80% power, assuming a true mean difference of at least 6.5 points in favor of the bryostatin groups (standardized effect size of 0.47), in a test between each bryostatin group and placebo in mean change from baseline in the SIB, (one-sided at α=0.10). This assumes the standard deviation of change from baseline at Week 13 is 14 points. This estimate allows that up to 15% of randomized subjects will not provide information due to discontinuation. The least significant difference for the test between the observed means under these conditions is estimated to be 3.9 points (standardized effect size of 0.28). Analysis Sets (Populations)

The Safety Analysis Set (SA) is defined as all randomized subjects who received any study medication (either partial or completed infusions of bryostatin or placebo).

The Full Analysis Set (FAS) used for efficacy analyses, consistent with the intention-to-treat (ITT) principles, is defined as all randomized subjects who received at least one dose of study medication and who have at least one post-baseline efficacy assessment.

9.1 Demographics, Baseline Characteristics and Disposition

Demographics will include age, sex, race, ethnicity, height, and body weight and will be summarized by treatment group using descriptive statistics for both the SA and FAS populations.

Medical history, neuroimaging, prior and concomitant medications, existing disease, years since AD onset and diagnosis, baseline medical conditions, and baseline safety and neuropsychological assessments will be summarized descriptively by treatment group for both the SA and FAS populations.

Subject disposition will be summarized by treatment group and will include numbers screened, randomized, dosed and withdrawn with reason for withdrawal.

9.2 Safety Analysis

The SA population will be used for all safety analyses with treatment group determined by the treatment actually received. AEs, safety laboratory, ECGs, physical exam and vital signs data will be presented in tabular format and summarized descriptively by treatment group. No formal hypothesis testing will be carried out on these safety assessments. Incidence and severity of AEs will be summarized by treatment dose using System Organ Class (SOC). AEs will be coded according to standard MedDRA terms.

C-SSRS responses will be mapped onto the Columbia-Classification Algorithm of Suicide Assessment (C-CASA) scale, and the frequency distribution of the C-CASA scores will be tabulated by treatment group and study visit. No hypotheses associated with the C-SSRS or C-CASA scales will be tested.

9.3 Efficacy Analysis

The FAS population will be used for efficacy analyses. In further keeping with ITT principles, analyses will be conducted according to randomized groups regardless of treatment actually received. Analyses will be performed on each dosing group vs. placebo and on the “pooled” dosing group vs. placebo.

Descriptive statistics by visit and randomized treatment group will be provided for all efficacy data. Efficacy evaluations will include inferential analyses and estimation at each visit for the change from baseline in the SIB (primary endpoint), ADCS-ADL-Severe, MMSE-2 and NPI, and the value at each visit for the CGI-I. The primary analysis will test the hypothesis that there is a beneficial effect of each bryostatin dose group or the combined (pooled) bryostatin group compared to placebo on the change from baseline in the SIB at the Week 13 visit. The secondary efficacy endpoints will be analyzed in all 3 groups (i.e., each dose separately and pooled vs. placebo); p-values and confidence intervals will be reported and used to assist in interpretations of the results.

All statistical tests will be one-sided and use $\alpha = 0.10$ unless otherwise stated. LSM and two-sided 80% confidence intervals will be provided for treatment group differences and estimated endpoint values by visit. No multiplicity adjustments will be made. Estimates of effect with confidence intervals and p-values comparing each dose of bryostatin to placebo and the pooled bryostatin group to placebo will be provided for all endpoints.

9.3.1 Analysis of Primary Endpoint

The primary statistical objective for efficacy is to estimate the effect of bryostatin on the mean change in the SIB at Week 13. A linear model will be used for both estimation and significance testing for the primary endpoint. This will be a Mixed Model for Repeated Measures (MMRM) with fixed effects for treatment, baseline MMSE-2 stratum, scheduled visit (nominal) and scheduled visit by treatment interaction, plus a random effect for subject. The baseline SIB measurement will be a covariate. The primary contrast will be the change from baseline in the SIB at Week 13 between each bryostatin group and the placebo group. Inferences will be based on the model estimates of expected values for the treatment by visit interaction.

Secondary analyses of the primary endpoint will include analyses at the other scheduled visits (Weeks 5 and 9).

9.3.2 Analysis of Secondary Endpoints

The ADCS-ADL-SIV, MMSE-2, and NPI secondary endpoints will be analyzed using the statistical model used in the analysis of the SIB. The model for the CGI-I will differ in that there is no baseline value used as a covariate in this global assess of change. These analyses will use the statistical models to generate point estimates, confidence intervals and p-values and will be

used to support the results of the primary endpoint analysis in addressing the primary efficacy objective.

9.4 Pharmacokinetics

Plasma samples will be analyzed for bryostatin concentrations using a validated assay. A separate Pharmacokinetic Analysis Plan (PAP) will be developed prior to the pharmacokinetic analysis that will describe methods to be used, as well as the tables, figures, and listings that will be generated as part of this analysis.

Plasma samples may be analyzed to determine PKCε amount in PBMCs. Dose-response relationships may be examined, as well as possible exposure-response relationships.

9.5 DSMB Safety Assessments

Unblinded summaries and listings of safety data will be provided to the Independent DSMB as described in Section 7. Details for these will be described further in the DSMB Charter and the Interim Analysis Plan (IAP). Analyses will be performed by an independent unblinded statistician so that study investigators and other sponsor personal (and contractors) involved in study monitoring, data processing and other aspect of the study remain blinded.

10 DATA MONITORING

10.0 Source Documentation

In accordance with ICH-GCP guidelines, source documents may include, but are not limited to the following:

- Clinic, office, hospital charts
- Copies of transcribed health care provider notes, which have been certified for accuracy after production
- Recorded data from automated instruments such as x-rays and other imaging reports, sonograms, computed axial tomography scans, magnetic resonance images, radioactive images, electrocardiograms, electroencephalograms
- Records of telephone contacts
- Diaries, evaluation checklists, or questionnaires that are completed directly by subjects or caregivers and serve as their own source
- Laboratory results and other laboratory test results, urine dip-stick results
- Correspondence regarding a subjects’ treatment between physicians or memoranda sent to the IRB.

10.1 Study Documentation and Record Retention

The PI, or designees, must enter all results collected during the clinical study into eCRFs. eCRF completion guidelines will be reviewed with clinical site personnel at the PI's Meeting and site initiation visits. PIs are responsible for approval of all entered or corrected data. The PI, or designees, must review and approve the data before database lock, or before any scheduled interim analyses, as required by the sponsor.

The medical records (source documents) upon which the eCRFs are based must be kept at the clinical site for at least a period of 2 years following the date a marketing application is approved for the drug for the indication for which it is being investigated; or, if no application is to be filed or if the application is not approved for such indication, until 2 years after the investigation is discontinued and the FDA is notified. Neurotrope BioScience, Inc. must be informed if the records are passed on to any other person or institution during this period. Records related to the study will be maintained by Neurotrope BioScience, Inc. or its designee for a minimum of 5 years per 21 Code of Federal Regulations (CFR) 58.195 (b) 2.

10.2 Site Monitoring

The Sponsor or its designee will be allowed to conduct site visits at the investigation facilities to monitor any aspect of the study. The PI will provide Neurotrope BioScience, Inc., or its designee, with documentation of IRB approval of the Study Protocol and the Informed Consent prior to study initiation and IRB approval of any subsequent amendments to the protocol or revision to the Informed Consent. Before a study site can enter a subject into the study, the Sponsor or a designee will visit the study site to:

- Determine the adequacy of the facilities
- Discuss with the PI(s) and other personnel their responsibilities with regard to protocol adherence, and the responsibilities of Neurotrope BioScience, Inc., or its representatives. This will be documented in a clinical study agreement between Neurotrope BioScience, Inc. and the PI.

During the study, a monitor from Neurotrope BioScience, Inc., or representative will have regular contacts with the investigational site, for the following:

- Provide information and support to the PI(s)
- Confirm that facilities remain acceptable
- Confirm that the investigational team is adhering to the protocol, that data are being accurately recorded in the eCRFs, and that investigational product accountability checks are being performed
- Perform source data verification. This includes a comparison of the data in the eCRFs with the subject's medical records at the hospital or practice, and other records relevant to the study. This will require direct access to all original records for each subject (e.g. clinic charts).

- Record and report any protocol deviations not previously sent to Neurotrope BioScience, Inc
- Confirm AEs and SAEs have been properly documented on CRFs and confirm any SAEs have been reported and those SAEs that met criteria for reporting have been forwarded to the EC/IRB/ Independent Ethics Committee (IEC).

The monitor will be available between visits if the PI(s) or other staff needs information or advice.

10.3 Quality Assurance and Quality Control

To ensure compliance with GCP and all applicable regulatory requirements, Neurotrope BioScience, Inc. or its representative may conduct a quality assurance audit.

10.4 Audits and Inspections

Authorized representatives of, Neurotrope Bioscience, Inc., a regulatory authority or an IRB may visit the site to perform audits or inspections, including source data verification. The purpose of an audit or inspection is to systematically and independently examine all study-related activities and documents to determine whether these activities were conducted, and data were recorded, analyzed, and accurately reported according to the protocol, GCP guidelines of the International Conference on Harmonization, and any applicable regulatory requirements. The PI should contact Neurotrope BioScience, Inc. immediately if contacted by a regulatory agency about an inspection.

11 ETHICS

11.0 Institutional Review Board

The PI must obtain IRB approval before initiating any study activities.

The final study protocol, including the final version of the Informed Consent Form, must be approved in writing by an IRB. The PI must submit written approval to Neurotrope BioScience, Inc. or its representative, before he or she can enroll any participant into the study.

The PI is responsible for informing the IRB of any amendment to the protocol in accordance with local requirements. In addition, the IRB must approve all advertising used to recruit subjects for the study. The protocol must be re-approved by the IRB upon receipt of amendments and annually, as local regulations require.

The PI is also responsible for providing the IRB with reports of any reportable serious adverse drug reactions from this or any other study conducted with the investigational product. Neurotrope BioScience, Inc. will provide this information to the PI.

In addition to SAEs that are suspected unexpected serious adverse reactions (SUSARs), the PI or designee will report any additional SAEs that may be required according to the IRB policy.

Progress reports will be provided to the IRB according to local regulations and guidelines.

11.1 Ethical Conduct of the Study

The conduct of this study will be consistent with ICH Guidance E6, GCP, and U.S. federal regulatory requirements, as applicable. This study will be conducted in accordance with applicable local law(s) and regulation(s) and the principles of protection of healthy human subjects participating in clinical medical research that have their origin in the Declaration of Helsinki. The PI must agree to the direct access to source documents and inspection of clinical study-related records by the regulatory authority/Neurotrope BioScience representatives.

11.2 Written Informed Consent

Written informed consent must be obtained from caregiver and subject (if possible) or legally acceptable representative if different from caregiver.

The PI should confirm to the extent possible that the subject has the necessary caregiver support and will be able to attend scheduled study visits for the duration of the study.

Before starting the clinical study, the PI must have the IRB's written approval or favorable opinion of the written ICF and any other written information to be provided to parents or guardians of subjects. The written approval of the IRB together with the approved subject's information/ICF must be in the clinical study files. The process of obtaining informed consent must be in accordance with applicable regulatory requirements and must adhere to ICH E6(R1) guidelines and the ethical principles in the Declaration of Helsinki. Written informed consent must be obtained and documented before any clinical study-specific procedure takes place. Participation in the clinical study and dates of informed consent given by subjects should be documented in the subjects' files.

12 STUDY MANAGEMENT

12.0 Data Collection and management

For all written documentation such as source documents, the data collected during the study must be legibly printed using a permanent ink pen. A single line should be drawn through any incorrect information. Opaque correction fluids or tapes are not permitted. All corrections or deletions to any of the source documents must be dated and initialed. All corrections or deletions to the eCRF will be documented via an electronic audit trail. The PI or designee will electronically sign each subject's final eCRF to signify that all of the information is correct and complete.

12.1 Data Quality Control

Periodic on-site review of communications between the PI and investigational site study monitors, and review of eCRF data and source documents are the responsibility of the Sponsor,

or designee. The eCRF data for each subject will be reviewed against source documents at the study sites by the investigational site study monitor.

The PI and investigational site will allow study related quality control monitoring and audits, EC/IRB/IEC review, and/or regulatory inspection and will cooperate in providing direct access to source data and documentation.

12.2 Data Management and Data Storage

Study procedures will be documented on source documents that will be retained at the site(s). An Electronic Data Capture (EDC) system will produce eCRFs that will be used to collect assessment data for this study. All study data entered into the eCRF will be compliant with regulatory requirements and 21CFR part 11. The system will allow differing levels of access and will accommodate roles for the PI, Medical Monitor, CRO, and Sponsor. All data changes made within the system will be subjected to an audit trail. In compliance with GCP, source documentation supporting the eCRF data should indicate the subject's participation in the study and should clearly document the dates and details of study procedures, AEs, and subject status. The eCRFs will identify study subjects with unique identifiers. Data are recorded from the source documents, directly onto the eCRF at the site.

Electronic CRF data items will undergo quality control standards of operation. Unresolved errors, omissions, or requests for clarification will trigger a query to the Investigational Site for resolution via electronic queries. The database will be corrected for completeness and accuracy. Prior and concomitant medications will be entered into the eCRF and coded using the World Health Organization Drug Reference List (WHO-DRL). Medical history, concurrent medical conditions, and AEs will be coded using MedDRA.

A quality assurance audit will be conducted to verify the accuracy and completeness of the database and will be done prior to declaring database lock. The database will not be altered after lock, unless joint written agreement is obtained between the CRO and the Sponsor.

12.3 Inspection of Records

Neurotrope BioScience, Inc., will be allowed to conduct site visits to the investigation facilities for the purpose of monitoring any aspect of the study. The PI agrees to allow the monitor to inspect the drug storage area, study drug stock, drug accountability records, subject charts and study source documents, and other records relative to study conduct.

12.4 Retention of Records

A searchable offline version of the eCRF will be forwarded to the Sponsor for storage. A copy of each completed eCRF will remain in the PI's study file on a compact disk. All source documentation, eCRFs and administrative records will be retained by the PI for a minimum of 2 years following agency approval of the medication for the indication under study or following notification that the investigational application is closed for this indication. However, this may be adjusted based on the applicable local requirements. After this time, the documentation will either be destroyed or transferred to Neurotrope BioScience, Inc., or designee. No study

documentation should be destroyed or moved to a new location without prior written approval by Neurotrope BioScience, Inc.

12.5 Confidentiality

All study findings and documents will be regarded as confidential. The PIs and members of their research teams must not disclose such information without prior written approval from Neurotrope BioScience, Inc. or its representatives.

The anonymity of participating subjects must be maintained. A Protected Health Information statement will be provided to each subject either as a part of the Informed Consent document or as a separate form. Subjects will be identified on eCRFs and other documents by their initials, birth date, and subject number. Documents that identify the subject by name (eg, the signed Informed Consent Form) must be maintained in strict confidence by the PIs.

12.6 Protocol Amendments

Minimally, any change that significantly affects the safety of subjects, the scope of the investigation, or the scientific quality of the study will be effected by means of a protocol amendment approved by the Sponsor. Any changes that affect subject safety or welfare must be submitted to the relevant IRB and approved before implementation.

The PI will provide written agreement of the protocol amendment via the approval signature page. The PI will notify the IRB of the amendment and obtain approval prior to implementation. If the change is intended to eliminate an immediate hazard, the amendment will be implemented immediately, prior to IRB notification.

12.7 Protocol Deviations

Should a deviation from the protocol be deemed crucial for the safety and well-being of a particular subject, such a deviation will be instituted for that subject only. The PI or other attending physician should contact the Medical Monitor as soon as possible. In addition, the PI or designee should document in the source document the reasons for the protocol deviation and the ensuing events. No protocol deviations for any Inclusion or Exclusion criterion will be permitted in this study.

12.8 Data Corrections

For all written documentation such as source documents, the data collected during the study must be legibly printed using a permanent ink pen. A single line should be drawn through any incorrect information. Opaque correction fluids or tapes are not permitted. All corrections or deletions to any of the source documents must be dated and initialed. All corrections or deletions to the eCRF will be documented via an electronic audit trail. The PI will electronically sign each subject's final eCRF to signify that all of the information is correct and complete.

12.9 Insurance

The Sponsor has taken out a liability insurance policy, which covers the liability of PIs. This policy is in accordance with local laws and requirements.

The Sponsor's insurance does not relieve the PI or the collaborators of any obligation to maintain their own liability insurance policy as required by the applicable law.

13 PUBLICATION AND DISCLOSURE POLICY

Study findings are an integral part of the overall commercialization plan for this investigational compound. To this end, the contents of this protocol and any amendments and results obtained during the study shall be kept confidential by the investigator, the investigator's staff, and the IRB/IEC, and shall not be disclosed in whole or in part to others, or used for any purpose other than reviewing or performing the study, without the review and prior written consent of the Sponsor. These obligations of confidentiality and non-use shall in no way diminish such obligations as set forth in either the Confidentiality Agreement or Clinical Trial Agreement executed between the Sponsor/CRO and the institution/investigator. All persons assisting in the performance of this study must be bound by the obligations of confidentiality and non-use set forth in either the Confidentiality Agreement or Clinical Trial Agreement executed between the institution/investigator and the Sponsor/CRO. Additionally, the publication plan of the Sponsor, considering, among other items, proprietary patent issues and competitive strategic goals, must be complied with prior to the public disclosure of any aspect of this study by abstract, verbal presentation, invited lecture, journal article, or journal letter. Matters regarding authorship and the order of authorship on publications reporting the results of single study findings are covered in a separate agreement.

14 APPENDIX 1 - RESTRICTED CONCOMITANT MEDICATIONS

Drugs Allowed (Y) and Not Allowed (N) as Concomitant medications in this study.

Drug Class	Usage (p.r.n.)	Chronic Use	Restrictions
Analgesics	(Y)	(Y)	Only non-opioid containing analgesics can be administered chronically. Use of acetaminophen is permitted, however, due to the possibility that it may inhibit PKC, it should be used sparingly. Combination products containing codeine, hydrocodone or oxycodone may be used on a p.r.n. basis only (not to exceed 5 consecutive days) and not within 24-hours of a clinic visit.
Anesthetics<br>• *General*<br>• *Local*	 (N)<br>(Y)	 (N)<br>(N)	
Anorexics	(N)	(N)	
Antacids	(Y)	(Y)	
Antianginal agents	(Y)	(Y)	
Antiarrhythmics	(N)	(Y)	Dose must be stable for 1 month prior to Screening.
Antiasthma agents	(Y)	(Y)	
Antibiotics	(Y)	Call	Call Medical Monitor.
Anticholinergics	(N)	(N)	Includes Cogentin. Anticholinergics for bladder control are to be avoided if possible. When needed some, such as Detrol is allowed. Call Medical Monitor.
Anticoagulants	(N)	(Y)	Heparin is not allowed
Anticonvulsants	(N)	(N)	Divalproex, Valproate, Topiramate are prohibited. Anticonvulsants with no known significant cognitive effects, such as Lamictal, Pregabalin, Levetiracetam are allowed. Call

Drugs Allowed (Y) and Not Allowed (N) as Concomitant medications in this study.

Drug Class	Usage (p.r.n.)	Chronic Use	Restrictions
			the medical monitor for other anticonvulsants. Patient must have been on a stable dosed for 3 months prior to screening.
Antidepressants	(N)	(Y)	MAO inhibitors, antidepressants with anticholinergic effects (e.g. tricyclics), and chronic use of sedating antidepressants (e.g. mirtazipine) are not allowed. Sedating antidepressants can be used sparingly, as needed for sleep; avoid using 12 hrs prior to efficacy assessments. Dose and medication must be stable for 1 month prior to Screening.
Antidiarrheal preparations	(Y)	(N)	Kaolin, Imodium and Pepto-Bismol preparations are allowed. Call Medical Monitor for others.
Antiemetics	(Y)	(N)	Antiemetics with sedative properties (e.g. first generation antihistaminics) are not allowed. Antiemetics such as phosphoric acid preparations (Emetrol, Emecheck), Pepto-Bismol, and cola syrup are allowed.
Antifungal agents<br>• *Systemic*<br>• *Topical*	 (N)<br>(Y)	 (N)<br>(Y)	
Antihistamines	(Y)	Call	Non-sedating antihistaminics such as Allegra (fexofenadine), Zyrtec (cetirizine) etc are allowed. Sedating antihistaminics are not allowed. See cough and cold preparations for combination products.
Antihypertensives	(N)	(Y)	Diupres (reserpine), Catapres (clonidine), Minipress (prazosin), Inderal (propranalol), Wytensin (guanabenz), Tenex (guanfacine) and Aldomet (methyldopa) are not allowed. All others - medication

Drugs Allowed (Y) and Not Allowed (N) as Concomitant medications in this study.

**Drug Class**	**Usage** (p.r.n.)	**Chronic Use**	**Restrictions**
			and dose must be stable for 1 months prior to Screening.
Anti-inflammatory drugs	(Y)	(Y)	Indomethacin and systemic corticosteroids are not allowed.
Anti-neoplastics	(N)	(Y)	Tamoxifen is allowed. Dose must be stable for 3 months prior to Screening.
Anti-obesity	(N)	(Y)	Xenical (orlistat) Cetilistat, Lorcaserin (Belviq) are allowed. Others such as Qsymia (a combination of phenermine and topiramate) are not allowed. Call Med Monitor. Dose must be stable for at least one month prior to Screening.
Anti-Parkinson's drugs	(N)	(N)	Includes dopaminergic agents, amantadine, selegiline, Cogentin, and MAO inhibitors.
Antipsychotics	(Y)	(Y)	Clozapine and antipsychotics with anticholinergic effects are not allowed. Limit use to low doses and only if absolutely needed. Call Medical Monitor. Doses must be stable for at least *one month* prior to Screening.
Antiviral agents	(Y)	(Call)	Zovirax, Valtrex, Famvir are allowed. For others, call Medical Monitor
Anxiolytics	(Y)	(N)	Limited use of short/medium acting benzodiazepines if needed is allowe Do not use within 12 hrs before efficacy assessments.
BPH agents	(N)	(Y)	Alpha-1 blockers (Hytrin, Flomax, Cardura) and finasteride are permitted. Drug and dose must be stable for 3 months prior to Screening.
Cholinesterase inhibitors	(N)	(Y)	Alzheimer's disease medications such as Aricept (donepezil) Exelon (rivastigmine) Reminyl (galantamine), and memantine

Drugs Allowed (Y) and Not Allowed (N) as Concomitant medications in this study.

**Drug Class**	**Usage**<br>**(p.r.n.)**	**Chronic Use**	**Restrictions**
			must be on a stable dose for at least 3 months prior to Screening.<br>Usage must continue unchanged throughout the study.
Cough/Cold preparations	(Y)	Call	Decongestants containing dextromethorphan or narcotics are not permitted. Preparations containing pseudoephedrine or phenylpropanolamine are not permitted. *See Antihistamines.*
Diuretics	(N)	(Y)	Medication and dose must be stable for 1 months prior to Screening.
Ginko biloba	(N)	(Y)	Dose must be stable for at least one month prior to Screening.
H_2 Blockers	(Y)	(Y)	For patients on chronic therapy, medication and dose must be stable for 1 months prior to Screening.
Hormones	(N)	(Y)	Medication and dose must be stable for 3 months prior to Screening.
Hormone suppressants	(N)	(Y)	Proscar (finasteride) is allowed. Dose must be stable for 3 months prior to Screening.
Hypoglycemic agents	(N)	(Y)	Oral hypoglycemic agents and insulin are allowed. Dose must be stable for 3 months prior to Screening.
Hypolipidemics	(N)	(Y)	Statins are allowed. Dose must be stable for at least one month prior to Screening.
Insulin	(N)	(Y)	Patients must be well controlled and stable.
Laxatives	(Y)	(Y)	Fiber-based products and Colace (docusate sodium) are allowed.
Muscle relaxants	(N)	(N)	
Psychotropic drugs not otherwise specified (including herbal products)	(N)	(N)	Call medical monitor

Drugs Allowed (Y) and Not Allowed (N) as Concomitant medications in this study.

**Drug Class**	**Usage**<br>**(p.r.n.)**	**Chronic Use**	**Restrictions**
Sedatives/hypnotics	(Y)	(Y)	Zolpidem, zaleplon, trazodone, chloral hydrate, mirtazapine, and the occasional use of benzodiazepines for sleep is allowed. Where possible, these should not be used within 12 hrs prior to efficacy tests.
Steroids<br>• *Systemic*<br>• *Topical*<br>• *Inhalant*	 (N)<br>(Y)<br>(Y)	 (N)<br>(Y)<br>(Y)	
Stimulants	(N)	(N)	Includes Ritalin, Concerta, any methylphenidate preparations, Cylert (pemoline), etc.
Tocopherol (Vitamin E)	(N)	(Y)	Dose more than 400 IU per day notallowed; Dose must be stable for at least one month prior to Screening.
Vaccines for AD	N	N	Patients treated with active vaccine against amyloid/tau within 2 years are not allowed.

15 APPENDIX 2 LIST OF REFERENCES

[1] McKhann GM, Knopman DS, Chertkow H, Hyman BT, Jack CR, Kawas CH, Phelps CH. 2011. The diagnosis of dementia due to Alzheimer's disease: recommendations from the National Institute on Aging-Alzheimer's Association workgroups on diagnostic guidelines for Alzheimer's disease. Alzheimer's & Dementia : The Journal of the Alzheimer's Association, 7:263–9.

[2] American Psychiatric Association. Diagnostic and Statistical Manual of Mental Disorders, 5th Edition: DSM-5 – May 27, 2013. American Psychiatric Association. Washington, D.C.

[3] http://www.heart.org/HEARTORG/Conditions/HeartFailure/AboutHeartFailure/Classes-of-Heart-Failure_UCM_306328_Article.jspList of References

[4] Nelson PT, Head F, Schmitt FA, e al. 2011. Alzheimer's disease is not "brain aging": neuropathological, genetic and epidemiological human studies. Acta Neuropathol. 121:571-87.

[5] Hongpaisan J and Alkon DL. 2007. A structural basis for enhancement of long-term associative memory in single dendritic spines regulated by PKC. PNAS. 104 (49):19571-19576l.

[6] Sun, M ad Alkon DL. 2014. The "memory kinases": roles of PKC isoforms in signal processing and memory formation. Progress in Molecular Biology and Translational Science. Elsevier Inc. Vol. 122

[7] Kern A, Roempp B, Prager K, Walter J, Behl C. 2006. Down-regulation of endogenous amyloid precursor protein processing due to cellular aging. J Biol Chem 281:2405-2413.

[8] Battaini F, Elkabes S, Bergamaschi S, Ladisa V, Lucchi L, De Graan PN, Schuurman T, Wetsel WC, Trabucchi M, Govoni S. 1995. Protein kinase C activity, translocation, and conventional isoforms in aging rat brain. Neurobiol Aging 16:137-148.

[9] Battaini F, Pascale A. 2005. Protein kinase C signal transduction regulation in physiological and pathological aging. Ann N Y Acad Sci 1057:177-192.

[10] Battaini F, Pascale A, Paoletti R, Govoni S. 1997. The role of anchoring protein RACK1 in PKC activation in the ageing rat brain. Trends Neurosci 20:410-415.

[11] Larbi A, Douziech N, Fortin C, Linteau A, Dupuis G, Fulop T, Jr. 2005. The role of the MAPK pathway alterations in GM-CSF modulated human neutrophil apoptosis with aging. Immun Ageing 2:6.

[12] Anderson JJ, Holtz G, Baskin PP, Wang R, Mazzarelli L, Wagner SL, Menzaghi F. 1999. Reduced cerebrospinal fluid levels of alpha-secretase-cleaved amyloid precursor protein in aged rats: correlation with spatial memory deficits. Neuroscience 93:1409-1420.

[13] Hongpaisan J, Sun M and Alkon DL. 2011. PKCε activation prevents synaptic loss, Aβ elevation, and cognitive deficits in Alzheimer's disease transgenic mice. The Journal of Neuroscience 32 (2):630-643.

---

[14] Schroot LM, Jackson K, Yi P et al. 2015. Acute oral Bryostatin-1 administration improves learning deficits in the APP/PS1 transgenic mouse model of Alzheimer's disease. Current Alzheimer Research 12: 222-31.

[15] Etcheberrigaray R, Tan M, Dewachter I et al. 2004. Therapeutic effects of PKC activation in Alzheimer's disease transgenic mice. PNAS: 101 (30): 11141-11146.

[16] Sperling RA, Jack CR, Black SE, Frosch MP, Greenberg SM, Hyman BT, Scheltens P, Carrillo MC, Thies Martin W, Bednar MM, Black RS, Brashear HR, Grundman M, Siemers ER, Feldman HH, Schindler RJ. 2011. Amyloid-related imaging abnormalities in amyloid-modifying therapeutic studies: recommendations from the Alzheimer's Association Research Roundtable Workgroup. Alzheimer's & dementia : 7(4), 367–85.

16 APPENDIX 3 – SUMMARY OF CHANGES

16.1 Version 2.0, 20 January 2016

Spelling, grammar and syntax corrections have been made throughout the document for clarity and correctness. In addition, the following changes and corrections were made.

Study Administrative Structure:

Study Medical Monitor/Emergency Contact Information has been updated to list Idil Cavus, MD PhD as medical monitor.

Central Laboratory has been changed to ACM Global

eResearchTechnology, Inc. (ERT) has been added as the central ECG vendor

Pharmacokinetic and Pharmacodynamic Plasma Sample Analysis Laboratory locations have been updated.

Protocol Synopsis and Section 4.4.2, Exclusion Criteria:

Exclusion 5 has been modified from poorly controlled diabetes, with a hemoglobin A1c of 8.5% or greater to poorly controlled diabetes, at the discretion of the Principal Investigator. This change was implemented to remove the perceived but unintended requirement to test hemoglobin A1c for all subjects during screening. Investigators may elect to test subjects suspected of having poorly controlled diabetes, but the test is intended to be done at the investigator's discretion based on medical history or clinical assessment.

Exclusion 6 has been modified from Creatinine CL of <60ml/min to Creatinine CL of <45ml/min. The rationale for this change is as follows:

The lower exclusionary CL of <45 mL/min is based on the National Kidney Foundation (NKF) guideline and recommendations, as well as on the extensive experience with bryostatin in oncology patients (absence of renal complications).

CL is measured using the Cockcroft-Gault (C-G) equation, which takes into account the age and gender of the patients. According to the National Kidney Foundation (NKF), almost 75% of individuals = or >70 years old may have GFR <90 mL/min/1.73 m2, and almost 25% may have GFR <60 mL/min/1.73 m2. (NKF Guideline pg 263).

Since single measures of GFR can fluctuate and is influenced by hydration, diet, medications, etc., the NKF in their recent US commentary recommends follow up of patients based on their renal function, together with other risk factors such as hypertension, diabetes etc. (https://www.kidney.org/professionals/guidelines/guidelines_commentaries). Therefore, for patients who have CL<60, the PI should refer patients to their primary care provider for appropriate follow-up.

The NKF endorses the new distinction between chronic kidney disease (CKD) stage 3a (GFR of 45-59 mL/min/1.73 m2) and 3b (GFR of 30-44 mL/min/1.73 m2) in the updated guideline. As reviewed in the guideline, the risks of mortality and other outcomes vary greatly between

these groups. Consistent with this, in their 2014 guideline severe dose restrictions of certain meds are recommended only for patients with CL <30.

Exclusion 7 has been corrected to exclude subjects using > 400 International Units of vitamin E. The previous version specified ≥ 400 International Units in some areas of the document, which was incorrect. A corresponding correction was made to Section 17, Appendix 1, Restricted Concomitant Medications.

Exclusion 17 has been modified to clarify if and when laboratory tests should be done to rule out alternate etiology for dementia. If the patient has prior history of serum B12 abnormality, anemia with hemoglobin ≤10g /dl, thyroid function abnormality, electrolyte abnormality, or positive syphilis serology the patient should be revaluated to determine if these potential causes of dementia have been addressed. Only if these causes have been ruled out as the cause of the dementia can the patient be enrolled.

Exclusion 18 has been clarified to define the established cut-off for QTc: History of prolonged QT or prolonged QT on screening ECG (QTcB or QTcF >499 per central reader).

The following text has been added to Section 4.4.2 regarding re-screening and expected follow-up:

"Subjects who are screen-failed (e.g. due to clinically significant laboratory abnormality or an active medical condition) may be re-screened if their medical condition stabilizes or improves as assessed by the PI. Subjects with CL<60ml/min should be referred for follow-up by their primary care provider." List of Abbreviations, and Sections 5.3, 5.6 and 8.2.10

The term Interactive Response Technology (IRT) has been replaced with Interactive Web Response System (IwRS), which is the specific system used in this study.

Section 2.1

Numbers of patients treated in oncology trials have been updated.

Section 5.3

Text describing packaging and labeling of Study Drug kits has been updated.

Section 5.4

Text describing storage and preparation of Study Drug has been clarified. A time window of 3 hours has been specified for completion of the 45-minute infusion after the IV bag containing Study Drug has been prepared.

Section 5.5

The following text has been deleted to conform to standard operating procedures which apply to disposal of biohazardous waste at some investigational sites. Used infusion bags and tubing will be disposed of following site standard procedures.

Deleted text: After the infusion has been completed, the used infusion bag should be returned to the pharmacist or individual designated as responsible for study drug and retained for drug accountability.

Section 8 Table 2 Schedule of Activities, Section 8.2.1 Screening and Randomization

Language has been modified to permit all screening assessments to be performed on the same day if practical. The Schedule of Activities Table has been adjusted to reflect this change. The new text in Section 8.2.1 reads:

> The following may be performed on the same day or on another day within the 21–day (+ 2 days) screening window:
>
> - Severe Impairment Battery (SIB)
> - Alzheimer Disease Cooperative Study Activities of Daily Living Inventory-Severe Impairment Version (ADCS-ADL-SIV)
> - Neuropsychiatric Inventory (NPI)
>
> The SIB, ADCS-ADL-SIV, and NPI may be administered at any time within the 21-day (+2) screening window, including the screening (first) day, allowing all procedures to be completed on the same day if practical.
>
> If the SIB and MMSE-2 are performed on the same day, the SIB should be performed first, followed by the MMSE-2.
>
> If the subject has had no intervening medical issues or changes in medications between the date the SIB is performed and Day 0, dosing may proceed as scheduled without repeating scale assessments.
>
> If changes in the subject's health or mental status occur during this period, including changes in medication affecting the mental status, the Medical Monitor should be notified. In this case, the subjects will need to have the SIB, MMSE-2, C-SSRS, ADCS-ADL-SIV, NPI repeated before they can be randomized. The site will need to continue to ensure eligibility requirements are met per the protocol regarding these scales.
>
> Sites will not be able to randomize subjects before confirmation that eligibility criteria have been met per the review of the Rosen-Modified Hachinski Scale, MMSE-2, and CSSRS by the Worldwide Clinical Trials' Clinical Analytics, Training, and Surveillance (CATS) group.

Section 17, Appendix 1, Restricted Concomitant Medications

> Multiple changes were made to the table of restricted medications, removing medications that have minimal effect on cognitive performance and testing. There are no known drug/drug interactions with bryostatin.

16.2 Summary of Changes, Version 3.0 15 July 2016

Except as noted, section numbers refer to the amended protocol section numbering.

Global changes:

The document version and date have been updated.

A number of grammatical corrections and clarifications were made to the text, which do not change the content. These have not been individually described in the summary of changes.

Title Page

Planned LPLV has been changed to February 2017.

Study Contact Information

Paula T. Trzepacz, MD has replaced Warren W. Wasiewski, MD as Chief Medical Officer.

Protocol Synopsis

Study duration has been changed to approximately 14 months.

Study Design

> Two doses of bryostatin, 20µg or 40µg, will be compared to placebo. Study subjects will receive a total of 7 doses of study drug over 12 weeks of treatment, followed by safety and efficacy assessments at Week 13 and a final study visit at Week 15. There will be no second randomization for additional treatment for those in the active drug arms nor will those in the placebo arm be randomized a second time to receive either 10 µg of bryostatin or continued treatment with placebo.
>
> Text describing the second randomization and second 12 weeks of treatment has been deleted.

Secondary Efficacy Endpoints (as described in Study Synopsis and Section 4.2.4)

> The timepoints at which secondary efficacy endpoints will be assessed has been specified as follows:
>
> - Change from baseline in the SIB at Weeks 5 and 9
> - Change from baseline in Alzheimer Disease Cooperative Study Activities of Daily Living Inventory-Severe Impairment Version (ADCS-ADL-SIV) score at Weeks 5, 9 and 13
> - Change from baseline in MMSE-2 score at Weeks 5, 9 and 13
> - Change from baseline in Neuropsychiatric Inventory (NPI) score at Weeks 5, 9 and 13
> - Clinical Global Impression of Improvement (CGI-I) at Weeks 5, 9 and 13

Exploratory Pharmacokinetic and Pharmacodynamic Endpoints

Peripheral blood mononuclear cells will be tested for PKCε concentration but not for activity as previously stated.

Statistical Considerations

(text in Protocol Synopsis and Section 11.0 have been revised as follows)

The primary contrasts will be the change from baseline in the SIB at Week 13 between each bryostatin group and the placebo group. These will be conducted sequentially, first for the bryostatin 40µg group, and if significant (at 1-sided level of 0.10), for the 20µg bryostatin group. The Bryostatin treatment groups will not be pooled.

To provide at least 80% power, it is assumed that a true mean difference of at least **6.5** (not 5.6) points in favor of the bryostatin groups (standardized effect size of **0.47**, not 0.40) will be observed, in a test between each bryostatin group and placebo in mean change from baseline in the SIB (one-sided at α=0.10). The least significant difference for the test between the observed means under these conditions is estimated to be **3.9** points (not 3.4), (effect size of 0.28 not 0.24).

Protocol Synopsis and Sections 4.3.1 and 4.3.2: Eligibility criteria

Inclusion #4 has been clarified to state that the MMSE-2 range of 4-15 is inclusive.

Inclusion #9 has been modified to allow a change in concomitant drugs for treatment of Alzheimer's disease under specific conditions. The text now reads: If taking drugs approved for treatment of Alzheimer's disease (e.g. cholinesterase inhibitors, memantine), must be on a stable dose for at least 3 months prior to entry into study and dose must not change during the study unless a change is required due to an adverse effect of the prescribed medication or a clinically significant change in the patient's status

Inclusion #11b has been rephrased as follows to specify use of contraceptives 30 days prior to dosing:

If not postmenopausal, agree to use a double method of contraception, one of which is a barrier method (e.g., intrauterine device plus condom, spermicidal gel plus condom) 30 days prior to dosing until 30 days after last dose and have negative human chorionic gonadotropin (β-hCG) test for pregnancy at screening.

Inclusion #12 has been modified to correspond with the change in Inclusion #11b: Males who have not had a vasectomy must use appropriate contraception methods (barrier or abstinence) from 30 days prior to dosing until 30 days after last dose

Exclusion criteria #5 and #6: The numbering of these criteria have been corrected in the Protocol Synopsis to match the order in which they occur in Section 4.3.2.

Exclusion #19 has been modified to address history of cancer. The text now reads: Acute or poorly controlled medical illness: blood pressure > 180 mmHg systolic or 100 mmHg diastolic; myocardial infarction within 6 months; uncompensated congestive heart failure [New York Heart Association (NYHA) Class III or IV]. History of cancer: the subject should be clear of cancer for at least 2 years prior to screening.

Section 3.0.3

References to the second 12 weeks of the study have been deleted.

Section 3.1 Hypothesis

The null hypothesis will be rejected at a significance level of α (1-sided) = 0.10. This analysis will be a test of superiority and only an improvement in the SIB score is of interest. The analysis will be conducted using a mixed model for repeated measures (MMRM). For this formal inferential testing, the bryostatin 40µg group will first be compared to the placebo group; if the null hypothesis is rejected, the bryostatin 20µg group will be then compared to the placebo group.

Section 4.0

Text describing the second randomization and details regarding dosing assignments in the second 12 weeks of the study have been deleted.

Figure 1: Study Design has been inserted.

Sections 4.2.5, 8.0.3 and 12.5 Pharmacokinetics

Peripheral blood mononuclear cells will be tested for PKCε concentration but not for activity as previously stated.

Section 5.5

Text referring to the second randomization has been deleted.

Section 5.7

Table 1: Dosing Schedule has been deleted. It has been replaced by Figure 1 in Section 4.0.

Remaining tables in the document have been renumbered accordingly.

Section 6

Reference to the second randomization has been deleted.

Section 6.2.1 Screening and Randomization

The screening period has been expanded to approximately 4 weeks to accommodate scheduling of imaging procedures. The SIB performed in screening must be done within 3 weeks before dosing, therefore if the screening period exceeds 3 weeks, the SIB should be repeated on the day of the first dose, prior to dose administration. The SIB score obtained closest to the day of dosing should be entered as the baseline value.

Section 6, Table 1 Schedule of Activities

The screening period has been expanded from 21 days to 28 days (4 weeks). This has been done to accommodate scheduling of imaging procedures, if needed. If the screening SIB is done more than 3 weeks before the date of the first dose, the SIB should be repeated on the day of dosing, prior to dose administration. The SIB value obtained on the day of dosing should be entered as the baseline SIB value.

Lab tests at screening will include TSH, and if indicated by abnormal TSH results, T-3 and T-4 will automatically be tested by the central lab.

Vital signs will be measured while the subject is in a supine position (previously unspecified).

A 5-minute time window has been added to Bryostatin level sample times, and vital sign measurements. This time window is also inserted in Sections 8.1.2 through 8.1.9, which describe study visit procedures.

Section 6.0.1.1 Laboratory

TSH has been added at screening, and T-3 and T-4 will be done if TSH result is abnormal.

Section 6.0.3 Pharmacokinetic and Pharmacodynamic Assessments

References to testing at doses 9, 12, and 14 have been deleted. A five-minute window has been provided for scheduled blood sampling.

Section 6.1.1 Screening and Randomization

Screening procedures and randomization will take place within an approximate 4-week period prior to first dose administration (Days -28 to -2). The previous screening period was 21 days.

TSH has been added to screening lab tests, with follow-up testing of T-3 and T-4 if TSH is abnormal.

Regarding changes in the subject's health or mental status that occur during the screening and randomization period, including changes in medication affecting the mental status, the following text has been revised to provide a case by case assessment of subject eligibility:

Old text: In this case, the subjects will need to have the SIB, MMSE-2, C-SSRS, ADCS-ADL-SIV, NPI repeated before they can be randomized.

New text: The Medical Monitor will advise the PI regarding any assessments that should be repeated to ensure eligibility requirements are met.

Section 6.1.10 Week 13 Day 91 (+/-2 days)

The following text has been deleted because no further dosing will occur:

Each subject will begin the newly assigned dose regimen at this visit, therefore, AEs will be collected prior to the infusion that reflect previous dosing and again after dosing.

Text describing the timing of vital sign measurements during infusion has also been deleted.

Sections 7.1.12 through 8.1.19 (section numbers from Version 2)

Sections referring to study visits in the second dosing period have been deleted.

Sections 8 and 9.5

References to efficacy data have been deleted. The DSMB will not review efficacy data.

Section 9 Data Analysis / Statistical Methods

The following text has been deleted, since it refers to the second dosing period:

The study will be conducted and analyzed in two parts. In the first part subjects will be randomized in equal numbers to one of two doses of bryostatin and Placebo and assessments will be scheduled during and after the first 12 weeks of treatment. After 12 weeks of treatment subjects in each dose group will be randomly assigned in equal numbers to either continue on their original treatment or begin a new dose of bryostatin.

Section 9.3 Efficacy Analysis

All statistical tests will be one-sided and use $\alpha = 0.10$ unless otherwise stated. LSM and two-sided 80% confidence intervals will be provided for treatment group differences and estimated endpoint values by visit. The following hierarchal procedure will be used for the formal inferential testing of the change from baseline in the SIB at Week 13: first the bryostatin 40µg group will be compared to the placebo group; if the null hypothesis is rejected, the bryostatin 20µg group will be then compared to the placebo group. Other than using the above stepwise inferential testing procedure for formal testing of the primary contrasts, no multiplicity adjustments will be made for analysis of the secondary endpoints. Whether or not the formal inferential testing yields rejection of the null hypothesis, estimates of effect with confidence intervals and p-values comparing each dose of bryostatin to placebo will be provided for all endpoints.

Section 9.3.1

The primary contrast will be the change from baseline in the SIB at Week 13 between each bryostatin group and the placebo group, using the hierarchical procedure described above for the formal hypothesis testing.

Section 9.3.2

The ADCS-ADL-SIV, MMSE-2, and NPI secondary endpoints will be analyzed using the statistical model used in the analysis of the SIB.

Section 12.7 Primary Efficacy Analysis (as numbered in version 2.0 of the protocol) has been deleted.

The following text has been deleted since the study will not be ongoing at the time of data analysis.

The primary analyses of the safety and efficacy endpoints is to be based on the data through the Week 13 visit. A complete unblinded analysis of the safety and efficacy data will be conducted on all data for the study. This will be conducted after all subjects have completed the Week 13 visit (or have discontinued), all data has been collected, checked and is final, and the database for the study has been locked.

Measures will be taken to prevent unblinding of individual study subjects and their data until completion of the study and final database lock.

The unblinded summaries from this analysis (data through Week 13) will be provided to the sponsor for the purpose of informing decisions about future development of bryostatin. The sponsor may also make public announcements regarding the study's meeting its primary safety and efficacy endpoints.

Since the study will be ongoing at the time of this analysis, the individual treatment assignment for all subjects should remain blinded.

16.3 Summary of Changes, Version 4.0 1 March 2017

Global changes:

The document version and date have been updated.

Study Contact Information

Kenneth Gorelick, MD has replaced Paula T. Trzepacz, MD as Acting Chief Medical Officer.

Protocol Synopsis

Exploratory Pharmacokinetic and Pharmacodynamic End Points: a population pharmacokinetic model will not be used to summarize bryostatin exposure in plasma.

Peripheral blood mononuclear cell (PBMC) Protein Kinase C epsilon (PKCε) amount at various time points may be determined.

Statistical Considerations: The primary contrasts will be the change from baseline in the SIB at Week 13 between each bryostatin group and the placebo group and between the combined (pooled) bryostatin dosing group and the placebo group.

Section 3.1 Hypothesis

Analyses will be performed on each bryostatin dosing group vs. placebo and on the "pooled" bryostatin dosing group vs. placebo. A stepwise hierarchal testing procedure will not be followed.

Section 4.3.5 Exploratory Pharmacokinetic and Pharmacodynamic End Points

Peripheral blood mononuclear cell PKCε amount may be assessed at various time points.

Section 9.3 Efficacy Analysis

Analyses will be performed on each dosing group vs. placebo and on the "pooled" dosing group vs. placebo.

The primary analysis will test the hypothesis that there is a beneficial effect of each bryostatin dose group or the combined (pooled) bryostatin group compared to placebo on the change from baseline in the SIB at the Week 13 visit.

The secondary efficacy endpoints will be analyzed in all 3 groups (i.e., each dose separately and pooled vs. placebo); p-values and confidence intervals will be reported and used to assist in interpretations of the results.

A hierarchal procedure will not be used for the formal inferential testing of the change from baseline in the SIB at Week 13.

Estimates of effect with confidence intervals and p-values comparing each dose of bryostatin to placebo and the pooled bryostatin group to placebo will be provided for all endpoints.

Section 9.4 Pharmacokinetics

A population pharmacokinetic model will not be used.

Plasma samples may be analyzed to determine PKCε concentrations in PBMCs. Dose-response relationships may be examined, as well as possible exposure-response relationships.
