# Supplementary material for: A Randomized, Double-Blind, Placebo-Controlled, Phase II Study Assessing Safety, Tolerability, and Efficacy of Bryostatin in the Treatment of Moderately Severe to Severe Alzheimer’s Disease
Source: J Alzheimers Dis. 2019 Jan 22;67(2):555–70. doi: 10.3233/JAD-180759 (PMC6398557; doi:10.3233/JAD-180759)
Supplement: Supplementary Material 2 [file jad-67-jad180759-s002.pdf]

Statistical Analysis Plan

Title: A Randomized, Double-Blind, Placebo-Controlled, Phase 2 Study Assessing the Safety, Tolerability and Efficacy of Bryostatin in the Treatment of Moderately Severe to Severe Alzheimer's Disease

Protocol Number: NTRP101-202

Version Final

Issue Date: 22-Mar-2017

Author: Shuhua Qi, MSc

Previous Versions

SAP Amendments before database lock

Version	Issue Date	Section	Revision/Addition	Rationale
Final	22Mar2017	6.1.6	Added Complete analysis set	Sponsor request
Final	22Mar2017	11	Added 4 Tables for Complete set and modified few table number accordingly	Sponsor request/agreement
Final	17Mar2017	6.10.2	Updated SAS code	Sponsor agreement
Final	14Mar2017	Page 1	Updated protocol version	Protocol amendment 4
Final	14Mar2017	6.10.2, 6.10.4, 6.10.6	1) Re-organizes.<br>2) Added following sentence in section 6.10.6:<br>The effects of center and treatment by center interaction will be explored using an analysis of covariance (ANCOVA) model as describe in section 6.10.4 if data permit. Low enrolling centers may be pooled if appropriate. Stepwise covariates selection approach may be applied. These will explored as post hoc analyses.<br>3) the followings were removed<br>- Correlation of change in (SIB, ACDC-ADL, NPI, etc) with bryostatin Cmax and AUC	Sponsor request/agreement

			- Correlation of change in (SIB ACDC-ADL, NPI, etc) with mean change in serum PKCepsilon levels	
Draft v4	14Feb2017	3.3	Removed percent from baseline in SIB score	Not in protocol
		6.1.4	Removed PPS	Sponsor request
		6.2.8.1	Replaced by original/draft1 SAP wording for calculation of dose taken and percentage of planned dose taken	The analysis can/will take place after unblinding per sponsor request
		6.10.1, 6.10.2	Revised primary analysis method	Protocol amendment XXX
		6.10.3	Sensitivity analysis was not planned	Sponsor request
		6.10.4, 6.10.5 and 6.10.6	Revised/added exploratory analysis methods	Sponsor request
		6.12.3	Added vital BP based on CTCAE grade	Sponsor request
Draft v3	02Feb2017	6.11	WCT PK group will proved summary table/listings for PKCε concentration	WCT PK group agreement
		Last page	Approval for implementation of Statistical Analysis Plan	
Draft v2	27Dec2016	Cover page	Changed author	Transition,12Dec2016
		Original 6.2.20, 6.11	Removed section 6.2.20 Calculation of Area under the Curve and section 6.11 Pharmacokinetic Analyses	A separate PK data analysis plan (PAP).

		2.8.1	Revised Calculation of dose taken and percentage of planned dose taken	Sponsor agreement
		6.12.2, 6.12.3 & 6.12.3	Added figures for Lab, vital signs and ECG	Sponsor request

Table of Contents

1 INTRODUCTION 8
2 STUDY OBJECTIVES 8
3 ENDPOINTS 9
3.1 Primary Endpoint 9
3.1.1 Safety 9
3.1.2 Efficacy 9
3.2 Secondary Endpoints 9
3.2.1 Safety 9
3.2.2 Efficacy 9
4 SAMPLE SIZE 10
5 RANDOMIZATION 10
6 PLANNED ANALYSES 10
6.1 Analysis Sets 11
6.1.1 Enrolled Set 11
6.1.2 Randomized Set 11
6.1.3 Full Analysis Set 11
6.1.4 Per-Protocol Set 11
6.1.5 Safety Analysis Set 11
6.1.6 Complete Analysis Set 11
6.2 Derived Data 11
6.2.1 Age 12
6.2.2 Screening 12
6.2.3 Baseline and Change from Baseline 12
6.2.4 Duration/Study Day/Time 12
6.2.5 Conventions for Missing and Partial Dates 12
6.2.6 Missing/Partial Start/Stop Date of Adverse Events and Concomitant Medications 12
6.2.7 Missing Diagnosis Dates 14
6.2.8 Exposure to Study Drug 14
6.2.9 Inexact Values 15
6.2.10 Study Period/Analysis Windows 15

6.2.11 Diagnosis of AD 16
6.2.12 Severe Impairment Battery (SIB) 16
6.2.13 Alzheimer Disease Cooperative Study Activities of Daily Living Inventory-Severe Impairment Version (ADCS-ADL-SIV) 17
6.2.14 Mini Mental State Examination 2 (MMSE-2) 17
6.2.15 Neuropsychiatric Inventory (NPI) 18
6.2.16 Clinical Global Impression of Improvement (CGI-I) 19
6.2.17 Rosen-Modified Hachinski Scale 19
6.2.18 Electrocardiogram Data 19
6.2.19 Columbia-Suicide Severity Rating Scale 19
6.2.20 Unscheduled Visits 20
6.3 Conventions 20
6.3.1 Decimal Places 21
6.4 Subject Disposition 22
6.5 Protocol Deviations 22
6.6 Baseline Comparability 22
6.7 Medical History 23
6.8 Prior and Concomitant Medications 23
6.9 Exposure to Study Drug/Treatment compliance 24
6.10 Efficacy Analyses 24
6.10.1 Primary Endpoint 24
6.10.2 Primary Efficacy Analysis 25
6.10.3 Sensitivity Analysis 25
6.10.4 Exploratory Analysis 26
6.10.5 Secondary Endpoints 26
6.10.6 Exploratory Endpoints 27
6.10.7 Multiplicity 27
6.11 Pharmacodynamic Analyses 27
6.12 Safety Analyses 28
6.12.1 Adverse Events 28
6.12.2 Laboratory Data 29
6.12.3 Vital Signs 30
6.12.4 Electrocardiogram Data 32
6.12.5 Physical Examination 32

6.12.6 Second Treatment Period Analyses 32
7 INTERIM ANALYSIS 33
8 DATA SAFETY MONITORING BOARD ANALYSIS 33
9 CHANGES TO PLANNED PROTOCOL ANALYSIS 33
10 REFERENCES 34
11 LIST OF TABLES, FIGURES AND LISTINGS 35

1 INTRODUCTION

This document details the planned statistical analyses of non-pharmacokinetic (PK) data for Neurotrope BioScience , protocol "NTRP101-202" study titled "A Randomized, Double-Blind, Placebo-Controlled, Phase 2 Study Assessing the Safety, Tolerability and Efficacy of Bryostatin in the Treatment of Moderately Severe to Severe Alzheimer's Disease".

The proposed analyses are based on the contents of the final version of the protocol (dated 17-Apr-2015) and amendments 2.0, 3.0 and 4.0 (dated 20-Jan-2016, 15-Jul-2016 and 03-Mar-2017, respectively).

This is a randomized double-blind placebo-controlled Phase 2 study comparing different doses of bryostatin for the treatment of moderately severe to severe Alzheimer's disease (AD). The study was originally 28 weeks in duration, including two 12-week treatment phases where eligible subjects were randomized to 1 of 3 treatments (bryostatin 20µg, bryostatin 40µg or placebo) for the first 12 weeks of the study. At the end of the 12 weeks, subjects in the 2 active treatment arms were to be randomized to continue on the initial dose or to receive an alternate dose (bryostatin 20µg or 40µg, depending on the initial bryostatin dose received) for an additional 12 weeks and subjects in the placebo arm were to be randomized either to continue on placebo or to receive bryostatin 10µg for an additional 12 weeks. Amended protocol version 3.0 (dated 15-Jul-2016), dropped the second 12-week treatment phase from the study design. This study is now 15 weeks in duration, including a safety and efficacy evaluation 30 days after the last dose of study drug.

Details of the proposed PK analysis will be covered by WCT Bioanalytical Sciences Group prior to the pharmacokinetic analysis in a separate PK data analysis plan (PAP). The PAP will describe the analysis population the modeling methods and possible covariates that will be examined, as well as the tables, figures, and listings.

2 STUDY OBJECTIVES

Primary objective:

- To evaluate the safety and tolerability of bryostatin 1 (hereafter referred to as bryostatin) for the treatment of moderately severe to severe AD.

Secondary objective:

- To evaluate the efficacy of bryostatin in the treatment of moderately severe to severe AD.

Exploratory objectives:

- To characterize the pharmacokinetics (PK) of bryostatin in subjects with AD following administration of 20 or 40µg of bryostatin administered as a 45 minute intravenous (IV) infusion over 12 weeks of treatment.

- To characterize the pharmacodynamics of bryostatin in subjects with AD following administration of 20 or 40µg of bryostatin administered as a 45 minute IV infusion over 12 weeks of treatment.

3 ENDPOINTS

3.1 Primary Endpoint

3.1.1 Safety

- Treatment emergent AEs (TEAEs)
- Treatment emergent SAEs

3.1.2 Efficacy

- Change from baseline in the Severe Impairment Battery (SIB) score at Week 13

3.2 Secondary Endpoints

3.2.1 Safety

- Vital signs
- Hematology
- Blood chemistry
- Physical examination (PE) including body weight
- Electrocardiogram (ECG) parameters
- Columbia-Suicide Severity Rating Scale C-SSRS

3.2.2 Efficacy

- Change from baseline in the SIB at Weeks 5 and 9
- Change from baseline in Alzheimer Disease Cooperative Study Activities of Daily Living Inventory-Severe Impairment Version (ADCS-ADL-SIV) score at Weeks 5, 9, and 13
- Change from baseline in Mini-Mental State Examination (MMSE)-2 score at Weeks 5, 9, and 13
- Change from baseline in Neuropsychiatric Inventory (NPI) score at Weeks 5, 9, and 13

- Clinical Global Impression of Improvement (CGI-I) at Weeks 5, 9, and 13

4 SAMPLE SIZE

Since the primary objective of the study is to obtain safety and tolerability data for the administration of multiple doses of bryostatin, the planned sample size is based largely on feasibility. However, the following is an assessment of power for the primary analysis of primary efficacy endpoint, estimation of the effect of bryostatin on the mean change from baseline in the SIB at Week 13.

It is estimated that 150 subjects equally randomized among the 3 dosing groups (2 doses of bryostatin and placebo) will provide at least 80% power, assuming a true mean difference of at least 6.5 points in favor of the bryostatin groups (standardized effect size of 0.47), in a test between each bryostatin group, and the polled bryostatin groups, and placebo in mean change from baseline in the SIB, (one-sided at α=0.10). This assumed the standard deviation of change from baseline at Week 13 is 14 points. This estimate allowed that up to 15% of randomized subjects would not provide information due to discontinuation. The least significant difference for the test between the observed means under these conditions was estimated to be 3.9 points (standardized effect size of 0.28).

5 RANDOMIZATION

Using a stratified randomization based on MMSE-2 score (4-9 vs. 10-15), eligible subjects were randomized 1:1:1 to 1 of 3 treatment arms: bryostatin 20µg, bryostatin 40µg or placebo.

Prior to amendment 3, after 12 weeks of treatment, eligible subjects were randomized for an additional 12 treatment period. Initial MMSE-2 scores were used for stratification. Subjects in the 20µg group were randomized 1:1 either to the same dose or to the 40µg dose. Subjects in the 40µg group were randomized 1:1 either to the same dose or to the 20µg dose. Subjects in the placebo group were randomized 1:1 either to continue on placebo or to the bryostatin 10µg dose.

6 PLANNED ANALYSES

This study contained 2 double-blind treatment phases/periods until protocol amendment 3.0. Subjects that were in the second treatment period discontinued the study once protocol amendment 3.0 was approved. The primary treatment period of interest is the initial treatment period and hereafter treatment period references this initial treatment period. The second treatment period will be referred to as treatment period 2 and unless otherwise stated, these data will only be listed. For full definition, see Section 6.2.10.2.

No statistical analysis plan (SAP) prepared in advance of the data can be absolutely definitive and the final clinical study report (CSR) may contain additional tables or statistical tests if

warranted by the data obtained. The justification for any such additional analyses will be fully documented in the final CSR.

6.1 Analysis Sets

Subjects excluded from the analysis sets and the reason for their exclusion will be listed in Appendix 16.2.

6.1.1 Enrolled Set

The Enrolled Set (ES) includes all subjects screened or those who give informed consent and are allocated a subject number. Subjects who are rescreened and assigned a new subject number will be counted only once.

6.1.2 Randomized Set

The Randomized Set (RS) consists of all subjects that are randomized.

6.1.3 Full Analysis Set

The Full Analysis Set (FAS) used for efficacy analyses, consistent with the intent-to-treat (ITT) principles, is defined as all randomized subjects who receive at least 1 dose of study medication and who have at least 1 post-baseline efficacy assessment.

6.1.4 Per-Protocol Set

A Per Protocol Analysis Set (PP) is not planned. Major protocol deviations will be summarized. A supportive data listing of protocol deviations (major/minor) will be provided.

6.1.5 Safety Analysis Set

The Safety Analysis Set (SA) is defined as all randomized subjects who receive any study medication (either partial or completed infusions of Bryostatin or Placebo). For safety analysis subjects will be grouped on an as-treated basis. If subjects received different doses over the course of the study, they will be grouped according to the dose most often administered. In addition, all treated subjects will be grouped and compared to placebo subjects.

6.1.6 Complete Analysis Set

The completer set includes all subjects in the FAS who have completed the week 13 evaluation of SIB.

6.2 Derived Data

This section describes the derivations required for statistical analysis. Unless otherwise stated, variables derived in the source data will not be re-calculated.

6.2.1 Age

Age at screening will be calculated in SAS as

age = floor ((intck('month',*birthdate*,*date*) - (day(*date*) < day(*birthdate*))) / 12);

6.2.2 Screening

For each variable where a subject is re-screened, his/her latest non-missing value from the original or re-screening assessment will be taken for tabulation purposes.

6.2.3 Baseline and Change from Baseline

Baseline is defined as the last non-missing value (either scheduled, unscheduled or repeat) before the subject receives first dose of study medication (Week 0).

Change from baseline CHG=AVAL-BASE where AVAL is the post-baseline value, BASE is the baseline value.

6.2.4 Duration/Study Day/Time

Study day will be calculated as the number of days from first dose of study medication (ie, for the analysis, the study day is relative to the first dose of study medication which will be deemed "Day 1"; note that this is a different definition than the one in Protocol NTRP-1010-202 V4, where the first dose of the study medication is given on Day 0).

- date of event – date of first dose of study drug + 1, for events on or after first dose
- date of event – date of first dose of study drug, for events before first dose.

Duration (e.g., event duration/medication usage) = stop date-start date +1

6.2.5 Conventions for Missing and Partial Dates

All rules explained below for partial/missing dates will be followed unless contradicted by any other data recorded on the electronic Case Report Form (eCRF).

All dates presented in the individual subject listings will be as recorded on the eCRF (i.e., not completed as per the rules below).

6.2.6 Missing/Partial Start/Stop Date of Adverse Events and Concomitant Medications

<u>Partial or missing stop date will be imputed as follows:</u>

If the stop date is completely missing and the event has resolved or the subject has stopped taking the concomitant medication, the stop date will be imputed as the date of the subject's last clinic visit in the study.

- If only the year is known, the stop date will be imputed as "31-Dec" of that year or as the date of the subject's last clinic visit in the study if in the same year.
- If the month and year are known, the stop date will be imputed as the last day of that month unless the stop date corresponds to the same month as the subject's last clinic visit in which case the date of the subject's last clinic visit in the study will be used instead.

Missing start date will be imputed as follows:

- If the stop date occurs on or after the start of study drug or the event/concomitant medication is ongoing, the start date will be imputed as the date of the first dose of study drug.
- If the stop date occurs before the start of study drug, the start date of the event/concomitant medication will be imputed as the subject's screening date or the stop date of the event/concomitant medication whichever the earlier.

Partial start date (year present, but month and day missing):

- If the stop date occurs on or after the start of study drug or the event/concomitant medication is ongoing, and the year is the same as the year of first dosing the start date will be imputed as "01-Jan" of the same year or the date of the first dose of study drug whichever is later. If the year is different from the year of first dosing "01-Jan" will be used.
- If the stop date occurs before the start of study drug, the start date of the event/concomitant medication will be imputed as the "01-Jan" of the same year.

Partial start date (month and year present, but day missing):

- If the stop date occurs on or after the start of study drug or the event/concomitant medication is ongoing, the start date will be imputed as the first day of the same month and year unless this partial start date is in same month as the first dose of study drug in which case the date of first dose of study drug will be used.
- If the stop date occurs before the start of study drug, the start date will be imputed as the first day of the month and year of the partial stop date.

Missing start/stop time of adverse events:

- If the start time of the adverse event (AE) is missing, it will imputed only in the case where the start date of the AE corresponds to the date of the first dose of study drug. The

time will be imputed as the same time as the first dose of study drug. In all other cases the time will not be imputed.
- If the stop time of the adverse event is missing, it will be imputed as 23:59 for stop time.

6.2.7 Missing Diagnosis Dates

If the month and year are present but the day is missing, the diagnosis date will be set to the first day of the relevant month. If only the year is recorded the diagnosis date will be set as “01-Jan” for that year.

6.2.8 Exposure to Study Drug

6.2.8.1 Calculation of dose taken and percentage of planned dose taken

Per protocol, study drug (bryostatin or placebo sterile lyophilized powder in 10 ml vials) should be reconstituted with 1 mL of PET diluent (resulting in 50 µg bryostatin per mL or 0.0 µg bryostatin per mL). After swirling the vial to completely dissolve the contents, the resulting solution must be diluted immediately with 9 mL of 0.9% sodium chloride (NS) injection, United States Pharmacopeia (USP). The necessary volume of this solution to achieve the assigned dose for the subject should then be added to an IV infusion bag containing 50 mL of normal saline.

Thus, the total volume of the infusion varies depending on the randomized treatment.

After diluting the 1 mL PET solution with 9 mL of 0.9% sodium chloride (NS), the resulting solution will result in 5 µg bryostatin per mL or 0.0 µg bryostatin per mL.

Subjects are supposed to receive a total of 7 doses with the first 2 doses being loading doses. The following table lists the total volume of 1 infusion for each treatment group.

Dose	1 infusion (total volume in mL)
Placebo	50 mL
20 µg	54 mL
24 µg (loading dose)	54.8 mL
40 µg	58 mL
48 µg (loading dose)	59.6 mL

The following table details total planned doses in terms of planned volume for 7 doses for each randomized treatment group. It also states the criteria to determine if a subject received at least 80% of his/her planned dose.

Dose	Planned dose in terms of planned volume	80% planned dose
Placebo	350 mL	280 mL
20 µg	379.6 mL	303.7 mL
40 µg	409.2 mL	327.4 mL

6.2.9 Inexact Values

In the case where a variable is recorded as “>x”, “≥x”, “<x” or “≤x”, then for analysis purposes a value of x will be taken, with x fulfilling the record of the variable. Where a range of values is quoted the midpoint of the range will be taken.

6.2.10 Study Period/Analysis Windows

6.2.10.1 Pre-Treatment Period

The Pre-Treatment Period is defined as the period of time between the subject signing the informed consent through immediately prior to the subject receiving the first dose of study medication.

6.2.10.2 Treatment Period

The primary treatment period of interest is the initial treatment period and will be referred to as treatment period. The second treatment period will be referred to as treatment period 2.
The treatment period starts on the day and time of first dose of study medication and ends on Week 13 Visit. This is the primary treatment period of interest.

For those subjects that were randomized for the second treatment period and had the first dose of second treatment period study medication on Week 13 visit, the treatment period ends prior to the infusion date and time for first dose of the second treatment period on Week 13 Visit. Hence any data collected prior to the infusion date and time on Week 13 Visit would be assigned to the initial treatment period. The data collected after that date and time will be assigned to treatment period 2 unless otherwise stated. For assessments at Week 13 where only date was collected and per protocol the assessments were pre-dose, these would be assigned to the initial treatment period.

6.2.10.2.1 Early Termination efficacy assessments

For analysis purposes, efficacy data collected from early termination (ET) visit will be assigned to the next scheduled visit. This only pertains to ET visits that occur during the treatment period.

Subjects who complete the treatment period but discontinue during the second treatment period are considered completers in the main treatment period analyses.

6.2.10.2.2 30-Day Follow-Up Visit

A follow-up visit should be scheduled 30 days after the last dose of study drug for all subjects that complete or are withdrawn from the study.

Follow-up visits will be assigned to the treatment period of the last dose of study drug taken by subject (and hence summarized under the treatment assigned for that treatment period.).

For details regarding concomitant medications and AEs please see corresponding Sections 6.8 and 6.12.1.

6.2.11 Diagnosis of AD

Diagnosis of AD is collected in medical history. Medical history is coded using MedDRA version 18.1. Years from Diagnosis of AD to Screening will be calculated based on preferred term for AD. Terms to be pulled will be finalized prior to unblinding.

6.2.12 Severe Impairment Battery (SIB)

The SIB is used to assess cognition in subjects with moderate and severe AD. There are forty questions that are divided into 9 subscales that include attention, language, orientation, memory, praxis, visuospatial ability, construction, social skills, and orienting head to name. Non-verbal responses are allowed, thus decreasing the need for language output. Most items have a point range of 0-2 except for item 19 and 24. The point score range for SIB total score (sum of the 40 items) is 0-100. Lower scores indicate greater cognitive impairment.

	Items	Point range
SIB Total Score	All	0-100
Social interaction	1 (a,b,c)	0-6
Orientation	3,5, 7	0-6
Visuospatial ability	27,29,31,33	0-8
Construction	35(a,b)	0-4
Language	4 (a,b),6,8 (a,b), 9(a,b,c),11 (a,b),13,15,17, 19(0-1 value),20,22, 24 (0-1 value),26,30 (a,b,c), 34(a,b),40	0-46
Memory	2, 10,14,25,28,32, 38	0-14
Praxis	16,18,21,23	0-8
Attention	12,36,37	0-6
Orienting to name	39	0-2

6.2.13 Alzheimer Disease Cooperative Study Activities of Daily Living Inventory-Severe Impairment Version (ADCS-ADL-SIV)

The ADCS-ADL-SIV is a 19-item functional assessment of the performance of activities of daily living for subjects with moderate to severe AD. Informants (Caregiver) are queried via a structured interview format as to whether subjects attempted each item in the inventory during the previous 4 weeks, as well as their level of performance. Each item is rated from the highest level of independent performance to complete loss. The questionnaire is split into two types of questions, an initial question relating to whether a subject has completed a particular activity and then a follow on question which scores how much assistance the subject has required if they have performed that particular activity. Note if a caregiver responds to the question relating to whether the subject performed a particular activity as no or don’t know this will contribute a zero to the total score. Total score (sum of the 19 items) range from 0-54 with lower scores indicating greater functional impairment.

6.2.14 Mini Mental State Examination 2 (MMSE-2)

The standard version of the MMSE-2, with 2 alternate forms, is used in this study. The MMSE-2 is a brief, widely used test for assessing overall cognitive state. The MMSE-2 (sum of 11 items) measures selected aspects of cognition such as memory, orientation, attention, language, and praxis on a scale of 0-30. Lower scores indicate greater cognitive impairment.

6.2.15 Neuropsychiatric Inventory (NPI)

The NPI is a behavior rating scale composed of a 12-item structured interview of the caregiver that is scored from 0 to 144 (the higher the score, the greater the psychiatric disturbance). The NPI assesses 12 behavioral and psychological disturbances occurring in dementia patients: delusions, hallucinations, agitation/aggression, depression/dysphoria, anxiety, elation/euphoria, apathy/indifference, disinhibition, irritability, aberrant motor behavior, sleep and nighttime behavior disorders, and appetite and eating disorders. Both the frequency and the severity of each behavior are determined.

Scoring the NPI:

Category	Numeric values assigned
Frequency is rated as:	1 = Occasionally - less than once per week<br>2 = Often - about once per week<br>3 = Frequently - several times per week but less than every day<br>4 = Very frequently - daily or essentially continuously present
Severity is rated as:	1 = Mild - produces little distress in the patient<br>2 = Moderate - more disturbing to the patient but can be redirected by the caregiver<br>3 = Severe - very disturbing to the patient and difficult to redirect
Caregiver Distress is rated as:	0 = no distress<br>1 = minimal<br>2 = mild<br>3 = moderate<br>4 = moderately severe<br>5 = very severe or extreme.
Category	Numeric values assigned

The total score for each of the 12 domains is domain score = frequency x severity

Thus, for each behavioral domain there are 4 scores:

- Frequency
- Severity

- Total (frequency x severity)
- Caregiver distress

A total NPI score will be calculated by adding the total scores of the 12 domain scores together. The caregiver distress score is not included in the total NPI score. The total distress score is generated by adding together the scores of the 12 items of the NPI distress questions.

6.2.16 Clinical Global Impression of Improvement (CGI-I)

The CGI-I is used to assess global change in the subject's condition compared to baseline before treatment. This is a 7-point scale ranging from (1) very much improved to (7) very much worse. A score of 0 means that the scale was not assessed and hence this value is not included in any summary/analysis unless stated otherwise.

6.2.17 Rosen-Modified Hachinski Scale

The Rosen-Modified Hachinski Scale will be evaluated at screening to differentiate Alzheimer's type dementia from multi-infarct dementia. The 8-item scale results in a score of 0-12.

6.2.18 Electrocardiogram Data

For ECG data recorded on continuous scales, if more than 1 value is recorded at a time point, the mean value rounded to the integer will be presented. For overall interpretation if more than 1 value is recorded, the most severe (worst case) of the respective readings will be taken. -

6.2.19 Columbia-Suicide Severity Rating Scale

The following outcomes are C-SSRS categories and have binary responses (yes/no). The categories have been re-ordered from the actual scale to facilitate the definition of composite endpoints:

Category 1	Wish to be Dead
Category 2	Non-specific Active Suicidal Thoughts
Category 3	Active Suicidal Ideation with Any Methods (Not Plan) without Intent to Act
Category 4	Active Suicidal Ideation with Some Intent to Act, without Specific Plan
Category 5	Active Suicidal Ideation with Specific Plan and Intent
Category 6	Preparatory Acts or Behavior
Category 7	Aborted Attempt
Category 8	Interrupted Attempt
Category 9	Actual Attempt (non-fatal)
Category 10	Completed Suicide

Suicidal Ideation since baseline – A "yes" answer at any time during double-blind treatment to any one of the 5 suicidal ideation questions (categories 1-5) on the C-SSRS.

Suicidal Behavior since baseline – A "yes" answer at any time during double-blind treatment to any one of the 5 suicidal behavior questions (categories 6-10) on the C-SSRS.

There will be no imputation of missing data for C-SSRS.

6.2.20 Unscheduled Visits

In general, only scheduled post-baseline laboratory and vital signs values will be tabulated. However, post-baseline repeat/unscheduled assessments could be included if an "any time, post-baseline" summary is specified. At minimum, these post-baseline assessments will be listed in the relevant appendices to the CSR.

6.3 Conventions

All data listings, summaries, figures and statistical analyses will be generated using SAS version 9.3 or higher[1].

Summaries will be presented by treatment group or overall. Treatment group labels will be displayed as follow:

Placebo	Bryostatin (20 µg)	Bryostatin (40 µg)	Bryostatin (20+40 µg)

Overall columns are to be included within the table shells as follows:

Demography	*Treatment and overall*
Baseline	*Treatment and overall*
Disposition	*Treatment and overall*
Efficacy	*Treatment*
PD	*Treatment*
AEs	*Treatment & overall*
Other safety	*Treatment*

Listings will be sorted in the following order: treatment group, subject, parameter, and visit unless otherwise stated.

Continuous variables will be summarized by the number of non-missing observations, mean, median, standard deviation, and minimum and maximum.

Categorical variables will be summarized by presenting the frequency and percent. Percentages will be based on the number of non-missing observations or the subject population unless otherwise specified. For each variable, all categories will be shown. Zero frequencies (but not the percent) within a category will be presented.

6.3.1 Decimal Places

Decimal places for derived data will be determined by the scale of measurement unless otherwise stated. No decimal places will be displayed if the smallest calculated value is ≥ 100; 1 decimal place will be displayed when the smallest value is within the interval (10, 100), with 10 being inclusive; 2 decimal places will be displayed when the smallest value is within (1, 10), with 1 being inclusive; and so on for even smaller scales of measurement.

For derived data where known in advance that the result will be an integer (e.g., day, month, year, number of days and total scores [for rating scales]) will be presented with zero decimal places unless otherwise stated.

Means, medians, and percentiles will be displayed to one more decimal place than the data**,** dispersion statistics (e.g., standard deviation) will have 2 more decimal places, and the minimum and maximum will be displayed to the same number of decimal places as reported in the raw data. Percentages will be displayed with 1 decimal place.

P-values will be quoted to 3 decimal places. P-values < 0.001 will be presented as p<0.001. Where p < 0.05, p < 0.01, or p < 0.001, attention will be drawn to this fact using the conventional "*", "**" or "***" annotation, respectively.

6.4 Subject Disposition

Subject disposition will be summarized as follows:

- The number of subjects, who were enrolled in the study, were randomized, and are in each analysis set will be summarized by treatment group and overall for the ES.
- The number of subjects from the main treatment period that were randomized into treatment period 2 and received study medication for the SA set.
- The number of subjects who were rescreened, failed screening and the reasons for screen failure will be tabulated for ES
- The number of treatment completers, early withdrawals and the reasons for withdrawals will be tabulated by treatment group and overall for SA and FAS set.
- The number of subjects present at each scheduled visit and number of subjects whose SIB were not done within 21 days before the first study drug dose will be summarized by treatment group for the SA set.
- Number of SA set subject by site will be summarized.

6.5 Protocol Deviations

A summary of major protocol deviations will be summarized.

Furthermore a listing of protocol deviations will be provided in Appendix 16.2.

6.6 Baseline Comparability

The comparability of treatment group with respect to subject demographics and baseline characteristics will be assessed in a descriptive manner, but no formal statistical testing will be performed.

Standard continuous or categorical variable summaries will be presented by treatment group for the SA and FAS set.

Demographic and Baseline Characteristics

The following variables will be summarized:

- Age at screening visit (years)
- Gender (Male, Female)
- Ethnicity (Hispanic or Latino, Not Hispanic or Latino)
- Race (American Indian or Alaska Native, Asian, Black or African American, Native Hawaiian or Other Pacific Islander, White)
- MMSE-2 (continuous, stratum [4-9 vs 10-15])
- Height, weight and BMI at screening
- Rosen-Modified Hachinski Score
- Years from diagnosis of AD to Screening (derived based on medical history)
- Concurrent use of AD drugs by type (cholinesterase inhibitor, memantine, both)

6.7 Medical History

Separate tabulations of previous and ongoing conditions at screening will be presented by treatment group and overall for the SA Set. Conditions will be presented *by Medical Dictionary of Regulatory Activities* (MedDRA version 18.1) primary system organ class and preferred term.

6.8 Prior and Concomitant Medications

Separate tabulations will be produced for prior and concomitant medications presented by treatment group and overall for the SA and FAS Set. Medications will be summarized using Anatomical Therapeutic Chemical (ATC) level 2 and drug reference name using the World Health Organization (WHO) dictionary (Version WHODDE MAR2015).

Medications will be categorized by start and stop dates of medication in relation to start date of study drug and could be categorized in more than one category (e.g., a medication taken prior to first dose of study drug but on-going through the treatment period (s), will be considered both a prior and concomitant medication). Prior medications are defined as all medications starting before the date of first dose of study medication. Concomitant medications are defined as

medications taken on or after the date of first dose of study medication up to the date of last study treatment in the main treatment period. For subjects who received study medication during treatment period 2, medications started on the same day as first dose of the second treatment period, will be assigned to that second treatment period only.

Frequency distribution of drugs used to treat AD by treatment arm will be presented.

6.9 Exposure to Study Drug/Treatment compliance

Number and percentage of subjects who did or did not have an infusion, had a full infusion, had partial infusion by treatment group and visit will be presented. Descriptive statistics of the number of infusions will be presented by treatment group.

Number and percentage of subjects who had 80% of the (cumulative) planned dose will be summarized by treatment group (see Section 6.2.8.1).

6.10 Efficacy Analyses

Efficacy analyses will be based on FAS by randomized treatment regardless of the treatment actually received. Descriptive statistics by treatment group and visit will be reported for all efficacy endpoints. Analyses will be performed on each dosing group vs. placebo and on the "pooled" dosing group vs. placebo.

6.10.1 Primary Endpoint

The primary efficacy objective is to determine if treatment with bryostatin (20 or 40 µg) improves cognitive function as measured by improvement on SIB after 12 weeks of treatment compared to placebo.

The primary efficacy endpoint is change from baseline in the Severe Impairment Battery (SIB) score at Week 13. Each bryostatin group will be compared to the placebo group and the pooled bryostatin group to placebo group.

The hypothesis test is as follows:

H_o: $\mu_{bryostatin} \leq \mu_{placebo}$

H_1: $\mu_{bryostatin} > \mu_{placebo}$

The null hypothesis will be rejected at a significance level of α (1-sided) = 0.10. There will be no p-value adjustment for multiplicity.

6.10.2 Primary Efficacy Analysis

The primary endpoint will be analyzed using Mixed Model for Repeated Measures (MMRM) with fixed effects for treatment, baseline MMSE-2 stratum, scheduled visit (nominal) and scheduled visit by treatment interaction, random effect for subject and baseline SIB as a covariate. The model will include two treatment groups (i.e. placebo and one bryostatin dose group). All statistical tests will be 1-sided with $\alpha = 0.10$. Least-square means (LSM) and 2-sided 80% confidence intervals (CI) will be provided for treatment group differences and estimated endpoint values by visit.

Sample SAS code for two-sided 80% confidence interval.
Treat: 1=Placebo, 2=study drug
Visit 13: Week 13

```
PROC MIXED DATA = <DATASET>;
  CLASS TREAT VISIT BASE_MMSE_STRATUM SUBJID;
  MODEL CHANGE = TREAT VISIT TREAT*VISIT BASE_MMSE_STRATUM
BASELINE_SIB/
                        DFM=KENWARDROGER ;
  REPEATED VISIT / TYPE=UN  SUB=SUBJID  GROUP=TREAT;
  LSMEANS TREAT*VISIT/PDIFF=CONTROL('1'  '13') ALPHA=0.2;
RUN;
```

Sample SAS code for one-sided p-value and 80% confidence interval.

```
PROC MIXED DATA = <DATASET>;
  CLASS TREAT VISIT BASE_MMSE_STRATUM SUBJID;
  MODEL CHANGE = TREAT VISIT TREAT*VISIT BASE_MMSE_STRATUM
BASELINE_SIB/
                          DDFM=KENWARDROGER ;
  REPEATED VISIT / TYPE=UN  SUB=SUBJID  GROUP=TREAT;
  LSMEANS TREAT*VISIT/PDIFF=CONTROLU('1'  '13') ALPHA=0.2;
RUN;
```

Note: the SAS code could be modified due to data limitation and convergence issue.

6.10.3 Sensitivity Analysis

No sensitivity analysis is planned for the study.

6.10.4 Exploratory Analysis

Exploratory analyses will be performed to evaluate the impact of baseline covariates on the primary efficacy variables. Such analyses may be described in the clinical study report.

- A re-analysis of the primary endpoint using the MMRM model and excluding all patients from sites that recruited 2 or fewer patients.
- Last observation carried forward (LOCF) analysis: Missing SIB assessment at Week 13 will be imputed using the LOCF method for subjects with post-baseline assessments. An analysis of covariance (ANCOVA) for the Week 13 Visit will be conducted. The model will include treatment, and baseline SIB as a covariate.
- Site will be added as an additional covariate in above ANCOVA to assess site effect.
- Use of AChEI, Memantine at baseline will be added as an additional covariate in above ANCOVA to assess site effect.
- Subjects will be divided into two subgroups: 1) baseline SIB<=median baseline SIB, 2) baseline SIB>median baseline SIB. An analysis of covariance (ANCOVA) for the Week 13 Visit will be conducted to assess treatment difference (low dose vs. placebo, high dose vs. placebo). The model will include treatment, and baseline SIB as a covariate. Missing SIB assessment at Week 13 will be imputed using the LOCF method for subjects with post-baseline assessments.
- The Following responder analyses at week 13 will be conducted to compare the proportions of responders between treatment groups (low dose vs. placebo, high dose vs. placebo, pooled dose vs. placebo) using CMH approach. Missing assessment at Week 13 will be imputed using the LOCF method for subjects with post-baseline assessments.
  A. A responder is defined as a subject who had improvement on the SIB.
  B. A responder is defined as a subject who had improvement of at least 3.9 points on the SIB.
  C. A responder is defined as a subject who had improvement of at least 6.5 points on the SIB.
  D. A responder is defined as a subject who had improvement either on SIB or on ADCS-ADL-SIV.

The same analyses will be performed for pooled dose vs. placebo.

6.10.5 Secondary Endpoints

Secondary endpoints for ADCS-ADL-SIV, MMSE-2 (excludes MMSE Stratum variable), NPI at week 13 will be analyzed using a statistical model similar to the one used for analysis of the SIB. The CGI-I secondary endpoint will be analyzed in similar fashion except the model will not have a baseline value used as a covariate.

The secondary efficacy endpoints will not be formally tested against the null hypothesis; however, p-values and CI will be used to support the results of the primary endpoint analysis.

6.10.6 Exploratory Endpoints

The exploratory PK endpoints will be addressed in a separate analysis plan.

The following exploratory endpoints will be analyzed using the same statistical model as the primary endpoint:

- Change from baseline in SIB and MMSE Scores at the 30-day follow up
- Change from Week 13 in SIB and MMSE Scores at the 30-day follow up.

Subjects who are randomized at treatment period 2 will be excluded at the 30-day follow up.

ANCOVA model described in section 6.10.4 will be used for SIB, ADCS-ADL, NPI, and MMSE-2 by visit.

ANCOVA model will be used for ADCS-ADL, NPI, and MMSE-2 at Week 13. Missing assessment at Week 13 will be imputed using the LOCF method for subjects with post-baseline assessments.

Subjects will be divided into two subgroups: 1) baseline ADCS-ADL <= median baseline ADCS-ADL, 2) baseline ADCS-ADL > median baseline ADCS-ADL. An analysis of covariance (ANCOVA) for the Week 13 Visit will be conducted to assess treatment difference (low dose vs. placebo, high dose vs. placebo). The model will include treatment, and baseline ADCS-ADL as a covariate. Missing ADCS-ADL assessment at Week 13 will be imputed using the LOCF method for subjects with post-baseline assessments. The same analyses for NPI, and MMSE-2 at Week 13 will also be conducted.

The effects of center and treatment by center interaction will be explored using an analysis of covariance (ANCOVA) model as describe in section 6.10.4 if data permit. Low enrolling centers may be pooled if appropriate. Stepwise covariates selection approach may be applied. These will explored as post hoc analyses.

6.10.7 Multiplicity

All secondary endpoints and the supportive analyses will be considered as descriptive evidence of efficacy and will be analyzed without any procedures to account for multiple comparisons.

6.11 Pharmacodynamic Analyses

PKCε concentration prior to the infusion, at the end of infusion (+5minute window) and 60 minutes after completion of infusion (±5minutes) will be collected from selected sites for doses

1, 3, and 7, respectively. Descriptive summary will covered by WCT Bioanalytical Sciences Group.

6.12 Safety Analyses

The safety analyses will be presented by the actual treatment received for the SA Set.

6.12.1 Adverse Events

A TEAE in the main treatment period is defined as follows:

- Any AE with an onset on or after the first dose of study drug and through 30 days after the last dose of study drug in the main treatment period.
- Any pre-existing AE that has worsened in severity on or after the first dose of study drug and through 30 days after the last dose of study drug in the main treatment period.

A TEAE in treatment period 2 is defined as follows:

- Any AE that has an onset on or after the first dose of treatment period 2 at the Week 13 Visit

A treatment-related AE is defined as an AE as being *possibly or probably* related to the study drug. If an AE has a missing relationship, it is assumed to be related to the study drug for analysis purposes.

Maximum severity will be assumed for an AE with missing severity.

The following tables will be presented for AEs in the main treatment period:

- Overall incidence and the number of AEs, SAEs, treatment emergent adverse events of special interest (AESIs)-myalgia, TEAEs leading to withdrawal of study drug.
- TEAE by system organ class and preferred term, incidence and number of events
- Treatment related TEAE by system organ class and preferred term, incidence and number of events
- Serious TEAE by system organ class and preferred term, incidence and number of events
- TEAESI-myalgia by system organ class and preferred term, incidence and number of events
- TEAEs leading to withdrawal of study drug by system organ class and preferred term, incidence and number of events
- TEAE by system organ class, preferred term and maximum severity, incidence
- Listing of serious TEAEs (presented in the Table section of the appendices)

- Listing of deaths (presented in the Table section of the appendices)
- Listing of relationship of AE verbatim term with MedDRA

Adverse events will be coded using MedDRA version 18.1.

A complete subject listing of all AEs will be provided. This listing will include treatment, AE verbatim term, primary system organ class and preferred term, the time of onset and cessation of event relative to the first dose of study medication, duration of AE (for ongoing AEs, no duration will be calculated), whether serious, severity, relationship to study medication, action taken and outcome.

6.12.2 Laboratory Data

Descriptive statistics of the observed values and change from baseline (continuous data) will be presented by treatment group and visit for each hematology, coagulation, CBC with differential, and serum chemistry parameter. Each measurement (continuous data) will be classed as below, within, or above normal range, based on ranges supplied by the laboratory used. Shift tables in relation to the normal range from baseline to each follow-up visit will be presented.

Liver Function Parameter Abnormalities

The number and percentage of subjects meeting certain liver function abnormality categories will be summarized by visit and any post-baseline visit (e.g., post-dose) during the treatment period:

- Alanine Aminotransferase (AST) assessment > 3 times upper limit of normal (ULN),
- Aspartate aminotransferase (AST) > 3xULN,
- ALT or AST: > 3xULN; > 10xULN; > 20xULN
- Total Bilirubin: > 1.5xULN; > 2xULN
- Alkaline Phosphatase: > 1.5xULN; >2xULN
- (ALT or AST > 3xULN) and total bilirubin>2xULN
- (ALT or AST>3xULN) and total bilirubin>2xULN and alkaline phosphatase<2xULN

Box plots for the above lab parameters will be provided for changes from baseline and maximal change. In addition, Scatter Plots of LFT Parameters Post-Baseline vs Baseline.

Lab Parameter Shift Tables

Shift tables in relation to the normal range from screening to each follow-up visit will be presented for Hematology and Chemistry. In addition, Shifts from baseline will be evaluated for

following Lab parameters based on Common Terminology Criteria for Adverse Events (CTCAE 4.03, 2010-06-14) grade. The baseline and highest post-dose results for each of these parameters will be categorized by CTCAE grade. The number and percentage of subjects in each baseline by post-baseline category will be summarized by analysis category. Separate shift tables will be prepared for the highest post-dose assessment and one for the last post-dose assessment during the treatment period. This will include all scheduled and unscheduled laboratory assessments.

A table displaying the CTCAE grades for lab parameters of interest is provided below:

Parameter (unit)	Grade 1	Grade 2	Grade 3	Grade 4
APTT (seconds)	>ULN-1.5xULN	>1.5xULN-2.5xULN	> 2.5xULN	----
Alkaline phosphate (U/L)	>ULN-2.5xULN	>2.5xULN-5xULN	>5xULN-20xULN	>20xULN
ALT (U/L)	>ULN-3xULN	>3xULN-5xULN	>5xULN-20xULN	>20xULN
AST (U/L)	>ULN-3xULN	>3xULN-5xULN	>5xULN-20xULN	>20xULN
Total bilirubin (µmol/L)	>ULN-1.5xULN	>1.5xULN-3xULN	>3xULN-10xULN	>10xULN
Creatinine	> 1-1.5x baseline or > ULN-1.5xULN	>1.5-3.0 x baseline or > 1.5xULN-3.0xULN	> 3.0- 6.0 x baseline or >3xULN-6xULN	>6x baseline or > 6xULN
Creatinine clearance	60<=RESULT<LLN ml/min	59-30 ml/min	29-15 ml/min	<15 ml/min
WBC decreased	<LLN - 3000/mm3; <LLN - 3.0 x 10e9 /L	<3000 - 2000/mm3; <3.0 - 2.0 x 10e9 /L	<2000 - 1000/mm3; <2.0 - 1.0 x 10e9 /L	<1000/mm3; <1.0 x 10e9 /L
PLT (PLAT/Platelet count)	<LLN - 75,000/mm3; <LLN - 75.0 x 10e9 /L	<75,000 - 50,000/mm3; <75.0 - 50.0 x 10e9 /L	<50,000 - 25,000/mm3; <50.0 - 25.0 x 10e9 /L	<25,000/mm3; <25.0 x 10e9 /L
Hemoglobin (anemia)	Hemoglobin (Hgb) <LLN - 10.0 g/dL; <LLN - 6.2 mmol/L; <LLN - 100 g/L	Hemoglobin (Hgb) <LLN 10.0 - 8.0 g/dL;<br><LLN 6.2 - 4.9 mmol/L;<br><LLN 100 – 80 g/L	Hgb <8.0 g/dL; <4.9 mmol/L; <80 g/L	-
INR	>1 - 1.5 x ULN; >1 - 1.5 times above baseline if on anticoagulation	>1.5 - 2.5 x ULN; >1.5 - 2.5 times above baseline if on anticoagulation	>2.5 x ULN; >2.5 times above baseline if on anticoagulation	-
CPK/CK (increase)	>ULN - 2.5 x ULN	>2.5 x ULN - 5 x ULN	>5 x ULN - 10 x ULN	>10 x ULN

6.12.3 Vital Signs

Descriptive statistics for observed values and changes from baseline in the following vital signs will be presented by treatment group and visit:

- Systolic blood pressure (mmHg)
- Diastolic blood pressure (mmHg)
- Pulse rate (bpm)
- Respiration rate (breaths/min)
- Body temperature (degrees Celsius)

Vital sign values will be categorized into clinical concern (CS) categories if applicable.

Vital Sign	PCS categories
Systolic blood pressure	120-139; 140-159; ≥160 mmHg
Diastolic blood pressure	80-89; 90-99; ≥ 100 mmHg
Pulse rate	<60 or >100 bpm
Respiratory Rate	> 27 breaths/min

Number and percent of subjects falling into these categories will be summarized by Visit and Any time, post-baseline during the treatment period. For scheduled visits (excluding Week 13 Visit) where multiple assessments are collected (pre and post infusion), worst case will be counted for that visit.

Box plots for the above vital signs will be provided for changes from baseline by visit and maximal change.

Shifts from baseline to worst for hyper and hypotension for CTCAE will be evaluated for based on Common Terminology Criteria for Adverse Events (CTCAE 4.03, 2010-06-14) grade. The baseline and highest post-dose results for each of these blood pressure will be categorized by CTCAE grade. Separate shift tables will be prepared for the highest post-dose assessment and one for the last post-dose assessment during the treatment period. This will include all scheduled and unscheduled vital assessments.

A table displaying the CTCAE grades for hyper and hypotension is provided below:

Parameter (unit)	Grade 1	Grade 2	Grade 3	Grade 4
Systolic BP (mmHg)	120 - 139 mmHg	140 - 159 mmHg	>=160 mm Hg	-
Diastolic BP (mmHg)	80 - 89 mmHg	90 - 99 mmHg	>=100 mm Hg	-

6.12.4 Electrocardiogram Data

Descriptive statistics for observed values and changes from baseline in the following ECG variables will be tabulated at each post-baseline visit:

- Heart rate (bpm)
- PR interval (ms)
- RR interval (ms)
- QT interval (ms)
- QRS Duration, (ms)
- QTcF interval (ms) [Fridericia's formula - QTcF]

ECG Result Interpretation (Normal, Abnormal Not Clinically Significant [NCS], and Abnormal Clinically Significant [CS]) will be summarized by treatment group and visit.

The incidence of ECG abnormalities for subjects with any abnormal CS ECG result will be presented by treatment group and visit.

QTcF values will be categorized into the following categories:

- < 450 ms
- 450-480 ms
- 481-500 ms
- > 500 ms

Number and percent of subjects that fall into these categories will be presented by treatment group and visit. Box plots for ECG data will be provided for changes from baseline by visit and maximal change.

6.12.5 Physical Examination

The body systems within the physical examination (PE) data will be summarized by treatment group and visit (Normal; Abnormal NCS, Abnormal CS). Changes from baseline will also be tabulated. Details of CS findings will be listed.

Descriptive statistics for observed values and changes from baseline in weight will be presented by treatment group and visit. Number and percentage of subjects with > 10% change from baseline (increase or decrease) will also be summarized.

6.12.6 Second Treatment Period Analyses

AEs (i.e. an AE has an onset on or after the first dose of the second treatment period or a pre-existing AE that has worsened in severity on or after the first dose of the second treatment period.) will be summarized by treatment.

The following tables will be presented for AEs in the second treatment period:

- Overall incidence and the number of AEs, SAEs, treatment emergent adverse events of special interest (AESIs)-myalgia, TEAEs leading to withdrawal of study drug, Period 2
- AE by system organ class and preferred term, incidence and number of events, Period 2

7 INTERIM ANALYSIS

No formal interim analyses are planned.

8 DATA SAFETY MONITORING BOARD ANALYSIS

Data safety monitoring board (DSMB) analyses/summaries are described separate from this document.

9 CHANGES TO PLANNED PROTOCOL ANALYSIS

10 REFERENCES

1. SAS Institute Inc. The SAS System, Version 9.3. Cary, NC, SAS Institute Inc. 2012.

11 LIST OF TABLES, FIGURES AND LISTINGS

The following table includes details of the tables, figures and listings to be included within each section of the eCTD. The eCTD section is shown in bold. The following validation methods maybe used

- Independent programming of numbers and manual review of format (IP)
- Independent programming by statistician of numbers and manual review of format (Stat IP)
- Manual review (MR)
- Code review (CR)

Table Number	Table Title	Validation Method	Shell Number (If Repeat)
**14.1**	**Demographics Data**		
**14.1.1**	**Disposition**		
14.1.1.1	Subject Disposition, Analysis Sets<br>Enrolled Set		
14.1.1.2	Subject Disposition, Second 12-Week Treatment Phase		
14.1.1.3	Subject Disposition, Screen Failures<br>Enrolled Set		
14.1.1.4	Inclusion/Exclusion Criteria not met<br>Enrolled Set		
14.1.1.5	Subject Disposition, Early Withdrawals<br>Safety Analysis Set		
14.1.1.6	Subject Disposition, Early Withdrawals<br>Full Analysis Set		
14.1.1.7	Subject Disposition, Safety Analysis Set by Site<br>Safety Analysis Set		
14.1.1.8	Subject Disposition, Safety Analysis Set by Site<br>Full Analysis Set		
14.1.1.9	Subject Disposition, Visits<br>Safety Analysis Set		
14.1.1.10	Subject Disposition, Visits		

	Full Analysis Set		
**14.1.2**	**Demographics**		
14.1.2.1	Demographics<br>Safety Analysis Set		
14.1.2.2	Demographics<br>Full Analysis Set		
14.1.2.3	Demographics<br>Completer Analysis Set		
14.1.2.4	Baseline Characteristics<br>Safety Analysis Set		
14.1.2.5	Baseline Characteristics<br>Full Analysis Set		
14.1.2.6	Baseline Characteristics<br>Completer Analysis Set		
**14.1.3**	**Baseline Characteristics**		
14.1.3.1	Prior Medical History<br>Safety Analysis Set		
14.1.3.2	Prior Medical History<br>Full Analysis Set		
14.1.3.3	Ongoing Medical Conditions at Screening<br>Safety Analysis Set		
14.1.3.4	Ongoing Medical Conditions at Screening<br>Full Analysis Set		
14.1.3.5	Prior Medications<br>Safety Analysis Set		
14.1.3.6	Prior Medications<br>Full Analysis Set		
14.1.3.7	Major Deviations<br>Full Analysis Set		
**14.2**	**Efficacy Data**		
**14.2.1**	**Primary Efficacy Endpoint**		
14.2.1.1	Severe Impairment Battery (SIB) Scale<br>Full Analysis Set		
14.2.1.2	Severe Impairment Battery (SIB) Scale<br>Completer Analysis Set		

14.2.1.3	Mixed Models for Repeated Measures Analysis of Change from Baseline in SIB Total Score<br>Full Analysis Set		
14.2.1.4	Mixed Models for Repeated Measures Analysis of Change from Baseline in SIB Total Score<br>Completer Analysis Set		
14.2.2.1	Alzheimer Disease Cooperative Study Activities of Daily Living Inventory Severe Impairment Version (ADCS-ADL-SIV)<br>Full Analysis Set		
14.2.2.2	Mixed Models for Repeated Measures Analysis of Change from Baseline in ADCS-ADL-SIV Total Score<br>Full Analysis Set		
14.2.2.3	Mini Mental State Examination Version 2 (MMSE-2)<br>Full Analysis Set		
14.2.2.4	Mixed Models for Repeated Measures Analysis of Change from Baseline in MMSE-2 Total Score<br>Full Analysis Set		
14.2.2.5	Neuropsychiatric Inventory (NPI) Scale<br>Full Analysis Set		
14.2.2.6	Mixed Models for Repeated Measures Analysis of Change from Baseline in NPI total Score<br>Full Analysis Set		
14.2.2.7	Clinical Global Impression of Improvement (CGI-I)<br>Full Analysis Set		
14.2.2.8	Mixed Models for Repeated Measures Analysis of CGI-I Score<br>Full Analysis Set		
**14.2.3**	**Exploratory Endpoints**		

14.2.3.1.1	Analysis of Covariance of Change from Baseline in SIB Total Score<br>Full Analysis Set		
14.2.3.1.2	Analysis of Covariance of Change from Baseline in SIB Total Score to Assess Site Effect<br>Full Analysis Set		
14.2.3.1.3	Analysis of Covariance of Change from Baseline in SIB Total Score of Use of AChEI or Memantine at Baseline as an Additional Covariate<br>Full Analysis Set		
14.2.3.1.4a	Analysis of Covariance of Change from Baseline in SIB Total Score, <= Median Baseline SIB Total Score<br>Full Analysis Set		
14.2.3.1.4b	Analysis of Covariance of Change from Baseline in SIB Total Score, > Median Baseline SIB Total Score<br>Full Analysis Set		
14.2.3.1.5a	A Responder Analysis (CMH) of Subject Who Had Improvement on SIB Total Score at Week 13<br>Full Analysis Set		
14.2.3.1.5b	A Responder Analysis (CMH) of Subject Who Had Improvement of at Least 3.9 Points on SIB Total Score at Week 13<br>Full Analysis Set		
14.2.3.1.5c	A Responder Analysis (CMH) of Subject Who Had Improvement of at Least 6.5 Points on SIB Total Score at Week 13<br>Full Analysis Set		
14.2.3.1.6	Mixed Models for Repeated Measures Analysis of Change from Baseline in SIB Total Score Excluding All Patients from Sites that Recruited 2 or Fewer Patients<br>Full Analysis Set		

14.2.3.1.7	Mixed Models for Repeated Measures Analysis of Changes from Baseline and Week 13 in SIB Total Score at 30-day Follow up<br>Full Analysis Set		
14.2.3.2.1	A Responder Analysis (CMH) of Subjects Who Had Improvement either on SIB or on ADCS-ADL-SIV<br>Full Analysis Set		
14.2.3.2.2	Analysis of Covariance of Change from Baseline in ADCS-ADL-SIV Total Score<br>Full Analysis Set		
14.2.3.2.3	Analysis of Covariance of Change from Baseline in ADCS-ADL-SIV Total Score to Assess Site Effect<br>Full Analysis Set		
14.2.3.2.4a	Analysis of Covariance of Change from Baseline in ADCS-ADL-SIV Total Score, <= Median Baseline ADCS-ADL-SIV Total Score<br>Full Analysis Set		
14.2.3.2.4b	Analysis of Covariance of Change from Baseline in ADCS-ADL-SIV Total Score, > Median Baseline ADCS-ADL-SIV Total Score<br>Full Analysis Set		
14.2.3.2.5	Analysis of Covariance of Change from Baseline in MMSE-2 Total Score<br>Full Analysis Set		
14.2.3.2.6	Analysis of Covariance of Change from Baseline in MMSE-2 Total Score to Assess Site Effect<br>Full Analysis Set		
14.2.3.2.7a	Analysis of Covariance of Change from Baseline in MMSE-2 Total Score, <= Median Baseline MMSE-2 Total Score<br>Full Analysis Set		

14.2.3.2.7b	Analysis of Covariance of Change from Baseline in MMSE-2 Total Score, > Median Baseline MMSE-2 Total Score<br>Full Analysis Set		
14.2.3.2.8	Analysis of Covariance of Change from Baseline in NPI Total Score<br>Full Analysis Set		
14.2.3.2.9	Analysis of Covariance of Change from Baseline in NPI Total Score to Assess Site Effect<br>Full Analysis Set		
14.2.3.2.10a	Analysis of Covariance of Change from Baseline in NPI Total Score, > Median Baseline NPI Total Score<br>Full Analysis Set		
14.2.3.2.10b	Analysis of Covariance of Change from Baseline in NPI Total Score, <= Median Baseline NPI Total Score<br>Full Analysis Set		
**14.3**	**Safety Data**		
**14.3.1**	**Displays of Adverse Events**		
14.3.1.1	Adverse Events, Overview of Treatment-Emergent Adverse Events (TEAE)<br>Safety Analysis Set		
14.3.1.2	Adverse Events, TEAEs by Primary System Organ Class and Preferred Term<br>Safety Analysis Set		
14.3.1.3	Adverse Events, Treatment-Related TEAEs by Primary System Organ Class and Preferred Term<br>Safety Analysis Set		
14.3.1.4	Adverse Events, TEAEs leading to Study Drug Discontinuation by Primary System Organ Class and Preferred Term<br>Safety Analysis Set		
14.3.1.5	Adverse Events, Serious TEAEs by Primary System Organ Class and Preferred Term		

	Safety Analysis Set		
14.3.1.6	Adverse Events, Treatment-Related Serious TEAEs by Primary System Organ Class and Preferred Term<br>Safety Analysis Set		
14.3.1.7	Adverse Events, TEAEs of Special Interest - Myalgia<br>Safety Analysis Set		
14.3.1.8	Adverse Events, TEAEs by Primary System Organ Class, Preferred Term and Maximum Severity<br>Safety Analysis Set		
14.3.1.9	Adverse Events, Overview of Treatment-Emergent Adverse Events (TEAE), Period 2<br>Safety Analysis Set		
14.3.1.10	Adverse Events, TEAEs by Primary System Organ Class and Preferred Term, Period 2<br>Safety Analysis Set		
**14.3.2**	**Listings Of Deaths, Other Serious And Significant Adverse Events**		
14.3.2.1	Deaths, Listing<br>Enrolled Set		
14.3.2.2	SAE, Listing<br>Safety Analysis Set		
14.3.2.3	Treatment-Related TEAEs, Listing<br>Safety Analysis Set		
14.3.2.4	TEAEs leading to Study Drug Discontinuation, Listing<br>Safety Analysis Set		
14.3.2.5	TEAEs of Special Interest – Myalgia, Listing<br>Safety Analysis Set		
**14.3.3**	**Narratives of Deaths, Other Serious and Certain Other Significant Adverse Events**<br>*(This is usually not created by stats but this placeholder)*		
**14.3.4**	**Abnormal Laboratory Values**		

14.3.4.1	Hematology<br>Safety Analysis Set		
14.3.4.2	Shift Table-Hematology<br>Safety Analysis Set		
14.3.4.3	Chemistry<br>Safety Analysis Set		
14.3.4.4	Shift Table-Chemistry<br>Safety Analysis Set		
14.3.4.5	Liver Function Abnormalities<br>Safety Analysis Set		
14.3.4.6	Shift for Selected Lab Parameters from Baseline to Last Assessment Based on CTCAE Grade<br>Safety Analysis Set		
14.3.4.7	Shift for Selected Lab Parameters from Baseline to Highest Post-Baseline Value based on CTCAE Grade<br>Safety Analysis Set		
**14.3.5**	**Extent Of Exposure, Dosage Information, And Compliance**		
14.3.5.1	Infusions<br>Safety Analysis Set		
**14.3.6**	**Vital Signs And Physical Examination**		
14.3.6.1	Vital Signs<br>Safety Analysis Set		
14.3.6.2.1	Vital Sign Data outside Clinical Concern Range<br>Safety Analysis Set		
14.3.6.2.2	Shift for Systolic BP and Diastolic BP from Baseline to Worst CTCAE Grade<br>Safety Analysis Set		
14.3.6.3	Physical Examinations<br>Safety Analysis Set		
14.3.6.4	Physical Examinations, Changes from Baseline<br>Safety Analysis Set		
14.3.6.5	Physical Examination-Weight		

	Safety Analysis Set		
14.3.6.6	Physical Examination-Weight Data outside Clinical Concern Range<br>Safety Analysis Set		
**14.3.7**	**Other Safety**		
14.3.7.1	ECGs<br>Safety Analysis Set		
14.3.7.2	ECG Result Interpretations<br>Safety Analysis Set		
14.3.7.3	ECG Abnormalities for Subjects with Any Abnormal Clinically Significant ECG Interpretation<br>Safety Analysis Set		
14.3.7.4	QTcF Categories<br>Safety Analysis Set		
14.3.7.5	Columbia-Suicide Severity Rating Scale (C-SSRS) at Screening<br>Safety Analysis Set		
14.3.7.6	Columbia-Suicide Severity Rating Scale (C-SSRS) at Post-baseline Visits<br>Safety Analysis Set		
**14.3.8**	**Concomitant Medication**		
14.3.8.1	Concomitant Medications<br>Safety Analysis Set		

Figure Number	Figure Title	Validation Method	Shell Number (if repeat)
14.2.1	Mean (+/- SE) Change from baseline in Severe Impairment Battery (SIB) Over Time<br>Full Analysis Set		
14.3.1.1	Box Plot of Change from Baseline in Heart Rate (beats/min)<br>Safety Analysis Set		
14.3.1.2	Box Plot of Change from Baseline in PR (msec)		

Figure Number	Figure Title	Validation Method	Shell Number (if repeat)
	Safety Analysis Set		
14.3.1.3	Box Plot of Change from Baseline in RR (msec)<br>Safety Analysis Set		
14.3.1.4	Box Plot of Change from Baseline in QT (msec)<br>Safety Analysis Set		
14.3.1.5	Box Plot of Change from Baseline in QRS (msec)<br>Safety Analysis Set		
14.3.1.6	Box Plot of Change from Baseline in QTcF (msec)<br>Safety Analysis Set		
14.3.2.1	Box Plot of Change from Baseline in Systolic Blood Pressure (mmHg)<br>Safety Analysis Set		
14.3.2.2	Box Plot of Change from Baseline in Diastolic Blood Pressure (mmHg)<br>Safety Analysis Set		
14.3.2.3	Box Plot of Change from Baseline in Pulse Rate (beats/min)<br>Safety Analysis Set		
14.3.2.4	Box Plot of Change from Baseline in Respiration (breaths/min)<br>Safety Analysis Set		
14.3.2.5	Box Plot of Change from Baseline in Weight (kg)<br>Safety Analysis Set		
14.3.3.1.1	Box Plot of Alanine Aminotransferase (ALT) with an ULN Range<br>Safety Analysis Set		
14.3.3.1.2	Box Plot of Alanine Aminotransferase (ALT) with an LLN Range<br>Safety Analysis Set		

Figure Number	Figure Title	Validation Method	Shell Number (if repeat)
14.3.3.2.1	Box Plot of Aspartate aminotransferase (AST) with an ULN Range Safety Analysis Set		
14.3.3.2.2	Box Plot of Aspartate aminotransferase (AST) with an LLN Range Safety Analysis Set		
14.3.3.3.1	Box Plot of Total Bilirubin with an ULN Range Safety Analysis Set		
14.3.3.3.2	Box Plot of Total Bilirubin with an LLN Range Safety Analysis Set		
14.3.3.4.1	Box Plot of Alkaline Phosphatase with an ULN Range Safety Analysis Set		
14.3.3.4.2	Box Plot of Alkaline Phosphatase with an LLN Range Safety Analysis Set		
14.3.3.5	Box Plot of Creatinine Clearance with an LLN Range Safety Analysis Set		
14.3.4.1	Scatter Plots of LFT Parameters Post-Baseline versus Baseline with ULN Range Safety Analysis Set		
14.3.4.2	Scatter Plot of Maximum ALT versus Maximum Total Bilirubin Safety Analysis Set		
14.3.4.3	Scatter Plot of Maximum AST versus Maximum Total Bilirubin Safety Analysis Set		
14.3.5	Box plot of Baseline CPK of All Safety Subjects versus Those with at Least 1 Myalgia Event Safety Analysis Set		

Listing Number	Listing Title	Validation Method	Shell Number (if repeat)
**16.2**	**Subject Data Listings**		
**16.2.1**	**Discontinued Subjects**		
16.2.1.1	Subject Disposition		
16.2.1.2	Screen Failures		
16.2.1.3	Early Withdrawals		
**16.2.2**	**Protocol Deviations**		
16.2.2.1	Protocol Deviations		
**16.2.3**	**Subjects Excluded From The Efficacy Analyses**		
16.2.3.1	Major Protocol Deviations		
**16.2.4**	**Demographic Data**		
16.2.4.1.1	Subject Randomization		
16.2.4.1.2	Demographic Data		
16.2.4.2.1	Medical History-Probable Alzheimer's Diagnosis		
16.2.4.2.2	Rosen-Modified Hachinski Ischemic Scale and Total Score		
16.2.4.3.1	Previous Medical History		
16.2.4.3.2	Ongoing Medical History		
16.2.4.4	Inclusion Criteria Not Met		
16.2.4.5	Exclusion Criteria Not Met		
16.2.4.6	Prior and Concomitant Medications		
**16.2.5**	**Compliance And/Or Drug Concentration Data**		
16.2.5.1	Infusions and Compliance		
**16.2.6**	**Individual Efficacy Response Data**		
16.2.6.1.1	Severe Impairment Battery (SIB) Scale, Items 1 – 20		
16.2.6.1.2	SIB Scale, Items 21 – 40 and Total Score		
16.2.6.2	Alzheimer Disease Cooperative Study Activities of Daily Living Inventory Severe Impairment Version (ADCS-ADL-SIV) and Total Score		
16.2.6.3	Mini Mental State Examination Version 2 (MMSE-2) and Total Score		
16.2.6.4	Clinical Global Impression of Improvement (CGI-I)		
16.2.6.5	Neuropsychiatric Inventory (NPI) Scale		
**16.2.7**	**Adverse Event Listings**		

Listing Number	Listing Title	Validation Method	Shell Number (if repeat)
16.2.7.1	All Adverse Events		
16.2.7.2	All Serious Adverse Events		
**16.2.8**	**Individual Laboratory Measurements And Other Safety**		
16.2.8.1.1	Hematology Results		
16.2.8.1.2	Chemistry Results		
16.2.8.1.3	Laboratory Liver Function Parameter Abnormalities		
16.2.8.1.4	Other Laboratory Tests		
16.2.8.2	Vital Signs		
16.2.8.3	ECG Parameters		
16.2.8.4	Physical Examination, Abnormal Results		
16.2.8.5	Columbia-Suicide Severity Rating Scale (C-SSRS)		

Statistical Analysis Plan

Title: A Randomized, Double-Blind, Placebo-Controlled, Phase 2 Study Assessing the Safety, Tolerability and Efficacy of Bryostatin in the Treatment of Moderately Severe to Severe Alzheimer's Disease

Protocol Number: NTRP101-202

Version Final

Issue Date: 22-Mar-2017

Author: Shuhua Qi, MSc

Previous Versions

SAP Amendments before database lock

Version	Issue Date	Section	Revision/Addition	Rationale
Final	22Mar2017	6.1.6	Added Complete analysis set	Sponsor request
Final	22Mar2017	11	Added 4 Tables for Complete set and modified few table number accordingly	Sponsor request/agreement
Final	17Mar2017	6.10.2	Updated SAS code	Sponsor agreement
Final	14Mar2017	Page 1	Updated protocol version	Protocol amendment 4
Final	14Mar2017	6.10.2, 6.10.4, 6.10.6	1) Re-organizes.<br>2) Added following sentence in section 6.10.6:<br>The effects of center and treatment by center interaction will be explored using an analysis of covariance (ANCOVA) model as describe in section 6.10.4 if data permit. Low enrolling centers may be pooled if appropriate. Stepwise covariates selection approach may be applied. These will explored as post hoc analyses.<br>3) the followings were removed<br>- Correlation of change in (SIB, ACDC-ADL, NPI, etc) with bryostatin Cmax and AUC	Sponsor request/agreement

			- Correlation of change in (SIB ACDC-ADL, NPI, etc) with mean change in serum PKCepsilon levels	
Draft v4	14Feb2017	3.3	Removed percent from baseline in SIB score	Not in protocol
		6.1.4	Removed PPS	Sponsor request
		6.2.8.1	Replaced by original/draft1 SAP wording for calculation of dose taken and percentage of planned dose taken	The analysis can/will take place after unblinding per sponsor request
		6.10.1, 6.10.2	Revised primary analysis method	Protocol amendment XXX
		6.10.3	Sensitivity analysis was not planned	Sponsor request
		6.10.4, 6.10.5 and 6.10.6	Revised/added exploratory analysis methods	Sponsor request
		6.12.3	Added vital BP based on CTCAE grade	Sponsor request
Draft v3	02Feb2017	6.11	WCT PK group will proved summary table/listings for PKCε concentration	WCT PK group agreement
		Last page	Approval for implementation of Statistical Analysis Plan	
Draft v2	27Dec2016	Cover page	Changed author	Transition,12Dec2016
		Original 6.2.20, 6.11	Removed section 6.2.20 Calculation of Area under the Curve and section 6.11 Pharmacokinetic Analyses	A separate PK data analysis plan (PAP).

		2.8.1	Revised Calculation of dose taken and percentage of planned dose taken	Sponsor agreement
		6.12.2, 6.12.3 & 6.12.3	Added figures for Lab, vital signs and ECG	Sponsor request

Table of Contents

1 INTRODUCTION 8
2 STUDY OBJECTIVES 8
3 ENDPOINTS 9
3.1 Primary Endpoint 9
3.1.1 Safety 9
3.1.2 Efficacy 9
3.2 Secondary Endpoints 9
3.2.1 Safety 9
3.2.2 Efficacy 9
4 SAMPLE SIZE 10
5 RANDOMIZATION 10
6 PLANNED ANALYSES 10
6.1 Analysis Sets 11
6.1.1 Enrolled Set 11
6.1.2 Randomized Set 11
6.1.3 Full Analysis Set 11
6.1.4 Per-Protocol Set 11
6.1.5 Safety Analysis Set 11
6.1.6 Complete Analysis Set 11
6.2 Derived Data 11
6.2.1 Age 12
6.2.2 Screening 12
6.2.3 Baseline and Change from Baseline 12
6.2.4 Duration/Study Day/Time 12
6.2.5 Conventions for Missing and Partial Dates 12
6.2.6 Missing/Partial Start/Stop Date of Adverse Events and Concomitant Medications 12
6.2.7 Missing Diagnosis Dates 14
6.2.8 Exposure to Study Drug 14
6.2.9 Inexact Values 15
6.2.10 Study Period/Analysis Windows 15

6.2.11 Diagnosis of AD 16
6.2.12 Severe Impairment Battery (SIB) 16
6.2.13 Alzheimer Disease Cooperative Study Activities of Daily Living Inventory-Severe Impairment Version (ADCS-ADL-SIV) 17
6.2.14 Mini Mental State Examination 2 (MMSE-2) 17
6.2.15 Neuropsychiatric Inventory (NPI) 18
6.2.16 Clinical Global Impression of Improvement (CGI-I) 19
6.2.17 Rosen-Modified Hachinski Scale 19
6.2.18 Electrocardiogram Data 19
6.2.19 Columbia-Suicide Severity Rating Scale 19
6.2.20 Unscheduled Visits 20
6.3 Conventions 20
6.3.1 Decimal Places 21
6.4 Subject Disposition 22
6.5 Protocol Deviations 22
6.6 Baseline Comparability 22
6.7 Medical History 23
6.8 Prior and Concomitant Medications 23
6.9 Exposure to Study Drug/Treatment compliance 24
6.10 Efficacy Analyses 24
6.10.1 Primary Endpoint 24
6.10.2 Primary Efficacy Analysis 25
6.10.3 Sensitivity Analysis 25
6.10.4 Exploratory Analysis 26
6.10.5 Secondary Endpoints 26
6.10.6 Exploratory Endpoints 27
6.10.7 Multiplicity 27
6.11 Pharmacodynamic Analyses 27
6.12 Safety Analyses 28
6.12.1 Adverse Events 28
6.12.2 Laboratory Data 29
6.12.3 Vital Signs 30
6.12.4 Electrocardiogram Data 32
6.12.5 Physical Examination 32

6.12.6 Second Treatment Period Analyses 32
7 INTERIM ANALYSIS 33
8 DATA SAFETY MONITORING BOARD ANALYSIS 33
9 CHANGES TO PLANNED PROTOCOL ANALYSIS 33
10 REFERENCES 34
11 LIST OF TABLES, FIGURES AND LISTINGS 35

1 INTRODUCTION

This document details the planned statistical analyses of non-pharmacokinetic (PK) data for Neurotrope BioScience , protocol “NTRP101-202” study titled “A Randomized, Double-Blind, Placebo-Controlled, Phase 2 Study Assessing the Safety, Tolerability and Efficacy of Bryostatin in the Treatment of Moderately Severe to Severe Alzheimer’s Disease”.

The proposed analyses are based on the contents of the final version of the protocol (dated 17-Apr-2015) and amendments 2.0, 3.0 and 4.0 (dated 20-Jan-2016, 15-Jul-2016 and 03-Mar-2017, respectively).

This is a randomized double-blind placebo-controlled Phase 2 study comparing different doses of bryostatin for the treatment of moderately severe to severe Alzheimer’s disease (AD). The study was originally 28 weeks in duration, including two 12-week treatment phases where eligible subjects were randomized to 1 of 3 treatments (bryostatin 20µg, bryostatin 40µg or placebo) for the first 12 weeks of the study. At the end of the 12 weeks, subjects in the 2 active treatment arms were to be randomized to continue on the initial dose or to receive an alternate dose (bryostatin 20µg or 40µg, depending on the initial bryostatin dose received) for an additional 12 weeks and subjects in the placebo arm were to be randomized either to continue on placebo or to receive bryostatin 10µg for an additional 12 weeks. Amended protocol version 3.0 (dated 15-Jul-2016), dropped the second 12-week treatment phase from the study design. This study is now 15 weeks in duration, including a safety and efficacy evaluation 30 days after the last dose of study drug.

Details of the proposed PK analysis will be covered by WCT Bioanalytical Sciences Group prior to the pharmacokinetic analysis in a separate PK data analysis plan (PAP). The PAP will describe the analysis population the modeling methods and possible covariates that will be examined, as well as the tables, figures, and listings.

2 STUDY OBJECTIVES

Primary objective:

- To evaluate the safety and tolerability of bryostatin 1 (hereafter referred to as bryostatin) for the treatment of moderately severe to severe AD.

Secondary objective:

- To evaluate the efficacy of bryostatin in the treatment of moderately severe to severe AD.

Exploratory objectives:

- To characterize the pharmacokinetics (PK) of bryostatin in subjects with AD following administration of 20 or 40µg of bryostatin administered as a 45 minute intravenous (IV) infusion over 12 weeks of treatment.

- To characterize the pharmacodynamics of bryostatin in subjects with AD following administration of 20 or 40µg of bryostatin administered as a 45 minute IV infusion over 12 weeks of treatment.

3 ENDPOINTS

3.1 Primary Endpoint

3.1.1 Safety

- Treatment emergent AEs (TEAEs)
- Treatment emergent SAEs

3.1.2 Efficacy

- Change from baseline in the Severe Impairment Battery (SIB) score at Week 13

3.2 Secondary Endpoints

3.2.1 Safety

- Vital signs
- Hematology
- Blood chemistry
- Physical examination (PE) including body weight
- Electrocardiogram (ECG) parameters
- Columbia-Suicide Severity Rating Scale C-SSRS

3.2.2 Efficacy

- Change from baseline in the SIB at Weeks 5 and 9
- Change from baseline in Alzheimer Disease Cooperative Study Activities of Daily Living Inventory-Severe Impairment Version (ADCS-ADL-SIV) score at Weeks 5, 9, and 13
- Change from baseline in Mini-Mental State Examination (MMSE)-2 score at Weeks 5, 9, and 13
- Change from baseline in Neuropsychiatric Inventory (NPI) score at Weeks 5, 9, and 13

- Clinical Global Impression of Improvement (CGI-I) at Weeks 5, 9, and 13

4 SAMPLE SIZE

Since the primary objective of the study is to obtain safety and tolerability data for the administration of multiple doses of bryostatin, the planned sample size is based largely on feasibility. However, the following is an assessment of power for the primary analysis of primary efficacy endpoint, estimation of the effect of bryostatin on the mean change from baseline in the SIB at Week 13.

It is estimated that 150 subjects equally randomized among the 3 dosing groups (2 doses of bryostatin and placebo) will provide at least 80% power, assuming a true mean difference of at least 6.5 points in favor of the bryostatin groups (standardized effect size of 0.47), in a test between each bryostatin group, and the polled bryostatin groups, and placebo in mean change from baseline in the SIB, (one-sided at α=0.10). This assumed the standard deviation of change from baseline at Week 13 is 14 points. This estimate allowed that up to 15% of randomized subjects would not provide information due to discontinuation. The least significant difference for the test between the observed means under these conditions was estimated to be 3.9 points (standardized effect size of 0.28).

5 RANDOMIZATION

Using a stratified randomization based on MMSE-2 score (4-9 vs. 10-15), eligible subjects were randomized 1:1:1 to 1 of 3 treatment arms: bryostatin 20µg, bryostatin 40µg or placebo.

Prior to amendment 3, after 12 weeks of treatment, eligible subjects were randomized for an additional 12 treatment period. Initial MMSE-2 scores were used for stratification. Subjects in the 20µg group were randomized 1:1 either to the same dose or to the 40µg dose. Subjects in the 40µg group were randomized 1:1 either to the same dose or to the 20µg dose. Subjects in the placebo group were randomized 1:1 either to continue on placebo or to the bryostatin 10µg dose.

6 PLANNED ANALYSES

This study contained 2 double-blind treatment phases/periods until protocol amendment 3.0. Subjects that were in the second treatment period discontinued the study once protocol amendment 3.0 was approved. The primary treatment period of interest is the initial treatment period and hereafter treatment period references this initial treatment period. The second treatment period will be referred to as treatment period 2 and unless otherwise stated, these data will only be listed. For full definition, see Section 6.2.10.2.

No statistical analysis plan (SAP) prepared in advance of the data can be absolutely definitive and the final clinical study report (CSR) may contain additional tables or statistical tests if

warranted by the data obtained. The justification for any such additional analyses will be fully documented in the final CSR.

6.1 Analysis Sets

Subjects excluded from the analysis sets and the reason for their exclusion will be listed in Appendix 16.2.

6.1.1 Enrolled Set

The Enrolled Set (ES) includes all subjects screened or those who give informed consent and are allocated a subject number. Subjects who are rescreened and assigned a new subject number will be counted only once.

6.1.2 Randomized Set

The Randomized Set (RS) consists of all subjects that are randomized.

6.1.3 Full Analysis Set

The Full Analysis Set (FAS) used for efficacy analyses, consistent with the intent-to-treat (ITT) principles, is defined as all randomized subjects who receive at least 1 dose of study medication and who have at least 1 post-baseline efficacy assessment.

6.1.4 Per-Protocol Set

A Per Protocol Analysis Set (PP) is not planned. Major protocol deviations will be summarized. A supportive data listing of protocol deviations (major/minor) will be provided.

6.1.5 Safety Analysis Set

The Safety Analysis Set (SA) is defined as all randomized subjects who receive any study medication (either partial or completed infusions of Bryostatin or Placebo). For safety analysis subjects will be grouped on an as-treated basis. If subjects received different doses over the course of the study, they will be grouped according to the dose most often administered. In addition, all treated subjects will be grouped and compared to placebo subjects.

6.1.6 Complete Analysis Set

The completer set includes all subjects in the FAS who have completed the week 13 evaluation of SIB.

6.2 Derived Data

This section describes the derivations required for statistical analysis. Unless otherwise stated, variables derived in the source data will not be re-calculated.

6.2.1 Age

Age at screening will be calculated in SAS as

age = floor ((intck('month',*birthdate*,*date*) - (day(*date*) < day(*birthdate*))) / 12);

6.2.2 Screening

For each variable where a subject is re-screened, his/her latest non-missing value from the original or re-screening assessment will be taken for tabulation purposes.

6.2.3 Baseline and Change from Baseline

Baseline is defined as the last non-missing value (either scheduled, unscheduled or repeat) before the subject receives first dose of study medication (Week 0).

Change from baseline CHG=AVAL-BASE where AVAL is the post-baseline value, BASE is the baseline value.

6.2.4 Duration/Study Day/Time

Study day will be calculated as the number of days from first dose of study medication (ie, for the analysis, the study day is relative to the first dose of study medication which will be deemed "Day 1"; note that this is a different definition than the one in Protocol NTRP-1010-202 V4, where the first dose of the study medication is given on Day 0).

- date of event – date of first dose of study drug + 1, for events on or after first dose
- date of event – date of first dose of study drug, for events before first dose.

Duration (e.g., event duration/medication usage) = stop date-start date +1

6.2.5 Conventions for Missing and Partial Dates

All rules explained below for partial/missing dates will be followed unless contradicted by any other data recorded on the electronic Case Report Form (eCRF).

All dates presented in the individual subject listings will be as recorded on the eCRF (i.e., not completed as per the rules below).

6.2.6 Missing/Partial Start/Stop Date of Adverse Events and Concomitant Medications

Partial or missing stop date will be imputed as follows:

If the stop date is completely missing and the event has resolved or the subject has stopped taking the concomitant medication, the stop date will be imputed as the date of the subject's last clinic visit in the study.

- If only the year is known, the stop date will be imputed as "31-Dec" of that year or as the date of the subject's last clinic visit in the study if in the same year.
- If the month and year are known, the stop date will be imputed as the last day of that month unless the stop date corresponds to the same month as the subject's last clinic visit in which case the date of the subject's last clinic visit in the study will be used instead.

Missing start date will be imputed as follows:

- If the stop date occurs on or after the start of study drug or the event/concomitant medication is ongoing, the start date will be imputed as the date of the first dose of study drug.
- If the stop date occurs before the start of study drug, the start date of the event/concomitant medication will be imputed as the subject's screening date or the stop date of the event/concomitant medication whichever the earlier.

Partial start date (year present, but month and day missing):

- If the stop date occurs on or after the start of study drug or the event/concomitant medication is ongoing, and the year is the same as the year of first dosing the start date will be imputed as "01-Jan" of the same year or the date of the first dose of study drug whichever is later. If the year is different from the year of first dosing "01-Jan" will be used.
- If the stop date occurs before the start of study drug, the start date of the event/concomitant medication will be imputed as the "01-Jan" of the same year.

Partial start date (month and year present, but day missing):

- If the stop date occurs on or after the start of study drug or the event/concomitant medication is ongoing, the start date will be imputed as the first day of the same month and year unless this partial start date is in same month as the first dose of study drug in which case the date of first dose of study drug will be used.
- If the stop date occurs before the start of study drug, the start date will be imputed as the first day of the month and year of the partial stop date.

Missing start/stop time of adverse events:

- If the start time of the adverse event (AE) is missing, it will imputed only in the case where the start date of the AE corresponds to the date of the first dose of study drug. The

time will be imputed as the same time as the first dose of study drug. In all other cases the time will not be imputed.

- If the stop time of the adverse event is missing, it will be imputed as 23:59 for stop time.

6.2.7 Missing Diagnosis Dates

If the month and year are present but the day is missing, the diagnosis date will be set to the first day of the relevant month. If only the year is recorded the diagnosis date will be set as "01-Jan" for that year.

6.2.8 Exposure to Study Drug

6.2.8.1 Calculation of dose taken and percentage of planned dose taken

Per protocol, study drug (bryostatin or placebo sterile lyophilized powder in 10 ml vials) should be reconstituted with 1 mL of PET diluent (resulting in 50 µg bryostatin per mL or 0.0 µg bryostatin per mL). After swirling the vial to completely dissolve the contents, the resulting solution must be diluted immediately with 9 mL of 0.9% sodium chloride (NS) injection, United States Pharmacopeia (USP). The necessary volume of this solution to achieve the assigned dose for the subject should then be added to an IV infusion bag containing 50 mL of normal saline.

Thus, the total volume of the infusion varies depending on the randomized treatment.

After diluting the 1 mL PET solution with 9 mL of 0.9% sodium chloride (NS), the resulting solution will result in 5 µg bryostatin per mL or 0.0 µg bryostatin per mL.

Subjects are supposed to receive a total of 7 doses with the first 2 doses being loading doses. The following table lists the total volume of 1 infusion for each treatment group.

Dose	1 infusion (total volume in mL)
Placebo	50 mL
20 µg	54 mL
24 µg (loading dose)	54.8 mL
40 µg	58 mL
48 µg (loading dose)	59.6 mL

The following table details total planned doses in terms of planned volume for 7 doses for each randomized treatment group. It also states the criteria to determine if a subject received at least 80% of his/her planned dose.

Dose	Planned dose in terms of planned volume	80% planned dose
Placebo	350 mL	280 mL
20 µg	379.6 mL	303.7 mL
40 µg	409.2 mL	327.4 mL

6.2.9 Inexact Values

In the case where a variable is recorded as ">x", "≥x", "<x" or "≤x", then for analysis purposes a value of x will be taken, with x fulfilling the record of the variable. Where a range of values is quoted the midpoint of the range will be taken.

6.2.10 Study Period/Analysis Windows

6.2.10.1 Pre-Treatment Period

The Pre-Treatment Period is defined as the period of time between the subject signing the informed consent through immediately prior to the subject receiving the first dose of study medication.

6.2.10.2 Treatment Period

The primary treatment period of interest is the initial treatment period and will be referred to as treatment period. The second treatment period will be referred to as treatment period 2.
The treatment period starts on the day and time of first dose of study medication and ends on Week 13 Visit. This is the primary treatment period of interest.

For those subjects that were randomized for the second treatment period and had the first dose of second treatment period study medication on Week 13 visit, the treatment period ends prior to the infusion date and time for first dose of the second treatment period on Week 13 Visit.
Hence any data collected prior to the infusion date and time on Week 13 Visit would be assigned to the initial treatment period. The data collected after that date and time will be assigned to treatment period 2 unless otherwise stated. For assessments at Week 13 where only date was collected and per protocol the assessments were pre-dose, these would be assigned to the initial treatment period.

6.2.10.2.1 Early Termination efficacy assessments

For analysis purposes, efficacy data collected from early termination (ET) visit will be assigned to the next scheduled visit. This only pertains to ET visits that occur during the treatment period.

Subjects who complete the treatment period but discontinue during the second treatment period are considered completers in the main treatment period analyses.

6.2.10.2.2 30-Day Follow-Up Visit

A follow-up visit should be scheduled 30 days after the last dose of study drug for all subjects that complete or are withdrawn from the study.

Follow-up visits will be assigned to the treatment period of the last dose of study drug taken by subject (and hence summarized under the treatment assigned for that treatment period.).

For details regarding concomitant medications and AEs please see corresponding Sections 6.8 and 6.12.1.

6.2.11 Diagnosis of AD

Diagnosis of AD is collected in medical history. Medical history is coded using MedDRA version 18.1. Years from Diagnosis of AD to Screening will be calculated based on preferred term for AD. Terms to be pulled will be finalized prior to unblinding.

6.2.12 Severe Impairment Battery (SIB)

The SIB is used to assess cognition in subjects with moderate and severe AD. There are forty questions that are divided into 9 subscales that include attention, language, orientation, memory, praxis, visuospatial ability, construction, social skills, and orienting head to name. Non-verbal responses are allowed, thus decreasing the need for language output. Most items have a point range of 0-2 except for item 19 and 24. The point score range for SIB total score (sum of the 40 items) is 0-100. Lower scores indicate greater cognitive impairment.

	Items	Point range
SIB Total Score	All	0-100
Social interaction	1 (a,b,c)	0-6
Orientation	3,5, 7	0-6
Visuospatial ability	27,29,31,33	0-8
Construction	35(a,b)	0-4
Language	4 (a,b),6,8 (a,b), 9(a,b,c),11 (a,b),13,15,17, 19(0-1 value),20,22, 24 (0-1 value),26,30 (a,b,c), 34(a,b),40	0-46
Memory	2, 10,14,25,28,32, 38	0-14
Praxis	16,18,21,23	0-8
Attention	12,36,37	0-6
Orienting to name	39	0-2

6.2.13 Alzheimer Disease Cooperative Study Activities of Daily Living Inventory-Severe Impairment Version (ADCS-ADL-SIV)

The ADCS-ADL-SIV is a 19-item functional assessment of the performance of activities of daily living for subjects with moderate to severe AD. Informants (Caregiver) are queried via a structured interview format as to whether subjects attempted each item in the inventory during the previous 4 weeks, as well as their level of performance. Each item is rated from the highest level of independent performance to complete loss. The questionnaire is split into two types of questions, an initial question relating to whether a subject has completed a particular activity and then a follow on question which scores how much assistance the subject has required if they have performed that particular activity. Note if a caregiver responds to the question relating to whether the subject performed a particular activity as no or don't know this will contribute a zero to the total score. Total score (sum of the 19 items) range from 0-54 with lower scores indicating greater functional impairment.

6.2.14 Mini Mental State Examination 2 (MMSE-2)

The standard version of the MMSE-2, with 2 alternate forms, is used in this study. The MMSE-2 is a brief, widely used test for assessing overall cognitive state. The MMSE-2 (sum of 11 items) measures selected aspects of cognition such as memory, orientation, attention, language, and praxis on a scale of 0-30. Lower scores indicate greater cognitive impairment.

6.2.15 Neuropsychiatric Inventory (NPI)

The NPI is a behavior rating scale composed of a 12-item structured interview of the caregiver that is scored from 0 to 144 (the higher the score, the greater the psychiatric disturbance). The NPI assesses 12 behavioral and psychological disturbances occurring in dementia patients: delusions, hallucinations, agitation/aggression, depression/dysphoria, anxiety, elation/euphoria, apathy/indifference, disinhibition, irritability, aberrant motor behavior, sleep and nighttime behavior disorders, and appetite and eating disorders. Both the frequency and the severity of each behavior are determined.

Scoring the NPI:

Category	Numeric values assigned
Frequency is rated as:	1 = Occasionally - less than once per week<br>2 = Often - about once per week<br>3 = Frequently - several times per week but less than every day<br>4 = Very frequently - daily or essentially continuously present
Severity is rated as:	1 = Mild - produces little distress in the patient<br>2 = Moderate - more disturbing to the patient but can be redirected by the caregiver<br>3 = Severe - very disturbing to the patient and difficult to redirect
Caregiver Distress is rated as:	0 = no distress<br>1 = minimal<br>2 = mild<br>3 = moderate<br>4 = moderately severe<br>5 = very severe or extreme.
Category	Numeric values assigned

The total score for each of the 12 domains is domain score = frequency x severity

Thus, for each behavioral domain there are 4 scores:

- Frequency
- Severity

- Total (frequency x severity)
- Caregiver distress

A total NPI score will be calculated by adding the total scores of the 12 domain scores together. The caregiver distress score is not included in the total NPI score. The total distress score is generated by adding together the scores of the 12 items of the NPI distress questions.

6.2.16 Clinical Global Impression of Improvement (CGI-I)

The CGI-I is used to assess global change in the subject's condition compared to baseline before treatment. This is a 7-point scale ranging from (1) very much improved to (7) very much worse. A score of 0 means that the scale was not assessed and hence this value is not included in any summary/analysis unless stated otherwise.

6.2.17 Rosen-Modified Hachinski Scale

The Rosen-Modified Hachinski Scale will be evaluated at screening to differentiate Alzheimer's type dementia from multi-infarct dementia. The 8-item scale results in a score of 0-12.

6.2.18 Electrocardiogram Data

For ECG data recorded on continuous scales, if more than 1 value is recorded at a time point, the mean value rounded to the integer will be presented. For overall interpretation if more than 1 value is recorded, the most severe (worst case) of the respective readings will be taken. -

6.2.19 Columbia-Suicide Severity Rating Scale

The following outcomes are C-SSRS categories and have binary responses (yes/no). The categories have been re-ordered from the actual scale to facilitate the definition of composite endpoints:

Category 1	Wish to be Dead
Category 2	Non-specific Active Suicidal Thoughts
Category 3	Active Suicidal Ideation with Any Methods (Not Plan) without Intent to Act
Category 4	Active Suicidal Ideation with Some Intent to Act, without Specific Plan
Category 5	Active Suicidal Ideation with Specific Plan and Intent
Category 6	Preparatory Acts or Behavior
Category 7	Aborted Attempt
Category 8	Interrupted Attempt
Category 9	Actual Attempt (non-fatal)
Category 10	Completed Suicide

Suicidal Ideation since baseline – A “yes” answer at any time during double-blind treatment to any one of the 5 suicidal ideation questions (categories 1-5) on the C-SSRS.

Suicidal Behavior since baseline – A “yes” answer at any time during double-blind treatment to any one of the 5 suicidal behavior questions (categories 6-10) on the C-SSRS.

There will be no imputation of missing data for C-SSRS.

6.2.20 Unscheduled Visits

In general, only scheduled post-baseline laboratory and vital signs values will be tabulated. However, post-baseline repeat/unscheduled assessments could be included if an “any time, post-baseline” summary is specified. At minimum, these post-baseline assessments will be listed in the relevant appendices to the CSR.

6.3 Conventions

All data listings, summaries, figures and statistical analyses will be generated using SAS version 9.3 or higher[1].

Summaries will be presented by treatment group or overall. Treatment group labels will be displayed as follow:

Placebo	Bryostatin (20 µg)	Bryostatin (40 µg)	Bryostatin (20+40 µg)

Overall columns are to be included within the table shells as follows:

Demography	*Treatment and overall*
Baseline	*Treatment and overall*
Disposition	*Treatment and overall*
Efficacy	*Treatment*
PD	*Treatment*
AEs	*Treatment & overall*
Other safety	*Treatment*

Listings will be sorted in the following order: treatment group, subject, parameter, and visit unless otherwise stated.

Continuous variables will be summarized by the number of non-missing observations, mean, median, standard deviation, and minimum and maximum.

Categorical variables will be summarized by presenting the frequency and percent. Percentages will be based on the number of non-missing observations or the subject population unless otherwise specified. For each variable, all categories will be shown. Zero frequencies (but not the percent) within a category will be presented.

6.3.1 Decimal Places

Decimal places for derived data will be determined by the scale of measurement unless otherwise stated. No decimal places will be displayed if the smallest calculated value is ≥ 100; 1 decimal place will be displayed when the smallest value is within the interval (10, 100), with 10 being inclusive; 2 decimal places will be displayed when the smallest value is within (1, 10), with 1 being inclusive; and so on for even smaller scales of measurement.

For derived data where known in advance that the result will be an integer (e.g., day, month, year, number of days and total scores [for rating scales]) will be presented with zero decimal places unless otherwise stated.

Means, medians, and percentiles will be displayed to one more decimal place than the data**,** dispersion statistics (e.g., standard deviation) will have 2 more decimal places, and the minimum and maximum will be displayed to the same number of decimal places as reported in the raw data. Percentages will be displayed with 1 decimal place.

P-values will be quoted to 3 decimal places. P-values < 0.001 will be presented as $p<0.001$. Where $p < 0.05$, $p < 0.01$, or $p < 0.001$, attention will be drawn to this fact using the conventional "*", "**" or "***" annotation, respectively.

6.4 Subject Disposition

Subject disposition will be summarized as follows:

- The number of subjects, who were enrolled in the study, were randomized, and are in each analysis set will be summarized by treatment group and overall for the ES.
- The number of subjects from the main treatment period that were randomized into treatment period 2 and received study medication for the SA set.
- The number of subjects who were rescreened, failed screening and the reasons for screen failure will be tabulated for ES
- The number of treatment completers, early withdrawals and the reasons for withdrawals will be tabulated by treatment group and overall for SA and FAS set.
- The number of subjects present at each scheduled visit and number of subjects whose SIB were not done within 21 days before the first study drug dose will be summarized by treatment group for the SA set.
- Number of SA set subject by site will be summarized.

6.5 Protocol Deviations

A summary of major protocol deviations will be summarized.

Furthermore a listing of protocol deviations will be provided in Appendix 16.2.

6.6 Baseline Comparability

The comparability of treatment group with respect to subject demographics and baseline characteristics will be assessed in a descriptive manner, but no formal statistical testing will be performed.

Standard continuous or categorical variable summaries will be presented by treatment group for the SA and FAS set.

Demographic and Baseline Characteristics

The following variables will be summarized:

- Age at screening visit (years)
- Gender (Male, Female)
- Ethnicity (Hispanic or Latino, Not Hispanic or Latino)
- Race (American Indian or Alaska Native, Asian, Black or African American, Native Hawaiian or Other Pacific Islander, White)
- MMSE-2 (continuous, stratum [4-9 vs 10-15])
- Height, weight and BMI at screening
- Rosen-Modified Hachinski Score
- Years from diagnosis of AD to Screening (derived based on medical history)
- Concurrent use of AD drugs by type (cholinesterase inhibitor, memantine, both)

6.7 Medical History

Separate tabulations of previous and ongoing conditions at screening will be presented by treatment group and overall for the SA Set. Conditions will be presented *by Medical Dictionary of Regulatory Activities* (MedDRA version 18.1) primary system organ class and preferred term.

6.8 Prior and Concomitant Medications

Separate tabulations will be produced for prior and concomitant medications presented by treatment group and overall for the SA and FAS Set. Medications will be summarized using Anatomical Therapeutic Chemical (ATC) level 2 and drug reference name using the World Health Organization (WHO) dictionary (Version WHODDE MAR2015).

Medications will be categorized by start and stop dates of medication in relation to start date of study drug and could be categorized in more than one category (e.g., a medication taken prior to first dose of study drug but on-going through the treatment period (s), will be considered both a prior and concomitant medication). Prior medications are defined as all medications starting before the date of first dose of study medication. Concomitant medications are defined as

medications taken on or after the date of first dose of study medication up to the date of last study treatment in the main treatment period. For subjects who received study medication during treatment period 2, medications started on the same day as first dose of the second treatment period, will be assigned to that second treatment period only.

Frequency distribution of drugs used to treat AD by treatment arm will be presented.

6.9 Exposure to Study Drug/Treatment compliance

Number and percentage of subjects who did or did not have an infusion, had a full infusion, had partial infusion by treatment group and visit will be presented. Descriptive statistics of the number of infusions will be presented by treatment group.

Number and percentage of subjects who had 80% of the (cumulative) planned dose will be summarized by treatment group (see Section 6.2.8.1).

6.10 Efficacy Analyses

Efficacy analyses will be based on FAS by randomized treatment regardless of the treatment actually received. Descriptive statistics by treatment group and visit will be reported for all efficacy endpoints. Analyses will be performed on each dosing group vs. placebo and on the "pooled" dosing group vs. placebo.

6.10.1 Primary Endpoint

The primary efficacy objective is to determine if treatment with bryostatin (20 or 40 µg) improves cognitive function as measured by improvement on SIB after 12 weeks of treatment compared to placebo.

The primary efficacy endpoint is change from baseline in the Severe Impairment Battery (SIB) score at Week 13. Each bryostatin group will be compared to the placebo group and the pooled bryostatin group to placebo group.

The hypothesis test is as follows:

H_o: $\mu_{bryostatin} \leq \mu_{placebo}$

H_1: $\mu_{bryostatin} > \mu_{placebo}$

The null hypothesis will be rejected at a significance level of α (1-sided) = 0.10. There will be no p-value adjustment for multiplicity.

6.10.2 Primary Efficacy Analysis

The primary endpoint will be analyzed using Mixed Model for Repeated Measures (MMRM) with fixed effects for treatment, baseline MMSE-2 stratum, scheduled visit (nominal) and scheduled visit by treatment interaction, random effect for subject and baseline SIB as a covariate. The model will include two treatment groups (i.e. placebo and one bryostatin dose group). All statistical tests will be 1-sided with $\alpha = 0.10$. Least-square means (LSM) and 2-sided 80% confidence intervals (CI) will be provided for treatment group differences and estimated endpoint values by visit.

Sample SAS code for two-sided 80% confidence interval.
Treat: 1=Placebo, 2=study drug
Visit 13: Week 13

```
PROC MIXED DATA = <DATASET>;
  CLASS TREAT VISIT BASE_MMSE_STRATUM SUBJID;
  MODEL CHANGE = TREAT VISIT TREAT*VISIT BASE_MMSE_STRATUM
BASELINE_SIB/
                        DFM=KENWARDROGER ;
  REPEATED VISIT / TYPE=UN  SUB=SUBJID  GROUP=TREAT;
  LSMEANS TREAT*VISIT/PDIFF=CONTROL('1'  '13') ALPHA=0.2;
RUN;
```

Sample SAS code for one-sided p-value and 80% confidence interval.

```
PROC MIXED DATA = <DATASET>;
  CLASS TREAT VISIT BASE_MMSE_STRATUM SUBJID;
  MODEL CHANGE = TREAT VISIT TREAT*VISIT BASE_MMSE_STRATUM
BASELINE_SIB/
                          DDFM=KENWARDROGER ;
  REPEATED VISIT / TYPE=UN  SUB=SUBJID  GROUP=TREAT;
  LSMEANS TREAT*VISIT/PDIFF=CONTROLU('1'  '13') ALPHA=0.2;
RUN;
```

Note: the SAS code could be modified due to data limitation and convergence issue.

6.10.3 Sensitivity Analysis

No sensitivity analysis is planned for the study.

6.10.4 Exploratory Analysis

Exploratory analyses will be performed to evaluate the impact of baseline covariates on the primary efficacy variables. Such analyses may be described in the clinical study report.

- A re-analysis of the primary endpoint using the MMRM model and excluding all patients from sites that recruited 2 or fewer patients.
- Last observation carried forward (LOCF) analysis: Missing SIB assessment at Week 13 will be imputed using the LOCF method for subjects with post-baseline assessments. An analysis of covariance (ANCOVA) for the Week 13 Visit will be conducted. The model will include treatment, and baseline SIB as a covariate.
- Site will be added as an additional covariate in above ANCOVA to assess site effect.
- Use of AChEI, Memantine at baseline will be added as an additional covariate in above ANCOVA to assess site effect.
- Subjects will be divided into two subgroups: 1) baseline SIB<=median baseline SIB, 2) baseline SIB>median baseline SIB. An analysis of covariance (ANCOVA) for the Week 13 Visit will be conducted to assess treatment difference (low dose vs. placebo, high dose vs. placebo). The model will include treatment, and baseline SIB as a covariate. Missing SIB assessment at Week 13 will be imputed using the LOCF method for subjects with post-baseline assessments.
- The Following responder analyses at week 13 will be conducted to compare the proportions of responders between treatment groups (low dose vs. placebo, high dose vs. placebo, pooled dose vs. placebo) using CMH approach. Missing assessment at Week 13 will be imputed using the LOCF method for subjects with post-baseline assessments.
  A. A responder is defined as a subject who had improvement on the SIB.
  B. A responder is defined as a subject who had improvement of at least 3.9 points on the SIB.
  C. A responder is defined as a subject who had improvement of at least 6.5 points on the SIB.
  D. A responder is defined as a subject who had improvement either on SIB or on ADCS-ADL-SIV.

The same analyses will be performed for pooled dose vs. placebo.

6.10.5 Secondary Endpoints

Secondary endpoints for ADCS-ADL-SIV, MMSE-2 (excludes MMSE Stratum variable), NPI at week 13 will be analyzed using a statistical model similar to the one used for analysis of the SIB. The CGI-I secondary endpoint will be analyzed in similar fashion except the model will not have a baseline value used as a covariate.

The secondary efficacy endpoints will not be formally tested against the null hypothesis; however, p-values and CI will be used to support the results of the primary endpoint analysis.

6.10.6 Exploratory Endpoints

The exploratory PK endpoints will be addressed in a separate analysis plan.

The following exploratory endpoints will be analyzed using the same statistical model as the primary endpoint:

- Change from baseline in SIB and MMSE Scores at the 30-day follow up
- Change from Week 13 in SIB and MMSE Scores at the 30-day follow up.

Subjects who are randomized at treatment period 2 will be excluded at the 30-day follow up.

ANCOVA model described in section 6.10.4 will be used for SIB, ADCS-ADL, NPI, and MMSE-2 by visit.

ANCOVA model will be used for ADCS-ADL, NPI, and MMSE-2 at Week 13. Missing assessment at Week 13 will be imputed using the LOCF method for subjects with post-baseline assessments.

Subjects will be divided into two subgroups: 1) baseline ADCS-ADL <= median baseline ADCS-ADL, 2) baseline ADCS-ADL > median baseline ADCS-ADL. An analysis of covariance (ANCOVA) for the Week 13 Visit will be conducted to assess treatment difference (low dose vs. placebo, high dose vs. placebo). The model will include treatment, and baseline ADCS-ADL as a covariate. Missing ADCS-ADL assessment at Week 13 will be imputed using the LOCF method for subjects with post-baseline assessments. The same analyses for NPI, and MMSE-2 at Week 13 will also be conducted.

The effects of center and treatment by center interaction will be explored using an analysis of covariance (ANCOVA) model as describe in section 6.10.4 if data permit. Low enrolling centers may be pooled if appropriate. Stepwise covariates selection approach may be applied. These will explored as post hoc analyses.

6.10.7 Multiplicity

All secondary endpoints and the supportive analyses will be considered as descriptive evidence of efficacy and will be analyzed without any procedures to account for multiple comparisons.

6.11 Pharmacodynamic Analyses

PKCε concentration prior to the infusion, at the end of infusion (+5minute window) and 60 minutes after completion of infusion (±5minutes) will be collected from selected sites for doses

1, 3, and 7, respectively. Descriptive summary will covered by WCT Bioanalytical Sciences Group.

6.12 Safety Analyses

The safety analyses will be presented by the actual treatment received for the SA Set.

6.12.1 Adverse Events

A TEAE in the main treatment period is defined as follows:

- Any AE with an onset on or after the first dose of study drug and through 30 days after the last dose of study drug in the main treatment period.
- Any pre-existing AE that has worsened in severity on or after the first dose of study drug and through 30 days after the last dose of study drug in the main treatment period.

A TEAE in treatment period 2 is defined as follows:

- Any AE that has an onset on or after the first dose of treatment period 2 at the Week 13 Visit

A treatment-related AE is defined as an AE as being *possibly or probably* related to the study drug. If an AE has a missing relationship, it is assumed to be related to the study drug for analysis purposes.

Maximum severity will be assumed for an AE with missing severity.

The following tables will be presented for AEs in the main treatment period:

- Overall incidence and the number of AEs, SAEs, treatment emergent adverse events of special interest (AESIs)-myalgia, TEAEs leading to withdrawal of study drug.
- TEAE by system organ class and preferred term, incidence and number of events
- Treatment related TEAE by system organ class and preferred term, incidence and number of events
- Serious TEAE by system organ class and preferred term, incidence and number of events
- TEAESI-myalgia by system organ class and preferred term, incidence and number of events
- TEAEs leading to withdrawal of study drug by system organ class and preferred term, incidence and number of events
- TEAE by system organ class, preferred term and maximum severity, incidence
- Listing of serious TEAEs (presented in the Table section of the appendices)

- Listing of deaths (presented in the Table section of the appendices)
- Listing of relationship of AE verbatim term with MedDRA

Adverse events will be coded using MedDRA version 18.1.

A complete subject listing of all AEs will be provided. This listing will include treatment, AE verbatim term, primary system organ class and preferred term, the time of onset and cessation of event relative to the first dose of study medication, duration of AE (for ongoing AEs, no duration will be calculated), whether serious, severity, relationship to study medication, action taken and outcome.

6.12.2 Laboratory Data

Descriptive statistics of the observed values and change from baseline (continuous data) will be presented by treatment group and visit for each hematology, coagulation, CBC with differential, and serum chemistry parameter. Each measurement (continuous data) will be classed as below, within, or above normal range, based on ranges supplied by the laboratory used. Shift tables in relation to the normal range from baseline to each follow-up visit will be presented.

Liver Function Parameter Abnormalities

The number and percentage of subjects meeting certain liver function abnormality categories will be summarized by visit and any post-baseline visit (e.g., post-dose) during the treatment period:

- Alanine Aminotransferase (AST) assessment > 3 times upper limit of normal (ULN),
- Aspartate aminotransferase (AST) > 3xULN,
- ALT or AST: > 3xULN; > 10xULN; > 20xULN
- Total Bilirubin: > 1.5xULN; > 2xULN
- Alkaline Phosphatase: > 1.5xULN; >2xULN
- (ALT or AST > 3xULN) and total bilirubin>2xULN
- (ALT or AST>3xULN) and total bilirubin>2xULN and alkaline phosphatase<2xULN

Box plots for the above lab parameters will be provided for changes from baseline and maximal change. In addition, Scatter Plots of LFT Parameters Post-Baseline vs Baseline.

Lab Parameter Shift Tables

Shift tables in relation to the normal range from screening to each follow-up visit will be presented for Hematology and Chemistry. In addition, Shifts from baseline will be evaluated for

following Lab parameters based on Common Terminology Criteria for Adverse Events (CTCAE 4.03, 2010-06-14) grade. The baseline and highest post-dose results for each of these parameters will be categorized by CTCAE grade. The number and percentage of subjects in each baseline by post-baseline category will be summarized by analysis category. Separate shift tables will be prepared for the highest post-dose assessment and one for the last post-dose assessment during the treatment period. This will include all scheduled and unscheduled laboratory assessments.

A table displaying the CTCAE grades for lab parameters of interest is provided below:

Parameter (unit)	Grade 1	Grade 2	Grade 3	Grade 4
APTT (seconds)	>ULN-1.5xULN	>1.5xULN-2.5xULN	> 2.5xULN	----
Alkaline phosphate (U/L)	>ULN-2.5xULN	>2.5xULN-5xULN	>5xULN-20xULN	>20xULN
ALT (U/L)	>ULN-3xULN	>3xULN-5xULN	>5xULN-20xULN	>20xULN
AST (U/L)	>ULN-3xULN	>3xULN-5xULN	>5xULN-20xULN	>20xULN
Total bilirubin (µmol/L)	>ULN-1.5xULN	>1.5xULN-3xULN	>3xULN-10xULN	>10xULN
Creatinine	> 1-1.5x baseline or > ULN-1.5xULN	>1.5-3.0 x baseline or > 1.5xULN-3.0xULN	> 3.0- 6.0 x baseline or >3xULN-6xULN	>6x baseline or > 6xULN
Creatinine clearance	60<=RESULT<LLN ml/min	59-30 ml/min	29-15 ml/min	<15 ml/min
WBC decreased	<LLN - 3000/mm3; <LLN - 3.0 x 10e9 /L	<3000 - 2000/mm3; <3.0 - 2.0 x 10e9 /L	<2000 - 1000/mm3; <2.0 - 1.0 x 10e9 /L	<1000/mm3; <1.0 x 10e9 /L
PLT (PLAT/Platelet count)	<LLN - 75,000/mm3; <LLN - 75.0 x 10e9 /L	<75,000 - 50,000/mm3; <75.0 - 50.0 x 10e9 /L	<50,000 - 25,000/mm3; <50.0 - 25.0 x 10e9 /L	<25,000/mm3; <25.0 x 10e9 /L
Hemoglobin (anemia)	Hemoglobin (Hgb) <LLN - 10.0 g/dL; <LLN - 6.2 mmol/L; <LLN - 100 g/L	Hemoglobin (Hgb) <LLN 10.0 - 8.0 g/dL; <LLN 6.2 - 4.9 mmol/L; <LLN 100 – 80 g/L	Hgb <8.0 g/dL; <4.9 mmol/L; <80 g/L	-
INR	>1 - 1.5 x ULN; >1 - 1.5 times above baseline if on anticoagulation	>1.5 - 2.5 x ULN; >1.5 - 2.5 times above baseline if on anticoagulation	>2.5 x ULN; >2.5 times above baseline if on anticoagulation	-
CPK/CK (increase)	>ULN - 2.5 x ULN	>2.5 x ULN - 5 x ULN	>5 x ULN - 10 x ULN	>10 x ULN

6.12.3 Vital Signs

Descriptive statistics for observed values and changes from baseline in the following vital signs will be presented by treatment group and visit:

- Systolic blood pressure (mmHg)
- Diastolic blood pressure (mmHg)
- Pulse rate (bpm)
- Respiration rate (breaths/min)
- Body temperature (degrees Celsius)

Vital sign values will be categorized into clinical concern (CS) categories if applicable.

Vital Sign	PCS categories
Systolic blood pressure	120-139; 140-159; ≥160 mmHg
Diastolic blood pressure	80-89; 90-99; ≥ 100 mmHg
Pulse rate	<60 or >100 bpm
Respiratory Rate	> 27 breaths/min

Number and percent of subjects falling into these categories will be summarized by Visit and Any time, post-baseline during the treatment period. For scheduled visits (excluding Week 13 Visit) where multiple assessments are collected (pre and post infusion), worst case will be counted for that visit.

Box plots for the above vital signs will be provided for changes from baseline by visit and maximal change.

Shifts from baseline to worst for hyper and hypotension for CTCAE will be evaluated for based on Common Terminology Criteria for Adverse Events (CTCAE 4.03, 2010-06-14) grade. The baseline and highest post-dose results for each of these blood pressure will be categorized by CTCAE grade. Separate shift tables will be prepared for the highest post-dose assessment and one for the last post-dose assessment during the treatment period. This will include all scheduled and unscheduled vital assessments.

A table displaying the CTCAE grades for hyper and hypotension is provided below:

Parameter (unit)	Grade 1	Grade 2	Grade 3	Grade 4
Systolic BP (mmHg)	120 - 139 mmHg	140 - 159 mmHg	>=160 mm Hg	-
Diastolic BP (mmHg)	80 - 89 mmHg	90 - 99 mmHg	>=100 mm Hg	-

6.12.4 Electrocardiogram Data

Descriptive statistics for observed values and changes from baseline in the following ECG variables will be tabulated at each post-baseline visit:

- Heart rate (bpm)
- PR interval (ms)
- RR interval (ms)
- QT interval (ms)
- QRS Duration, (ms)
- QTcF interval (ms) [Fridericia's formula - QTcF]

ECG Result Interpretation (Normal, Abnormal Not Clinically Significant [NCS], and Abnormal Clinically Significant [CS]) will be summarized by treatment group and visit.

The incidence of ECG abnormalities for subjects with any abnormal CS ECG result will be presented by treatment group and visit.

QTcF values will be categorized into the following categories:

- < 450 ms
- 450-480 ms
- 481-500 ms
- > 500 ms

Number and percent of subjects that fall into these categories will be presented by treatment group and visit. Box plots for ECG data will be provided for changes from baseline by visit and maximal change.

6.12.5 Physical Examination

The body systems within the physical examination (PE) data will be summarized by treatment group and visit (Normal; Abnormal NCS, Abnormal CS). Changes from baseline will also be tabulated. Details of CS findings will be listed.

Descriptive statistics for observed values and changes from baseline in weight will be presented by treatment group and visit. Number and percentage of subjects with > 10% change from baseline (increase or decrease) will also be summarized.

6.12.6 Second Treatment Period Analyses

AEs (i.e. an AE has an onset on or after the first dose of the second treatment period or a pre-existing AE that has worsened in severity on or after the first dose of the second treatment period.) will be summarized by treatment.

The following tables will be presented for AEs in the second treatment period:

- Overall incidence and the number of AEs, SAEs, treatment emergent adverse events of special interest (AESIs)-myalgia, TEAEs leading to withdrawal of study drug, Period 2
- AE by system organ class and preferred term, incidence and number of events, Period 2

7 INTERIM ANALYSIS

No formal interim analyses are planned.

8 DATA SAFETY MONITORING BOARD ANALYSIS

Data safety monitoring board (DSMB) analyses/summaries are described separate from this document.

9 CHANGES TO PLANNED PROTOCOL ANALYSIS

10 REFERENCES

1. SAS Institute Inc. The SAS System, Version 9.3. Cary, NC, SAS Institute Inc. 2012.

11 LIST OF TABLES, FIGURES AND LISTINGS

The following table includes details of the tables, figures and listings to be included within each section of the eCTD. The eCTD section is shown in bold. The following validation methods maybe used

- Independent programming of numbers and manual review of format (IP)
- Independent programming by statistician of numbers and manual review of format (Stat IP)
- Manual review (MR)
- Code review (CR)

Table Number	Table Title	Validation Method	Shell Number (If Repeat)
**14.1**	**Demographics Data**		
**14.1.1**	**Disposition**		
14.1.1.1	Subject Disposition, Analysis Sets<br>Enrolled Set		
14.1.1.2	Subject Disposition, Second 12-Week Treatment Phase		
14.1.1.3	Subject Disposition, Screen Failures<br>Enrolled Set		
14.1.1.4	Inclusion/Exclusion Criteria not met<br>Enrolled Set		
14.1.1.5	Subject Disposition, Early Withdrawals<br>Safety Analysis Set		
14.1.1.6	Subject Disposition, Early Withdrawals<br>Full Analysis Set		
14.1.1.7	Subject Disposition, Safety Analysis Set by Site<br>Safety Analysis Set		
14.1.1.8	Subject Disposition, Safety Analysis Set by Site<br>Full Analysis Set		
14.1.1.9	Subject Disposition, Visits<br>Safety Analysis Set		
14.1.1.10	Subject Disposition, Visits		

	Full Analysis Set		
**14.1.2**	**Demographics**		
14.1.2.1	Demographics<br>Safety Analysis Set		
14.1.2.2	Demographics<br>Full Analysis Set		
14.1.2.3	Demographics<br>Completer Analysis Set		
14.1.2.4	Baseline Characteristics<br>Safety Analysis Set		
14.1.2.5	Baseline Characteristics<br>Full Analysis Set		
14.1.2.6	Baseline Characteristics<br>Completer Analysis Set		
**14.1.3**	**Baseline Characteristics**		
14.1.3.1	Prior Medical History<br>Safety Analysis Set		
14.1.3.2	Prior Medical History<br>Full Analysis Set		
14.1.3.3	Ongoing Medical Conditions at Screening<br>Safety Analysis Set		
14.1.3.4	Ongoing Medical Conditions at Screening<br>Full Analysis Set		
14.1.3.5	Prior Medications<br>Safety Analysis Set		
14.1.3.6	Prior Medications<br>Full Analysis Set		
14.1.3.7	Major Deviations<br>Full Analysis Set		
**14.2**	**Efficacy Data**		
**14.2.1**	**Primary Efficacy Endpoint**		
14.2.1.1	Severe Impairment Battery (SIB) Scale<br>Full Analysis Set		
14.2.1.2	Severe Impairment Battery (SIB) Scale<br>Completer Analysis Set		

14.2.1.3	Mixed Models for Repeated Measures Analysis of Change from Baseline in SIB Total Score<br>Full Analysis Set		
14.2.1.4	Mixed Models for Repeated Measures Analysis of Change from Baseline in SIB Total Score<br>Completer Analysis Set		
14.2.2.1	Alzheimer Disease Cooperative Study Activities of Daily Living Inventory Severe Impairment Version (ADCS-ADL-SIV)<br>Full Analysis Set		
14.2.2.2	Mixed Models for Repeated Measures Analysis of Change from Baseline in ADCS-ADL-SIV Total Score<br>Full Analysis Set		
14.2.2.3	Mini Mental State Examination Version 2 (MMSE-2)<br>Full Analysis Set		
14.2.2.4	Mixed Models for Repeated Measures Analysis of Change from Baseline in MMSE-2 Total Score<br>Full Analysis Set		
14.2.2.5	Neuropsychiatric Inventory (NPI) Scale<br>Full Analysis Set		
14.2.2.6	Mixed Models for Repeated Measures Analysis of Change from Baseline in NPI total Score<br>Full Analysis Set		
14.2.2.7	Clinical Global Impression of Improvement (CGI-I)<br>Full Analysis Set		
14.2.2.8	Mixed Models for Repeated Measures Analysis of CGI-I Score<br>Full Analysis Set		
**14.2.3**	**Exploratory Endpoints**		

14.2.3.1.1	Analysis of Covariance of Change from Baseline in SIB Total Score<br>Full Analysis Set		
14.2.3.1.2	Analysis of Covariance of Change from Baseline in SIB Total Score to Assess Site Effect<br>Full Analysis Set		
14.2.3.1.3	Analysis of Covariance of Change from Baseline in SIB Total Score of Use of AChEI or Memantine at Baseline as an Additional Covariate<br>Full Analysis Set		
14.2.3.1.4a	Analysis of Covariance of Change from Baseline in SIB Total Score, <= Median Baseline SIB Total Score<br>Full Analysis Set		
14.2.3.1.4b	Analysis of Covariance of Change from Baseline in SIB Total Score, > Median Baseline SIB Total Score<br>Full Analysis Set		
14.2.3.1.5a	A Responder Analysis (CMH) of Subject Who Had Improvement on SIB Total Score at Week 13<br>Full Analysis Set		
14.2.3.1.5b	A Responder Analysis (CMH) of Subject Who Had Improvement of at Least 3.9 Points on SIB Total Score at Week 13<br>Full Analysis Set		
14.2.3.1.5c	A Responder Analysis (CMH) of Subject Who Had Improvement of at Least 6.5 Points on SIB Total Score at Week 13<br>Full Analysis Set		
14.2.3.1.6	Mixed Models for Repeated Measures Analysis of Change from Baseline in SIB Total Score Excluding All Patients from Sites that Recruited 2 or Fewer Patients<br>Full Analysis Set		

14.2.3.1.7	Mixed Models for Repeated Measures Analysis of Changes from Baseline and Week 13 in SIB Total Score at 30-day Follow up<br>Full Analysis Set		
14.2.3.2.1	A Responder Analysis (CMH) of Subjects Who Had Improvement either on SIB or on ADCS-ADL-SIV<br>Full Analysis Set		
14.2.3.2.2	Analysis of Covariance of Change from Baseline in ADCS-ADL-SIV Total Score<br>Full Analysis Set		
14.2.3.2.3	Analysis of Covariance of Change from Baseline in ADCS-ADL-SIV Total Score to Assess Site Effect<br>Full Analysis Set		
14.2.3.2.4a	Analysis of Covariance of Change from Baseline in ADCS-ADL-SIV Total Score, <= Median Baseline ADCS-ADL-SIV Total Score<br>Full Analysis Set		
14.2.3.2.4b	Analysis of Covariance of Change from Baseline in ADCS-ADL-SIV Total Score, > Median Baseline ADCS-ADL-SIV Total Score<br>Full Analysis Set		
14.2.3.2.5	Analysis of Covariance of Change from Baseline in MMSE-2 Total Score<br>Full Analysis Set		
14.2.3.2.6	Analysis of Covariance of Change from Baseline in MMSE-2 Total Score to Assess Site Effect<br>Full Analysis Set		
14.2.3.2.7a	Analysis of Covariance of Change from Baseline in MMSE-2 Total Score, <= Median Baseline MMSE-2 Total Score<br>Full Analysis Set		

14.2.3.2.7b	Analysis of Covariance of Change from Baseline in MMSE-2 Total Score, > Median Baseline MMSE-2 Total Score<br>Full Analysis Set		
14.2.3.2.8	Analysis of Covariance of Change from Baseline in NPI Total Score<br>Full Analysis Set		
14.2.3.2.9	Analysis of Covariance of Change from Baseline in NPI Total Score to Assess Site Effect<br>Full Analysis Set		
14.2.3.2.10a	Analysis of Covariance of Change from Baseline in NPI Total Score, > Median Baseline NPI Total Score<br>Full Analysis Set		
14.2.3.2.10b	Analysis of Covariance of Change from Baseline in NPI Total Score, <= Median Baseline NPI Total Score<br>Full Analysis Set		
**14.3**	**Safety Data**		
**14.3.1**	**Displays of Adverse Events**		
14.3.1.1	Adverse Events, Overview of Treatment-Emergent Adverse Events (TEAE)<br>Safety Analysis Set		
14.3.1.2	Adverse Events, TEAEs by Primary System Organ Class and Preferred Term<br>Safety Analysis Set		
14.3.1.3	Adverse Events, Treatment-Related TEAEs by Primary System Organ Class and Preferred Term<br>Safety Analysis Set		
14.3.1.4	Adverse Events, TEAEs leading to Study Drug Discontinuation by Primary System Organ Class and Preferred Term<br>Safety Analysis Set		
14.3.1.5	Adverse Events, Serious TEAEs by Primary System Organ Class and Preferred Term		

	Safety Analysis Set		
14.3.1.6	Adverse Events, Treatment-Related Serious TEAEs by Primary System Organ Class and Preferred Term<br>Safety Analysis Set		
14.3.1.7	Adverse Events, TEAEs of Special Interest - Myalgia<br>Safety Analysis Set		
14.3.1.8	Adverse Events, TEAEs by Primary System Organ Class, Preferred Term and Maximum Severity<br>Safety Analysis Set		
14.3.1.9	Adverse Events, Overview of Treatment-Emergent Adverse Events (TEAE), Period 2<br>Safety Analysis Set		
14.3.1.10	Adverse Events, TEAEs by Primary System Organ Class and Preferred Term, Period 2<br>Safety Analysis Set		
**14.3.2**	**Listings Of Deaths, Other Serious And Significant Adverse Events**		
14.3.2.1	Deaths, Listing<br>Enrolled Set		
14.3.2.2	SAE, Listing<br>Safety Analysis Set		
14.3.2.3	Treatment-Related TEAEs, Listing<br>Safety Analysis Set		
14.3.2.4	TEAEs leading to Study Drug Discontinuation, Listing<br>Safety Analysis Set		
14.3.2.5	TEAEs of Special Interest – Myalgia, Listing<br>Safety Analysis Set		
**14.3.3**	**Narratives of Deaths, Other Serious and Certain Other Significant Adverse Events**<br>*(This is usually not created by stats but this placeholder)*		
**14.3.4**	**Abnormal Laboratory Values**		

14.3.4.1	Hematology<br>Safety Analysis Set		
14.3.4.2	Shift Table-Hematology<br>Safety Analysis Set		
14.3.4.3	Chemistry<br>Safety Analysis Set		
14.3.4.4	Shift Table-Chemistry<br>Safety Analysis Set		
14.3.4.5	Liver Function Abnormalities<br>Safety Analysis Set		
14.3.4.6	Shift for Selected Lab Parameters from Baseline to Last Assessment Based on CTCAE Grade<br>Safety Analysis Set		
14.3.4.7	Shift for Selected Lab Parameters from Baseline to Highest Post-Baseline Value based on CTCAE Grade<br>Safety Analysis Set		
**14.3.5**	**Extent Of Exposure, Dosage Information, And Compliance**		
14.3.5.1	Infusions<br>Safety Analysis Set		
**14.3.6**	**Vital Signs And Physical Examination**		
14.3.6.1	Vital Signs<br>Safety Analysis Set		
14.3.6.2.1	Vital Sign Data outside Clinical Concern Range<br>Safety Analysis Set		
14.3.6.2.2	Shift for Systolic BP and Diastolic BP from Baseline to Worst CTCAE Grade<br>Safety Analysis Set		
14.3.6.3	Physical Examinations<br>Safety Analysis Set		
14.3.6.4	Physical Examinations, Changes from Baseline<br>Safety Analysis Set		
14.3.6.5	Physical Examination-Weight		

	Safety Analysis Set		
14.3.6.6	Physical Examination-Weight Data outside Clinical Concern Range<br>Safety Analysis Set		
**14.3.7**	**Other Safety**		
14.3.7.1	ECGs<br>Safety Analysis Set		
14.3.7.2	ECG Result Interpretations		
14.3.7.3	ECG Abnormalities for Subjects with Any Abnormal Clinically Significant ECG Interpretation		
14.3.7.4	QTcF Categories<br>Safety Analysis Set		
14.3.7.5	Columbia-Suicide Severity Rating Scale (C-SSRS) at Screening<br>Safety Analysis Set		
14.3.7.6	Columbia-Suicide Severity Rating Scale (C-SSRS) at Post-baseline Visits<br>Safety Analysis Set		
**14.3.8**	**Concomitant Medication**		
14.3.8.1	Concomitant Medications<br>Safety Analysis Set		

**Figure Number**	**Figure Title**	**Validation Method**	**Shell Number (if repeat)**
14.2.1	Mean (+/- SE) Change from baseline in Severe Impairment Battery (SIB) Over Time<br>Full Analysis Set		
14.3.1.1	Box Plot of Change from Baseline in Heart Rate (beats/min)<br>Safety Analysis Set		
14.3.1.2	Box Plot of Change from Baseline in PR (msec)<br>Safety Analysis Set		

Figure Number	Figure Title	Validation Method	Shell Number (if repeat)
14.3.1.3	Box Plot of Change from Baseline in RR (msec)<br>Safety Analysis Set		
14.3.1.4	Box Plot of Change from Baseline in QT (msec)<br>Safety Analysis Set		
14.3.1.5	Box Plot of Change from Baseline in QRS (msec)<br>Safety Analysis Set		
14.3.1.6	Box Plot of Change from Baseline in QTcF (msec)<br>Safety Analysis Set		
14.3.2.1	Box Plot of Change from Baseline in Systolic Blood Pressure (mmHg)<br>Safety Analysis Set		
14.3.2.2	Box Plot of Change from Baseline in Diastolic Blood Pressure (mmHg)<br>Safety Analysis Set		
14.3.2.3	Box Plot of Change from Baseline in Pulse Rate (beats/min)<br>Safety Analysis Set		
14.3.2.4	Box Plot of Change from Baseline in Respiration (breaths/min)<br>Safety Analysis Set		
14.3.2.5	Box Plot of Change from Baseline in Weight (kg)<br>Safety Analysis Set		
14.3.3.1.1	Box Plot of Alanine Aminotransferase (ALT) with an ULN Range<br>Safety Analysis Set		
14.3.3.1.2	Box Plot of Alanine Aminotransferase (ALT) with an LLN Range<br>Safety Analysis Set		
14.3.3.2.1	Box Plot of Aspartate aminotransferase (AST) with an ULN Range		

Figure Number	Figure Title	Validation Method	Shell Number (if repeat)
	Safety Analysis Set		
14.3.3.2.2	Box Plot of Aspartate aminotransferase (AST) with an LLN Range<br>Safety Analysis Set		
14.3.3.3.1	Box Plot of Total Bilirubin with an ULN Range Safety Analysis Set		
14.3.3.3.2	Box Plot of Total Bilirubin with an LLN Range Safety Analysis Set		
14.3.3.4.1	Box Plot of Alkaline Phosphatase with an ULN Range<br>Safety Analysis Set		
14.3.3.4.2	Box Plot of Alkaline Phosphatase with an LLN Range<br>Safety Analysis Set		
14.3.3.5	Box Plot of Creatinine Clearance with an LLN Range<br>Safety Analysis Set		
14.3.4.1	Scatter Plots of LFT Parameters Post-Baseline versus Baseline with ULN Range<br>Safety Analysis Set		
14.3.4.2	Scatter Plot of Maximum ALT versus Maximum Total Bilirubin<br>Safety Analysis Set		
14.3.4.3	Scatter Plot of Maximum AST versus Maximum Total Bilirubin<br>Safety Analysis Set		
14.3.5	Box plot of Baseline CPK of All Safety Subjects versus Those with at Least 1 Myalgia Event<br>Safety Analysis Set		

Listing Number	Listing Title	Validation Method	Shell Number (if repeat)
**16.2**	**Subject Data Listings**		

Listing Number	Listing Title	Validation Method	Shell Number (if repeat)
**16.2.1**	**Discontinued Subjects**		
16.2.1.1	Subject Disposition		
16.2.1.2	Screen Failures		
16.2.1.3	Early Withdrawals		
**16.2.2**	**Protocol Deviations**		
16.2.2.1	Protocol Deviations		
**16.2.3**	**Subjects Excluded From The Efficacy Analyses**		
16.2.3.1	Major Protocol Deviations		
**16.2.4**	**Demographic Data**		
16.2.4.1.1	Subject Randomization		
16.2.4.1.2	Demographic Data		
16.2.4.2.1	Medical History-Probable Alzheimer's Diagnosis		
16.2.4.2.2	Rosen-Modified Hachinski Ischemic Scale and Total Score		
16.2.4.3.1	Previous Medical History		
16.2.4.3.2	Ongoing Medical History		
16.2.4.4	Inclusion Criteria Not Met		
16.2.4.5	Exclusion Criteria Not Met		
16.2.4.6	Prior and Concomitant Medications		
**16.2.5**	**Compliance And/Or Drug Concentration Data**		
16.2.5.1	Infusions and Compliance		
**16.2.6**	**Individual Efficacy Response Data**		
16.2.6.1.1	Severe Impairment Battery (SIB) Scale, Items 1 – 20		
16.2.6.1.2	SIB Scale, Items 21 – 40 and Total Score		
16.2.6.2	Alzheimer Disease Cooperative Study Activities of Daily Living Inventory Severe Impairment Version (ADCS-ADL-SIV) and Total Score		
16.2.6.3	Mini Mental State Examination Version 2 (MMSE-2) and Total Score		
16.2.6.4	Clinical Global Impression of Improvement (CGI-I)		
16.2.6.5	Neuropsychiatric Inventory (NPI) Scale		
**16.2.7**	**Adverse Event Listings**		
16.2.7.1	All Adverse Events		

Listing Number	Listing Title	Validation Method	Shell Number (if repeat)
16.2.7.2	All Serious Adverse Events		
**16.2.8**	**Individual Laboratory Measurements And Other Safety**		
16.2.8.1.1	Hematology Results		
16.2.8.1.2	Chemistry Results		
16.2.8.1.3	Laboratory Liver Function Parameter Abnormalities		
16.2.8.1.4	Other Laboratory Tests		
16.2.8.2	Vital Signs		
16.2.8.3	ECG Parameters		
16.2.8.4	Physical Examination, Abnormal Results		
16.2.8.5	Columbia-Suicide Severity Rating Scale (C-SSRS)		

Approval for implementation of Statistical Analysis Plan

Title:	A Randomized, Double-Blind, Placebo-Controlled, Phase 2 Study Assessing the Safety, Tolerability and Efficacy of Bryostatin in the Treatment of Moderately Severe to Severe Alzheimer's Disease
Protocol Reference:	NTRP-101-202 V4 final 03Mar2017
Sponsor:	Neurotrope BioScience inc.
Issue Date:	22-Mar-2017
Author:	**Shuhua Qi: Senior Biostatistician**
WCT reviewer:	**Jonathan White: Associate Director, Biostatistics**
Author's signature:	[signature] **Date:** 22 Mar 2017
Reviewer's signature:	[signature] **Date:** 22 Mar 2017

The above Statistical Analysis Plan has been reviewed and approved by the Sponsor:

Name of Approver: **Kenneth J. Gorelick, MD FCCP PhD**

Position: **Acting Chief Medical Officer**

Signature: [signature] Date: 22 Mar 2017

Name of Approver: **Elaine Grenier**

Position: **Director, Head of Clinical Operations, Neurotrope BioScience inc.**

Signature: [signature] Date: 22 Mar 2017
